# Hyperglycolysis in endothelial cells drives endothelial injury and microvascular alterations in peritoneal dialysis

**DOI:** 10.1002/ctm2.1498

**Published:** 2023-11-30

**Authors:** Zekun Si, Wenyan Su, Zhuoyu Zhou, Jinjin Li, Cailing Su, Ying Zhang, Zuoyu Hu, Zhijie Huang, Hong Zhou, Ansheng Cong, Zhanmei Zhou, Wei Cao

**Affiliations:** ^1^ Division of Nephrology State Key Laboratory of Organ Failure Research Guangdong Provincial Key Laboratory of Nephrology Guangdong Provincial Clinical Research Center for Kidney Disease Nanfang Hospital Southern Medical University Guangzhou P. R. China; ^2^ Division of Nephrology The Second Affiliated Hospital of Guangzhou Medical University Guangzhou P. R. China

**Keywords:** 6‐phosphofructo‐2‐kinase/fructose‐2,6‐biphosphatase 3, glycolysis, microvascular alterations, peritoneal dialysis, peritoneal endothelial cells

## Abstract

**Background:**

Endothelial cell (EC) dysfunction leading to microvascular alterations is a hallmark of technique failure in peritoneal dialysis (PD). However, the mechanisms underlying EC dysfunction in PD are poorly defined.

**Methods:**

We combined RNA sequencing with metabolite set analysis to characterize the metabolic profile of peritoneal ECs from a mouse model of PD. This was combined with EC‐selective blockade of glycolysis by genetic or pharmacological inhibition of 6‐phosphofructo‐2‐kinase/fructose‐2,6‐biphosphatase 3 (PFKFB3) in vivo and in vitro. We also investigated the association between peritoneal EC glycolysis and microvascular alterations in human peritoneal samples from patients with end‐stage kidney disease (ESKD).

**Results:**

In a mouse model of PD, peritoneal ECs had a hyperglycolytic metabolism that shunts intermediates into nucleotide synthesis. Hyperglycolytic mouse peritoneal ECs displayed a unique active phenotype with increased proliferation, permeability and inflammation. The active phenotype of mouse peritoneal ECs can be recapitulated in human umbilical venous ECs and primary human peritoneal ECs by vascular endothelial growth factor that was released from high glucose‐treated mesothelial cells. Importantly, reduction of peritoneal EC glycolysis, via endothelial deficiency of the glycolytic activator PFKFB3, inhibited PD fluid‐induced increases in peritoneal capillary density, vascular permeability and monocyte extravasation, thereby protecting the peritoneum from the development of structural and functional damages. Mechanistically, endothelial PFKFB3 deficiency induced the protective effects in part by inhibiting cell proliferation, VE‐cadherin endocytosis and monocyte‐adhesion molecule expression. Pharmacological PFKFB3 blockade induced a similar therapeutic benefit in this PD model. Human peritoneal tissue from patients with ESKD also demonstrated evidence of increased EC PFKFB3 expression associated with microvascular alterations and peritoneal dysfunction.

**Conclusions:**

These findings reveal a critical role of glycolysis in ECs in mediating the deterioration of peritoneal function and suggest that strategies targeting glycolysis in peritoneal ECs may be of therapeutic benefit for patients undergoing PD.

## Graphical abstract

1. Peritoneal endothelial cells have a hyperglycolytic metabolism during peritoneal dialysis (PD).
2. Endothelial PFKFB3 deficiency/blockade inhibits PD fluid‐induced peritoneal microvascular alterations, thereby protecting the peritoneum from the development of structural and functional damages.
3. Mechanistically, endothelial PFKFB3 deficiency induces the protective effects in part by inhibiting cell proliferation, VE‐cadherin endocytosis and monocyte‐adhesion molecule expression.

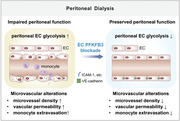

## 1 BACKGROUND

Peritoneal dialysis (PD) is a home‐based kidney replacement therapy option for approximately 4 million patients with end‐stage kidney disease (ESKD).^1^, ^2^ It offers a cost‐effective dialysis treatment with greater individual freedom and better preservation of residual kidney function as compared to haemodialysis.^3^, ^4^ However, PD treatment of ESKD is associated with a gradual loss of efficacy.^5^ Extended exposure of the peritoneum to glucose‐based PD fluids induces progressive mesothelial cell loss, submesothelial fibrosis and microvascular alterations, culminating in ultrafiltration failure.^6^ Microvascular alterations in PD, as defined by increases in microvessel density, vascular permeability and monocyte transendothelial migration, presumably result from endothelial cell (EC) proliferation, inflammation or injury.^7^, ^8^, ^9^, ^10^ However, the underlying mechanisms of EC dysfunction in PD are poorly defined.

ECs, which line the inner vascular wall, act as the primary barrier for solute transport across the vessel wall, and play an essential role in regulating angiogenesis, vascular integrity and monocyte extravasation.^5^, ^10^, ^11^, ^12^ Not unexpectedly, microvascular alterations in PD accelerate solute transfer,^13^, ^14^ facilitate peritoneal inflammation with fibrotic consequences,^15^ and thus contribute to loss of PD efficacy. Despite decades of intense research, less is known about the cues that induce EC dysfunction in PD.^7^ Current vascular normalization strategies focus mainly on targeting angiogenic growth factors, including the vascular endothelial growth factor (VEGF).^16^, ^17^ But blockade of one growth factor would lead to compensatory upregulation of others, which could stimulate vascular changes again.^18^ Thus, the need for a new regulatory system for EC dysfunction during PD has been recognized.

ECs possess unique metabolic properties.^19^ They rely on glycolysis to generate more than 85% of their ATP, even in healthy, quiescent vasculature with abundant oxygen availability.^20^, ^21^ ECs also have heterogeneous phenotypes in different tissues^22^ and are highly plastic, responding differently to changes in the local microenvironment.^18^, ^23^ In limb disorders and tumours, ECs upregulate glycolysis or glycolytic side pathways to fuel local angiogenesis.^24^, ^25^, ^26^ Increased glycolysis under these conditions is mediated by 6‐phosphofructo‐2‐kinase/fructose‐2,6‐biphosphatase 3 (PFKFB3), which generates fructose‐2,6‐bisphosphate to activate the rate‐limiting glycolytic enzyme phosphofructokinase.^20^ However, in the context of PD, it remains unclear if peritoneal ECs possess an altered metabolism, and if targeting the reprogrammed metabolism in ECs provides therapeutic benefit.

Here, we first characterized the metabolic profile of peritoneal ECs in the condition of PD. We then examined the role of glycolysis in peritoneal ECs during peritoneal dysfunction, and investigated if targeting EC glycolysis affects peritoneal vessels and improves peritoneal function in PD. We addressed these issues using in vivo mouse models, in vitro cell models, and human peritoneal tissue samples.

## 2 MATERIALS AND METHODS

Detailed methods used in this study are provided in Supporting Information. All the images selected for publication in each sample represent the sample mean.

### 2.1 Experimental PD model

Animal procedures were approved by the Institutional Animal Ethics Committee (NFYY‐2022‐0227). The floxed *Pfkfb3* (*Pfkfb3* ^flox/flox^) mice and *Cdh5‐Cre* transgenic mice on a C57BL/6J background were obtained from Cyagen Bioscience. Normal C57BL/6J mice were obtained from our Institutional Animal Experiment Center. Male mice aged 8–10 weeks (20–24 g) were used.

**FIGURE 1:**
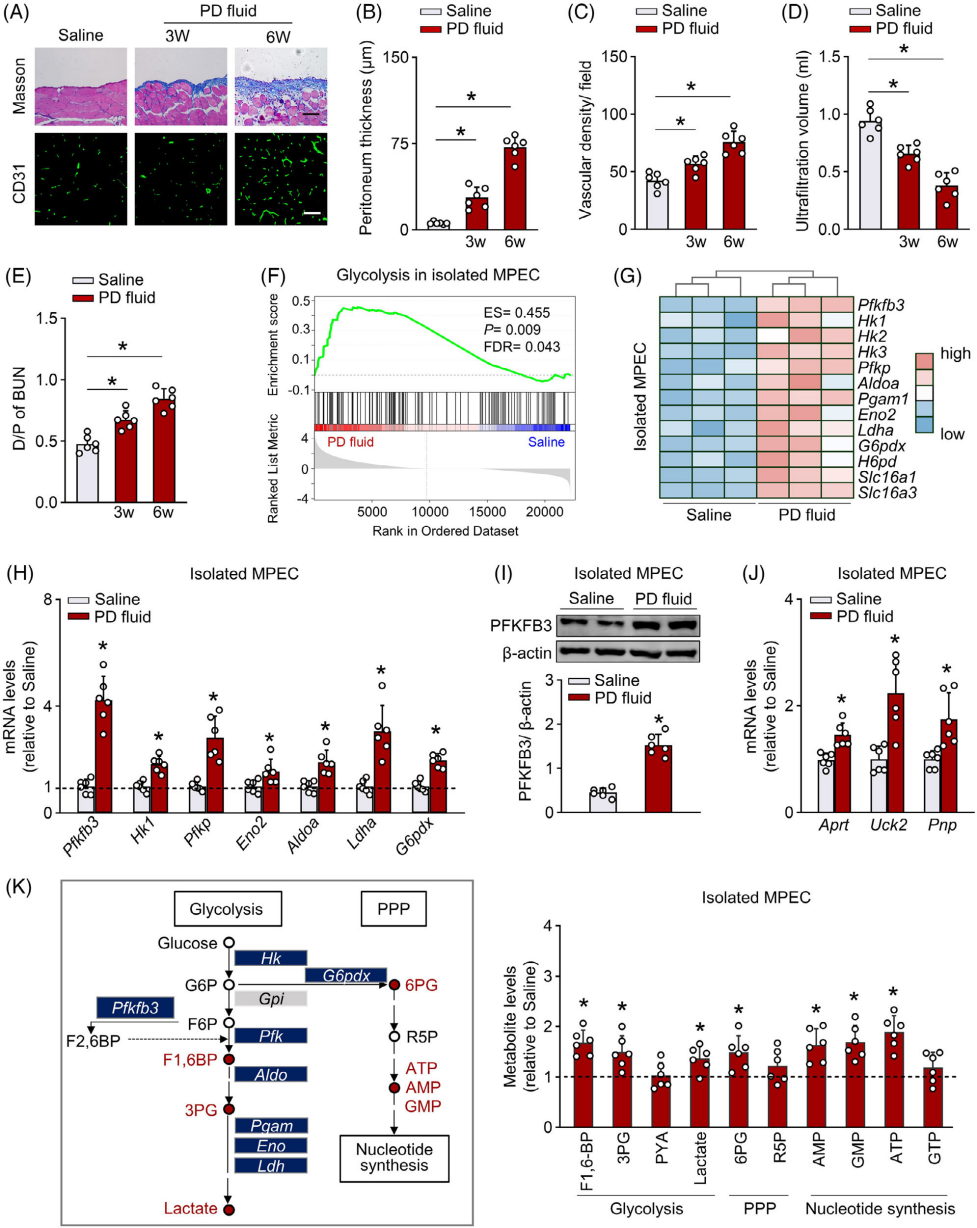
Peritoneal endothelial cells (ECs) are hyperglycolytic in experimental peritoneal dialysis (PD) model. The experimental PD model was induced by daily intraperitoneal injection of 4.25% PD fluid into mice for 3–6 weeks (PD fluid group). Sham‐operated mice received daily intraperitoneal injection of saline for 3–6 weeks (saline group). (A) Peritoneal fibrosis presented by Masson's trichrome staining (Masson; scale bar: 100 μm), and the density of peritoneal microvessels presented by immunofluorescence staining for CD31 (green) in the mesentery (scale bar: 50 μm). (B) The quantitative data of peritoneal thickness detected by Masson staining in A. One‐way ANOVA followed by Bonferroni test; *p < .05. (C) The quantitative data of vascular density in A. One‐way ANOVA followed by Bonferroni test. (D and E) The peritoneal ultrafiltration volume (D) and peritoneal permeability of blood urea nitrogen (D/P of BUN) (E) examined by modified peritoneal equilibration test. One‐way ANOVA followed by Bonferroni test. (F) Mouse peritoneal ECs (MPECs) were isolated from the mesentery of PD fluid and saline mice. Gene set enrichment analysis (GSEA) plots demonstrated enrichment score (ES) for glycolysis pathway in RNA sequencing data of isolated MPECs. FDR, false discovery rate. (G) Heatmap analysis showing transcript levels of glycolysis‐related genes in RNA sequencing data of isolated MPECs. (H) mRNA levels of genes involved in glycolysis and pentose phosphate pathways (PPP) in isolated MPECs. *t*‐test, *p < .05 versus saline group. (I) PFKFB3 protein level in isolated MPECs. *t*‐test, *p < .05 versus saline group. (J) mRNA levels of genes involved in nucleic acid synthesis pathway in isolated MPECs. *t*‐test, *p < .05 versus saline group. (K) Metabolite analysis in isolated MPECs. *t*‐test, *p < .05 versus saline group. Error bars, mean ± SD (*n* = 6 mice in each group). Hk1, hexokinase 1; Pfkfb3, 6‐phosphofructo‐2‐kinase/fructose‐2,6‐biphosphatase 3; Pfkp, phosphofructokinase platelet; Eno2, enolase 2; Aldoa, aldolase A, fructose‐bisphosphate; Ldha, lactate dehydrogenase A; G6pdx, glucose‐6‐phosphate dehydrogenase X‐linked; Aprt, adenine phosphoribosyl transferase; Uck2, uridine‐cytidine kinase 2; Pnp, purine‐nucleoside phosphorylase; F1,6‐BP, 1.6‐fructose diphosphate; 3PG, 3‐phosphoglyceric acid; PYA, pyruvic acid; 6PG, 6‐phosphogluconic acid; R5P, 5‐phosphoribose.

The mouse PD model was induced by intraperitoneally injecting normal mice with 4.25% Dianeal PD fluid (containing 4.25% glucose; Baxter Healthcare; at 100 mL/kg bodyweight) daily for 6 weeks.^27^, ^28^, ^29^ Sham‐operated mice received daily intraperitoneal injection of saline for 6 weeks (saline mice). The PD model was validated by the detection of progressive peritoneal fibrosis and impaired peritoneal function (as indicated by a reduced ultrafiltration volume and increased solute transport rate) during the 6‐week observation period (Figure 1A–E and Figure S1A–C).

For pharmacological inhibition of PFKFB3‐driven glycolysis, PD mice received injection of 25 mg/kg PFKFB3‐blocker 3‐(3‐pyridinyl)‐1‐(4‐pyridinyl)‐2‐propen‐1‐one (3PO)^25^ or DMSO (intraperitonealy, all from Sigma‐Aldrich), three times per week starting from Day 1 or after 3 weeks of PD fluid administration. The efficiency of PFKFB3 inhibition by 3PO was confirmed by reduced generation of F2,6‐P2 in peritoneal ECs isolated from 3PO‐treaed mice (Figure S6B).

### 2.2 Generation of endothelial‐specific *Pfkfb3* knockout mice

To obtain endothelial‐specific *Pfkfb3*‐knockout (*Pfkfb3* ^ΔEC^) mice, *Pfkfb*3^flox/flox^ (*Pfkfb3* ^WT^) mice were intercrossed with *Cdh5‐Cre* transgenic mice, an EC‐selective Cre‐driver line (all from Cyagen Bioscience).^23^, ^30^, ^31^ Consistent with previous studies,^23^, ^30^, ^31^ *Pfkfb3* ^ΔEC^ mice were healthy and fertile. And there was no significant difference in baseline bodyweight (*Pfkfb3* ^WT^ vs. *Pfkfb3* ^ΔEC^ mice: 22.2 ± 1.1 vs. 20.8 ± 2.6) and peritoneal microvessel density (Figure 4C) between *Pfkfb3* ^WT^ and *Pfkfb3* ^ΔEC^ mice.

### 2.3 Cell isolation and culture

Mouse peritoneal ECs and mesothelial cells were isolated from mouse mesentery as described in Supporting Information Methods. The identity of the mesentery is confirmed by the detection of UPK3B expression (a marker of mesothelial cells) using immunostaining and immunoblot analysis (Figure S1D,E). Primary human parietal peritoneal endothelial cells (HPECs, human) were purchased from Procell, and human umbilical vein endothelial cells (HUVECs, human) and mesothelial cells (Met‐5A, human) were purchased from American Type Culture Collection. These cells were cultured and used for experiments as described in Supporting Information Methods.

### 2.4 Human peritoneal samples

Studies using human peritoneal samples (the omentum) were approved by Ethics Committee in the Second Affiliated Hospital of Guangzhou Medical University and conducted in accordance with the Declaration of Helsinki (2021‐hs‐61). Omentum tissue from ESKD patients on PD was collected during PD catheter removal due to ultrafiltration failure (*n* = 3 patients). Omentum tissue from age‐ and serum creatinine‐matched ESKD patients was obtained during PD catheter insertion (*n* = 3 patients). Patient characteristics are presented in Table S1.

### 2.5 RNA sequencing

For RNA sequencing, peritoneal ECs were isolated from three randomly selected saline mice and three randomly selected PD mice. Total RNA was isolated from cells using TRIzol reagent (Invitrogen). RNA quality check and sequencing were performed on the Agilent 2100 Bioanalyzer and the Illumina NovaSeq 6000 platform. Heatmap analysis was performed using the pheatmap package, and differential expression genes (DEGs) were calculated with the Limma package. Gene set enrichment analysis (GSEA) was performed using the clusterProfiler package. RNA‐sequencing data are available in the GSE database under accession number GSE230008.

### 2.6 Statistical analysis

Quantitative data are shown as means and standard deviations, and analyzed with SPSS 20.0 (SPSS). Shapiro–Wilk statistics were used for normality tests. Differences between two groups were assessed by an unpaired Student *t*‐test, while differences among groups by ANOVA or unpaired *t*‐test with Bonferroni correction for multiple testing. A *p*‐value of less than .05 was considered statistically significant.

## 3 RESULTS

### 3.1 Peritoneal ECs are hyperglycolytic in experimental PD model

To investigate the metabolic profile of peritoneal ECs in response to PD, we first prepared an experimental PD model by daily peritoneal instillation of human Dianeal PD fluid containing 4.25% glucose for 6 weeks. The PD mice had a progressive peritoneal fibrosis from 3 to 6 weeks, characterized by a gradual increase in peritoneal thickness and overexpression of extracellular matrix in peritoneal tissue (Figure 1A,B and Figure S1A,B). Importantly, the density of peritoneal microvessels, identified by the endothelial marker CD31, was significantly increased in PD mice from 3 to 6 weeks (Figure 1A,C). Consistent with these histological data, a reduced ultrafiltration volume, and increased peritoneal permeability of glucose and blood urea nitrogen were observed in PD mice (Figure 1D,E and Figure S1C), confirming an impaired peritoneal function in this PD model.

We then isolated mouse peritoneal ECs from the mesentery of PD and saline mice (Figure S2A), and performed RNA sequencing to characterize their metabolic profile. GSEA revealed that glycolysis was one of the most enriched gene sets associated with peritoneal dysfunction (Figure 1F and Figure S2B). A similar trend was also observed for nucleotide metabolism, amino acid metabolism and carbohydrate metabolism (Figure S2B). Pathway mapping and heatmap analysis demonstrated that peritoneal ECs from PD mice had upregulated transcript levels of genes involved in glycolysis, including PFKFB3, hexokinase 1 (HK1), the pentose phosphate pathway (such as glucose‐6‐phosphate dehydrogenase [G6pdx], hexose‐6‐phosphate dehydrogenase [H6pd]) (Figure 1G), nucleotide metabolism, amino acid metabolism and carbohydrate metabolism (Figure S2C). Further real‐time PCR and immunoblot analyses confirmed upregulation of glycolytic enzymes in peritoneal ECs from PD mice (Figure 1H,I and Figure S2D,E). Importantly, PFKFB3 transcript levels were more than three‐fold higher in peritoneal ECs from PD mice than those from saline mice, while expression of other glycolytic enzymes was moderately altered (Figure 1H). In agreement with this increase in glycolysis, expression of enzymes involved in nucleotide synthesis was also upregulated in peritoneal ECs from PD mice (Figure 1J). Therefore, PD fluid treatment might increase glycolysis in peritoneal ECs to support biomass production and proliferation.

To confirm this notion, we assayed stable intermediate metabolites of glycolysis, pentose phosphate pathway and nucleotide synthesis pathways in mouse peritoneal ECs using liquid chromatography‐mass spectrometry (LC‐MS). As shown in Figure 1K, peritoneal ECs from PD mice (vs. saline mice) had higher levels of intermediate metabolites involved in these pathways (*p* < .05). Further investigation revealed upregulated glucose consumption and lactate excretion in the medium of peritoneal ECs from PD mice (Figure S2F).

Together, our data show that the development of peritoneal dysfunction during PD is associated with hyperglycolysis in peritoneal ECs, rendering glycolysis in peritoneal ECs an attractive target.

### 3.2 Soluble factors from mesothelial cells induce hyperglycolysis in ECs

Next, we investigated the drivers of glycolysis in ECs during peritoneal dysfunction. Direct treatment with high glucose or mannitol for 24 h, at concentrations equivalent to human PD fluid, did not significantly increase cell viability or glycolysis‐related gene expression in HUVECs (Figure S4A–D). To explore which cell populations are able to drive EC glycolysis, we analyzed and annotated single‐cell RNA sequencing (scRNA‐seq) data of human peritoneal cells from patients on PD.^27^ As shown in Figure S3A, nine cell clusters were derived, covering diverse cell types in the peritoneum. Among the cell populations, mesothelial cells exhibited the highest enrichment score of angiogenesis‐related gene sets, and this increased further after long‐term PD (Figure S3B,C), highlighting the mesothelial–endothelial crosstalk under PD condition. We therefore incubated HUVECs with the conditioned medium from human mesothelial cells that were exposed to high glucose (1.5%, a concentration equivalent to 1.5% human PD fluid; HG‐CM) or normal glucose (.1%, NG‐CM), which mimics the in vivo peritoneal niche in PD or saline mice.

**FIGURE 2:**
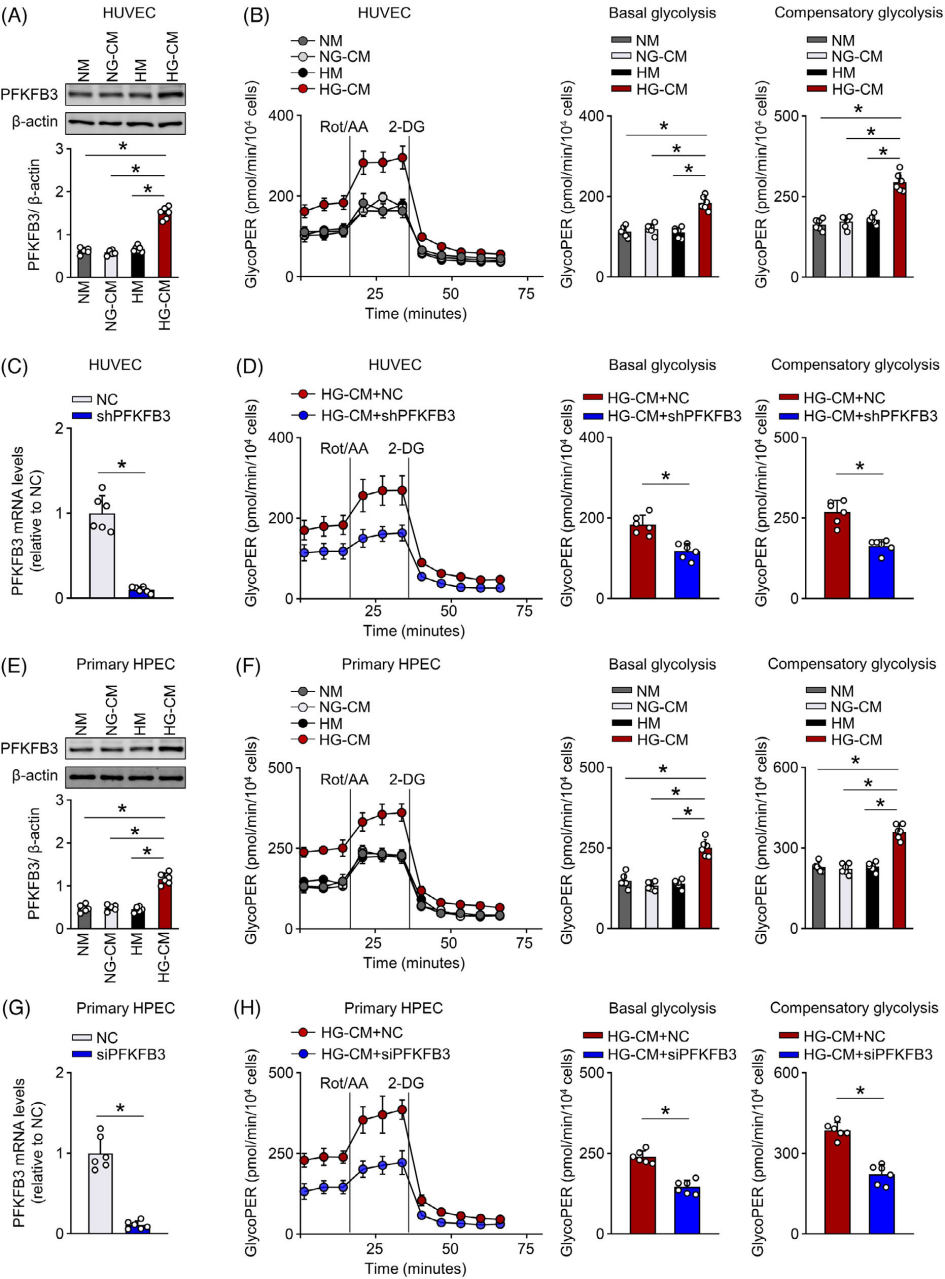
Soluble factors from mesothelial cells induces hyperglycolysis in endothelial cells (ECs). We incubated human umbilical venous ECs (HUVECs) and primary human peritoneal ECs (HPECs) with the conditioned medium from human mesothelial cells (Met‐5A) that were exposed to 1.5% high glucose (HG‐CM) or .1% glucose (NG‐CM), which mimics the in vivo peritoneal niche in peritoneal dialysis (PD) fluid or saline mice. NM, normal medium. HM, medium containing 1.5% glucose. (A) PFKFB3 protein levels in HUVECs. One‐way ANOVA followed by Bonferroni test; *p < .05. (B) Glycolytic rate (termed GlycoPER) measured by glycolytic rate assay in HUVECs. One‐way ANOVA followed by Bonferroni test; *p < .05. (C) PFKFB3 mRNA levels in HUVECs transfected with shPFKFB3 or negative control (NC). *t*‐test; *p < .05. (D) Knockdown of PFKFB3 in HUVECs markedly reduced the HG‐CM‐induced increase in glycolytic rate. *t*‐test; *p < .05. (E) PFKFB3 protein levels in HPECs. One‐way ANOVA followed by Bonferroni test; *p < .05. (F) Glycolytic rate (termed GlycoPER) in HPECs. One‐way ANOVA followed by Bonferroni test; *p < .05. (G) PFKFB3 mRNA levels in HPECs transfected with siPFKFB3 or NC. *t*‐test; *p < .05. (H) Knockdown of PFKFB3 in HPECs markedly reduced HG‐CM‐induced increase in glycolytic rate. *t*‐test; *p < .05. Error bars, mean ± SD (*n* = 6 cell samples in each group).

HG‐CM treatment (vs. NG‐CM) for 24 h induced a marked increase in cell viability and the mRNA levels of key glycolysis‐related enzymes, especially PFKFB3 (Figure S4F,G). Changes in the protein level of PFKFB3 paralleled the mRNA level (Figure 2A). We then assessed the glycolytic function of HG‐CM‐treated ECs by glycolytic rate assay and glycolytic stress test using a Seahorse extracellular flux analyzer. HUVECs incubated with HG‐CM (vs. NG‐CM) had a greater increase in glycolytic rate (termed GlycoPER) and glucose‐induced extracellular acidification rate (ECAR), indicating increased activity of glycolytic metabolism (Figure 2B and Figure S4H). Interestingly, knockdown of PFKFB3 in HUVECs markedly reduced the upregulation of EC glycolysis by HG‐CM (Figure 2C,D and Figure S4I). Thus, soluble factor(s) released from mesothelial cells in the setting of PD, can induce glycolytic enzyme expression and promote glycolysis in adjacent ECs.

We also examined the effect of HG‐CM on primary human peritoneal ECs (HPECs). As shown in Figure 2E–H, incubation of HPECs with HG‐CM upregulated glycolysis as evidenced by an increase in glycolytic rate and PFKFB3 expression, whereas knockdown of PFKFB3 in HPECs reduced the hyperglycolysis induced by HG‐CM.

Incubation of both ECs with a high glucose medium (HM, 1.5% glucose) did not significantly alter glycolysis as detected by glycolytic rate assay (Figure 2B,F). Together, soluble cytokines released from mesothelial cells are potent factors for inducing PFKFB3‐mediated hyperglycolysis in ECs, reinforcing that EC glycolysis is involved in the development of peritoneal dysfunction.

### 3.3 PFKFB3 controls glycolysis in peritoneal ECs

**FIGURE 3:**
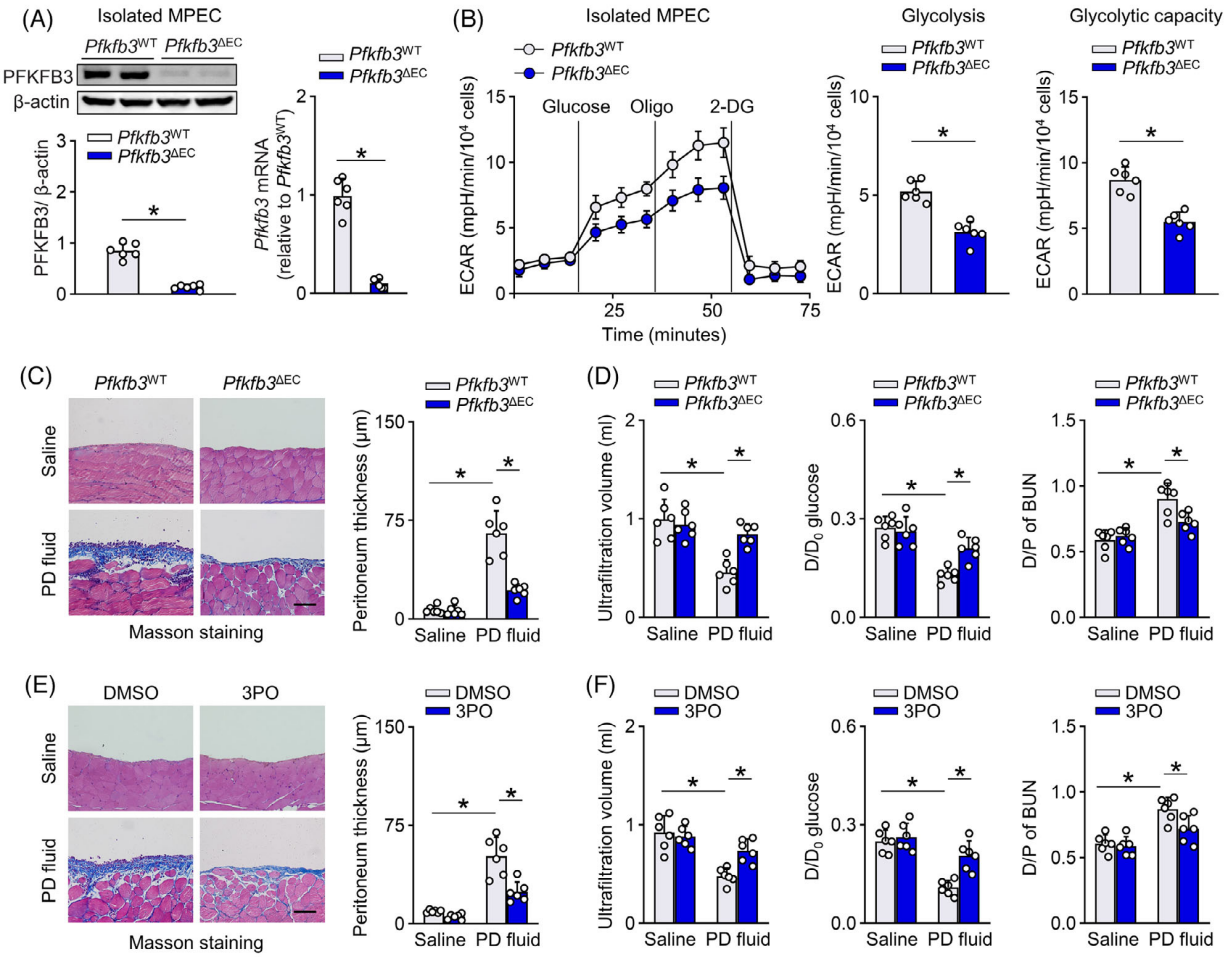
Genetic or pharmacological inhibition of endothelial cell (EC) PFKFB3 lessens peritoneal fibrosis and improves peritoneal function in peritoneal dialysis (PD) model. (A and B) EC‐specific PFKFB3 knockout mice (*Pfkfb3* ^ΔEC^) was generated by intercrossing *Cdh5*‐Cre mice with *Pfkfb3* ^flox/flox^ (*Pfkfb3* ^WT^) mice. A markedly reduced protein and transcript level of PFKFB3 was observed in isolated MPECs from *Pfkfb3* ^ΔEC^ mice as compared to *Pfkfb3* ^WT^ mice (A). The PFKFB3‐deficient MPECs had a lower glucose‐induced elevation in extracellular acidification rate (ECAR) as detected by glycolytic stress assay (B). *t*‐test; *p < .05. (C and D) Both *Pfkfb3* ^WT^ and *Pfkfb3* ^ΔEC^ mice received daily intraperitoneal injection of PD fluid for 6 weeks. As compared to *Pfkfb3* ^WT^ mice, *Pfkfb3* ^ΔEC^ mice had a significantly reduced PD fluid‐induced peritoneal fibrosis (indicated by Masson staining; C) and improved peritoneal function (indicated by ultrafiltration volume, D/D_0_ glucose, D/P of BUN; D). Scale bar: 100 μm. One‐way ANOVA followed by Bonferroni test; *p < .05. (E and F) Pharmacological inhibition of PFKFB3 was achieved by intraperitoneal injection of mice with 3PO three times per week starting from 1 day after PD fluid treatment. 3PO administration significantly reduced PD fluid‐induced peritoneal fibrosis (E) and improved peritoneal function (F). Scale bar: 100 μm. One‐way ANOVA followed by Bonferroni test; *p < .05. Error bars, mean ± SD (*n* = 6 mice in each group).

To examine if targeting PFKFB3 regulates glycolysis in peritoneal ECs in vivo, we generated EC‐specific PFKFB3 knockout mice (*Pfkfb3* ^ΔEC^) by intercrossing *Cdh5‐Cre* mice with *Pfkfb3* ^flox/flox^ (*Pfkfb3* ^WT^) mice.^23^, ^30^, ^31^ We observed a markedly reduced PFKFB3 expression in peritoneal ECs from *Pfkfb3* ^ΔEC^ mice as compared to *Pfkfb3* ^WT^ mice (Figure 3A), confirming effective deletion of endothelial PFKFB3 in *Pfkfb3* ^ΔEC^ mice. Seahorse flux analysis revealed that PFKFB3‐deficient mouse peritoneal ECs (vs. *Pfkfb3* ^WT^ ECs) had a reduced ECAR (Figure 3B), supporting a key role of PFKFB3 in controlling glycolysis in peritoneal ECs.

### 3.4 Genetic or pharmacological inhibition of EC PFKFB3 lessens peritoneal fibrosis and improves peritoneal function in PD model

To investigate the role of EC glycolysis in development of peritoneal dysfunction during PD, both *Pfkfb3* ^WT^ and *Pfkfb3* ^ΔEC^ mice received daily intraperitoneal injection of PD fluid for 6 weeks. PD fluid (vs. saline) caused significant thickening of parietal peritoneal tissue and increased peritoneal expression of extracellular matrix in *Pfkfb3* ^WT^ mice. However, these PD fluid‐induced effects were clearly attenuated in *Pfkfb3* ^ΔEC^ mice (Figure 3C and Figure S5A). Peritoneal function, as reflected by solute transport rate and ultrafiltration volume, was also improved in *Pfkfb3* ^ΔEC^ mice (Figure 3D).

To mimic the PFKFB3 deficiency, we used a low dose of PFKFB3‐blocker 3PO (25 mg/kg; three times per week), which has been reported to reduce EC glycolysis in vivo.^25^ Peritoneal fibrosis was assessed by Masson's trichrome staining and extracellular matrix expression. Figure 3E shows images of the stained peritoneal tissue sections, in which deposited extracellular matrix is stained blue. Treatment with 3PO in PD mice, initiated from the first day of PD fluid administration, attenuated PD fluid‐induced peritoneal fibrosis and improved peritoneal function at Week 6 (Figure 3E,F and Figure S5B). Furthermore, 3PO treatment, initiated after 3 weeks of PD fluid administration also reduced peritoneal extracellular matrix expression and improved peritoneal function at Week 6 (Figure S5C–E).

Taken together, these data identify hyperglycolysis in ECs as a critical participant in the development of peritoneal dysfunction during PD. Blocking glycolysis in peritoneal ECs by PFKFB3 inhibition helps to impede the development of peritoneal dysfunction.

### 3.5 Genetic inhibition of EC PFKFB3 inhibits endothelial proliferation and reduces peritoneal microvessel density in PD model

**FIGURE 4:**
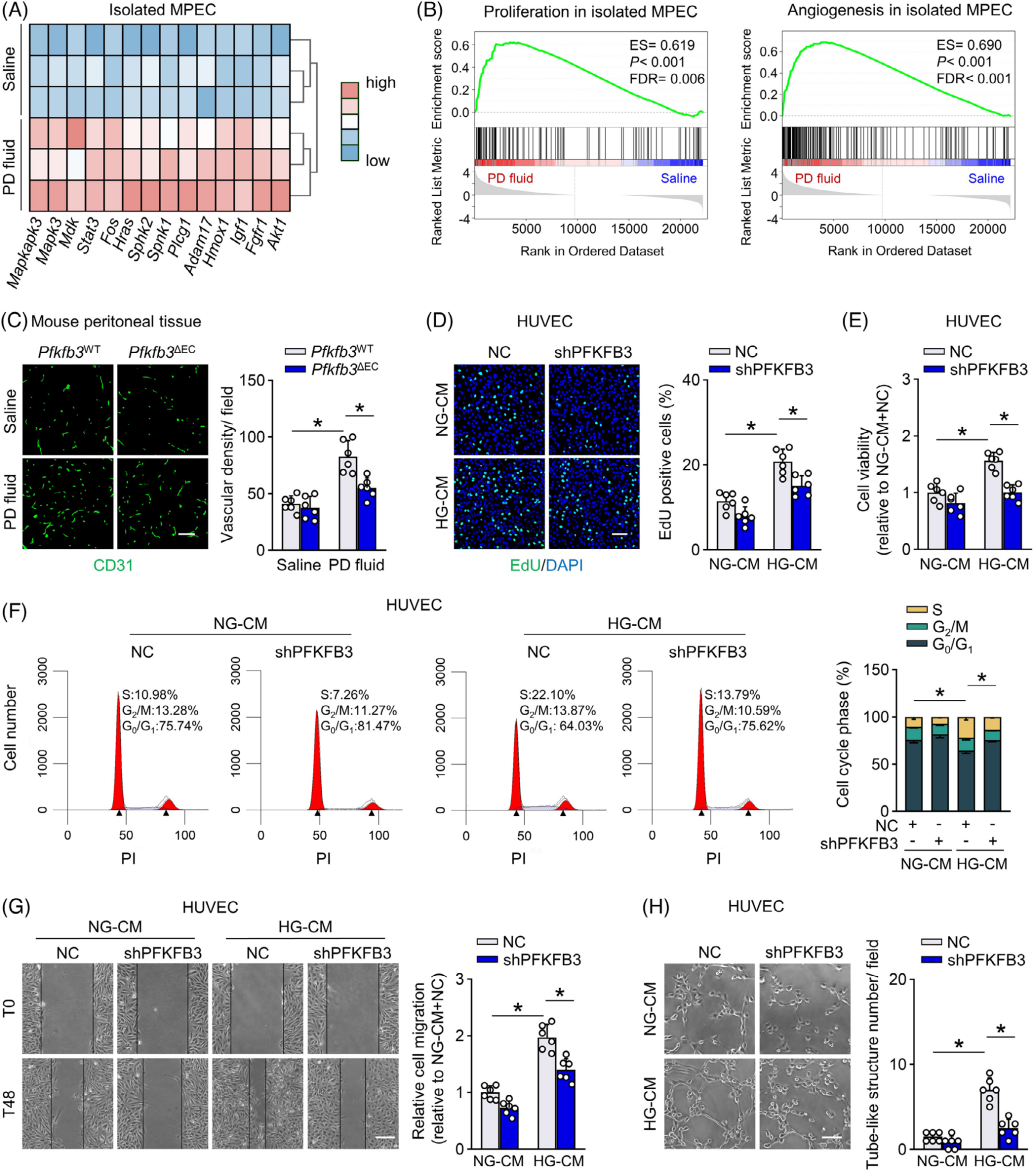
Genetic inhibition of endothelial cell (EC) PFKFB3 inhibits endothelial proliferation and reduces peritoneal microvessel density in peritoneal dialysis (PD) model. (A) Heatmap analysis showing transcript levels of proliferation and angiogenesis‐related genes in RNA sequencing data of isolated MPECs from saline‐ and PD fluid‐treated mice. (B) Gene set enrichment analysis (GSEA) plots demonstrating ES for proliferation and angiogenesis pathways in RNA sequencing data of isolated MPECs. (C) Immunofluorescence staining for CD31 (green) in the mesentery from *Pfkfb3* ^WT^ and *Pfkfb3* ^ΔEC^ mice treated with PD fluid or saline. Quantitative data showing the vascular density/field. Scale bar: 50 μm. One‐way ANOVA followed by Bonferroni test; *p < .05. (D) Human umbilical venous ECs (HUVECs) transfected with shPFKFB3 or negative control (NC) were incubated with HG‐CM for 24 h. EdU incorporation images and quantitative data (percentage of EdU‐positive cells) are shown. Scale bar: 100 μm. One‐way ANOVA followed by Bonferroni test; *p < .05. (E) CCK‐8 assay showing cell viability in HUVECs. One‐way ANOVA followed by Bonferroni test; *p < .05. (F) Flow cytometry showing distribution of different cell cycle phases in HUVECs: flow cytometry histograms and quantitative data. One‐way ANOVA followed by Bonferroni test; *p < .05. (G) Scratch wound migration assay showing migration of HUVECs. Quantitative data showing the migration distance at the time of scratching (T0) and 48 h after scratching (T48). Scale bar: 200 μm. One‐way ANOVA followed by Bonferroni test; *p < .05. (H) Tube formation assay showing tube formation of HUVECs: images and quantitative data (tube‐like structure number/field). Scale bar: 100 μm. One‐way ANOVA followed by Bonferroni test. *p < .05. Error bars, mean ± SD (*n* = 6 mice in each group in C, *n* = 6 cell samples in D–H).

We next investigated why inhibition of PFKFB3‐mediated glycolysis in ECs protects the peritoneum from PD fluid‐induced damage. Peritoneal microvessel density is recognized as a major determinant of peritoneal solute transport in PD.^14^ We explored if PFKFB3 deficiency alters peritoneal microvessel density in PD model. In *Pfkfb3* ^WT^ mice, PD fluid (vs. saline) increased expression of genes involved in proliferation, migration and angiogenesis in mouse peritoneal ECs (Figure 4A,B and Figure S6A), and increased microvessel density (marked by CD31) in the peritoneum (Figure 4C). Notably, the PD fluid‐induced effect was clearly attenuated in *Pfkfb3* ^ΔEC^ mice and 3PO‐treated mice (Figure 4C and Figure S6C). Thus, PFKFB3‐driven glycolysis in ECs contributes to PD‐induced increase in peritoneal microvessel density.

We next examined the effect of HG‐CM, a scenario similar to PD, on primary HPECs. HG‐CM administration increased cell proliferation, as indicated by elevations in EdU (5‐ethynyl‐2′‐deoxyuridine)‐positive nuclei ratio and cell viability (Figure S6D,E). Importantly, knockdown of PFKFB3 in HPECs reduced these HG‐CM‐induced effects (Figure S6D,E), linking PFKFB3‐mediated glycolysis to EC proliferation in PD.

Cultured HUVECs were then used to investigate the PFKFB3 function further. Mechanistically, in addition to generating more energy (Figure S6L), incubation of HUVECs with HG‐CM enhanced the binding of PFKFB3 to the cyclin‐dependent kinase (CDK) 4 (Figure S6F), forming a complex^32^, ^33^ that upregulates the expression of the G1/S transition dominator CDK4 (Figure S6G). Indeed, HUVECs treated with HG‐CM had a marked increase in percentage of cells in S‐phase of the cell cycle (Figure 4F), accompanied by elevation in EdU‐positive nuclei ratio and cell viability (Figure 4D,E). Consistently, HG‐CM enhanced in vitro migration, and increased formation of tube‐like structures in HUVECs (Figure 4G,H). Knockdown of PFKFB3 in HUVECs reversed the ability of HG‐CM to upregulate CDK4 (Figure S6G) and its ability to increase EC proliferation, migration and tube formation (Figure 4D–H).

In addition, knockdown of PFKFB3 did not affect nitric oxide generation in HUVECs (Figure S6H). Collectively, activation of EC PFKFB3 links glucose metabolism with peritoneal microvessel density. Blockade of PFKFB3 in ECs protects the peritoneum against angiogenesis during PD.

### 3.6 Genetic inhibition of EC PFKFB3 downregulates vascular endothelial‐cadherin expression and decreases vascular permeability in PD model

**FIGURE 5:**
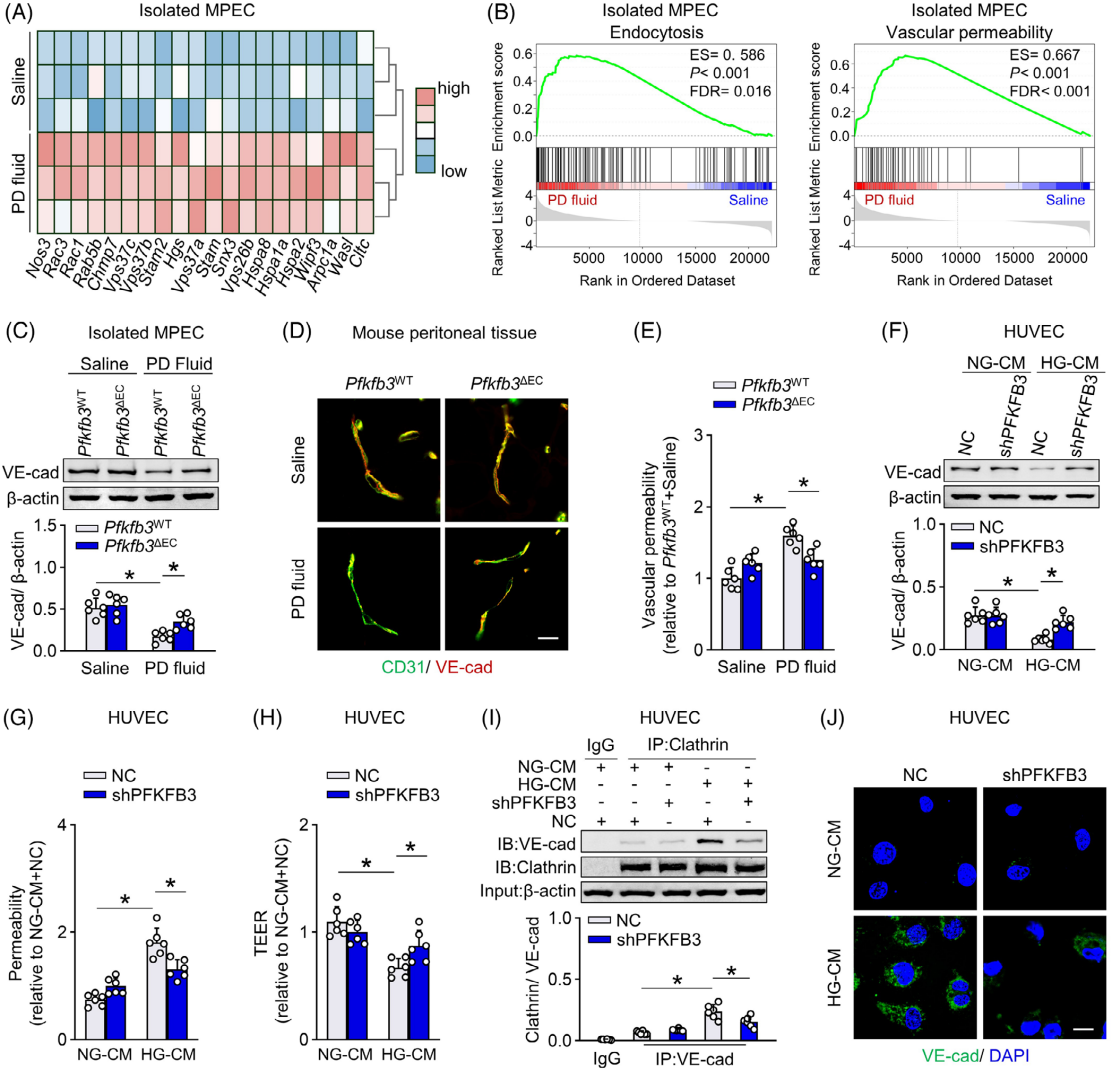
Genetic inhibition of endothelial cell (EC) PFKFB3 restores vascular endothelial‐cadherin endocytosis and reduced vascular permeability in peritoneal dialysis (PD) model. (A) Heatmap analysis showing transcript levels of endocytosis and vascular permeability‐related genes in RNA sequencing data of isolated MPECs from saline and PD fluid mice. (B) Gene set enrichment analysis (GSEA) plots demonstrating ES for endocytosis and vascular permeability pathways in RNA sequencing data of isolated MPECs. (C) VE‐cadherin (VE‐cad) protein level in MPECs isolated from *Pfkfb3* ^WT^ and *Pfkfb3* ^ΔEC^ mice treated with PD fluid or saline. One‐way ANOVA followed by Bonferroni test; *p < .05. (D) Immunostaining of mouse mesentery sections for CD31 (green) and VE‐cad (red). Scale bar: 20 μm. (E) Peritoneal vascular permeability assessed by quantification of Evans blue dye extravasations. One‐way ANOVA followed by Bonferroni test; *p < .05. (F) Human umbilical venous ECs (HUVECs) transfected with shPFKFB3 or negative control (NC) were incubated with HG‐CM for 24 h. VE‐cad protein level in HUVECs are shown. One‐way ANOVA followed by Bonferroni test; *p < .05. (G) In vitro permeability assay using FITC‐dextran demonstrating permeability of HUVECs. One‐way ANOVA followed by Bonferroni test; *p < .05. (H) Transendothelial electrical resistance (TEER) of HUVECs. One‐way ANOVA followed by Bonferroni test; *p < .05. (I) Immunoprecipitation and immunoblot analysis of the binding of Clathrin to VE‐cad in HUVECs. One‐way ANOVA followed by Bonferroni test; *p < .05. (J) Internalized VE‐cad (green) in HUVECs. Scale bar: 20 μm. Error bars, mean ± SD (*n* = 6 mice in each group in C and E; *n* = 6 cell samples in F–I).

As disrupted EC barrier increases vascular permeability that contributes to accelerated peritoneal solute transfer in PD,^34^ we investigated the integrity of the EC barrier in the experimental PD model. GSEA analysis of transcripts in mouse peritoneal ECs revealed that endocytosis and vascular permeability were two of the most enriched gene sets associated with peritoneal dysfunction (Figure 5A,B), supporting an apparent role of disrupted EC barrier in PD‐induced peritoneal dysfunction. Vascular endothelial‐cadherin (VE‐cadherin) is a critical EC adherens junction involved in maintenance of the EC barrier.^34^, ^35^ In *Pfkfb3* ^WT^ mice, PD fluid (vs. saline) decreased the expression of the junctional protein VE‐cadherin in peritoneal ECs (Figure 5C). This PD fluid‐induced downregulation of VE‐cadherin was markedly improved in *Pfkfb3* ^ΔEC^ mice (Figure 5C). Immunostaining of peritoneal sections for CD31 and VE‐cadherin revealed that VE‐cadherin‐positive junctions were more abundant and strongly stained in PD fluid‐treated *Pfkfb3* ^ΔEC^ mice compared with *Pfkfb3* ^WT^ mice (Figure 5D). Accordingly, peritoneal vascular permeability, assessed by quantification of Evans blue dye extravasation,^35^ was increased by PD fluid in *Pfkfb3* ^WT^ mice, but not significantly or to a lesser extent in *Pfkfb3* ^ΔEC^ mice (Figure 5E). Thus, a tightened EC barrier contributes to improved peritoneal function in *Pfkfb3* ^ΔEC^ mice.

Consistently, HG‐CM administration reduced the VE‐cadherin expression in both HUVECs and primary HPECs (Figure 5F and Figure S6I). HG‐CM also increased penetration of FITC‐conjugated dextran across ECs (Figure 5G and Figure S6J) and decreased the trans‐EC electrical resistance (TEER, Figure 5H and Figure S6K), a measure of EC barrier tightness. Knockdown of PFKFB3 in these ECs reversed the HG‐CM‐induced reduction in VE‐cadherin and improved EC barrier tightness (Figure 5F–H and Figure S6I–K).

As VE‐cadherin disruption is largely regulated by Clathrin‐mediated endocytosis^34^ and this process is ATP‐dependent,^36^ we propose that glycolysis provides ATP required for VE‐cadherin endocytosis in ECs during PD. Indeed, incubation of HUVECs with HG‐CM increased ATP generation and promoted the binding of Clathrin to VE‐cadherin, whereas PFKFB3 deficiency decreased ATP generation and induced disassociation of VE‐cadherin from Clathrin (Figure 5I and Figure S6L). Internalized VE‐cadherin was further determined by acid washing HUVECs incubated with anti‐VE‐cadherin antibody and chloroquine.^37^ Knockout of PFKFB3 in HUVECs reversed the HG‐CM‐induced increase in VE‐cadherin endocytosis (Figure 5J). We also found that HG‐CM administration in HUVECs increased the generation of reactive oxygen species (ROS) (Figure S6M), a well‐established disruptor of adherens junctions.^26^, ^38^ PFKFB3 deficiency reduced HG‐CM‐induced increase in ROS generation (Figure S6M) while improving EC barrier tightness (Figure 5H), supporting a role for ROS in VE‐cadherin disruption in the context of PD.

The 1.5% glucose or mannitol did not significantly affect the EC barrier tightness (Figure S6N). In conclusion, glycolysis in ECs mediates PD fluid‐induced EC barrier disruption and vascular hyperpermeability. The protective action of EC PFKFB3 deficiency in PD model is mediated, at least in part, by the preservation of the VE‐cadherin expression.

### 3.7 Genetic inhibition of EC PFKFB3 attenuates endothelial inflammation and alleviates monocyte extravasation in PD model

**FIGURE 6:**
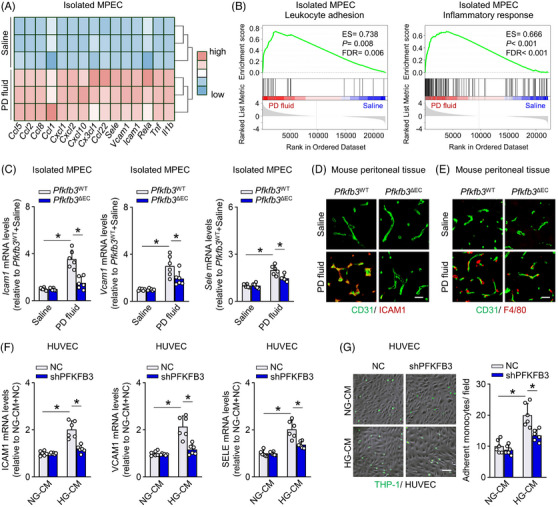
Genetic inhibition of endothelial cell (EC) PFKFB3 attenuates endothelial inflammation and alleviates monocyte extravasation in peritoneal dialysis (PD) model. (A) Heatmap analysis showing transcript levels of adhesion and inflammatory response‐related genes in RNA sequencing data of isolated MPECs from saline and PD mice. (B) Gene set enrichment analysis (GSEA) plots demonstrating ES for leukocyte adhesion and inflammatory response pathways in RNA sequencing data of isolated MPECs. (C) mRNA levels of *Icam1*, *Vcam1* and *Sele* in isolated MPECs from *Pfkfb3* ^WT^ and *Pfkfb3* ^ΔEC^ mice treated with PD fluid or saline. One‐way ANOVA followed by Bonferroni test; *p < .05. (D) Immunostaining of mouse mesentery sections for CD31 (green) and ICAM1 (red). Scale bar: 20 μm. (E) Immunostaining of mesentery sections for CD31 (green) and F4/80 (red) in mouse mesentery sections. Scale bar: 20 μm. (F) mRNA levels of ICAM1, VCAM1 and SELE in shPFKFB3‐ or NC‐transfected human umbilical venous ECs (HUVECs) after incubation with HG‐CM. One‐way ANOVA followed by Bonferroni test; *p < .05. (G) Images and quantification of monocyte (THP1 cell, green) adhesion on HUVECs. Scale bar: 100 μm. One‐way ANOVA followed by Bonferroni test; *p < .05. Error bars, mean ± SD (*n* = 6 mice in each group in C; *n* = 6 cell samples in F and G).

ECs have been implicated in peritoneal inflammation and fibrosis, which impairs peritoneal ultrafiltration,^13^ by allowing transmigration of monocytes into the peritoneum.^10^, ^15^ We examined if ECs exploit their metabolic changes to engage in process of monocyte extravasation in PD. In normal mice, heatmap analysis of transcripts in peritoneal ECs revealed that PD fluid (vs. saline) induced an active EC phenotype, with clustering of genes involved in leukocyte chemotaxis and adhesion (Figure 6A). GSEA analysis confirmed the significant enrichment of leukocyte adhesion and inflammatory response in the diseased mouse peritoneal ECs (Figure 6B). Validation of key molecules involved in monocyte extravasation revealed an upregulation of intercellular adhesion molecule‐1 (*Icam1*), vascular cell adhesion molecule‐1 (*Vcam1*) and E‐selectin (*Sele*) in mouse peritoneal Ecs from PD fluid‐treated *Pfkfb3* ^WT^ mice (Figure 6C and Figure S7A). Immunostaining of peritoneal sections for CD31 and ICAM1 confirmed that ICAM1 expression was markedly increased in peritoneal ECs from PD fluid‐treated *Pfkfb3* ^WT^ mice (Figure 6D). As a consequence, F4/80‐positive macrophages were increased and accumulated in and around peritoneal vessels, close to fibroblasts and mesothelial cells in PD fluid‐treated *Pfkfb3* ^WT^ mice (Figure 6E and Figure S8C,D). Importantly, all of these PD fluid‐induced changes were attenuated in *Pfkfb3* ^ΔEC^ mice (Figure 6C–E, Figures S7A and S8).

In agreement, incubation of HG‐CM with primary HPECs enhanced EC inflammation as detected by ICAM and VCAM expression, whereas knockdown of PFKFB3 in HPECs reversed the ability of HG‐CM to increase EC inflammation (Figure S7B). Mechanically, HG‐CM in HUVECs activated NF‐κB signalling (Figure S7C), a known transcriptional activator of adhesion molecules that could be stimulated by glycolytic product lactate.^25^ Indeed, HG‐CM upregulated the expression of above‐mentioned adhesion molecules (Figure 6F and Figure S7D), and increased the number of monocytes adhering to and migrating across ECs (Figure 6G). Knockdown of PFKFB3 in HUVECs reversed all the effects induced by HG‐CM (Figure 6F,G and Figure S7C,D).

Additionally, 1.5% glucose or mannitol did not significantly alter the EC adhesion molecule expression (Figure S7E). Together, enhanced glycolysis may render peritoneal ECs more adhesive to monocytes. This, combined with the disrupted EC barrier in PD, promotes peritoneal inflammation and subsequent fibrosis.^15^, ^39^ Inhibition of EC glycolysis helps to reduce peritoneal damage, thereby improving peritoneal ultrafiltration in PD.

### 3.8 VEGF released from mesothelial cells induces EC glycolysis and dysfunction

**FIGURE 7:**
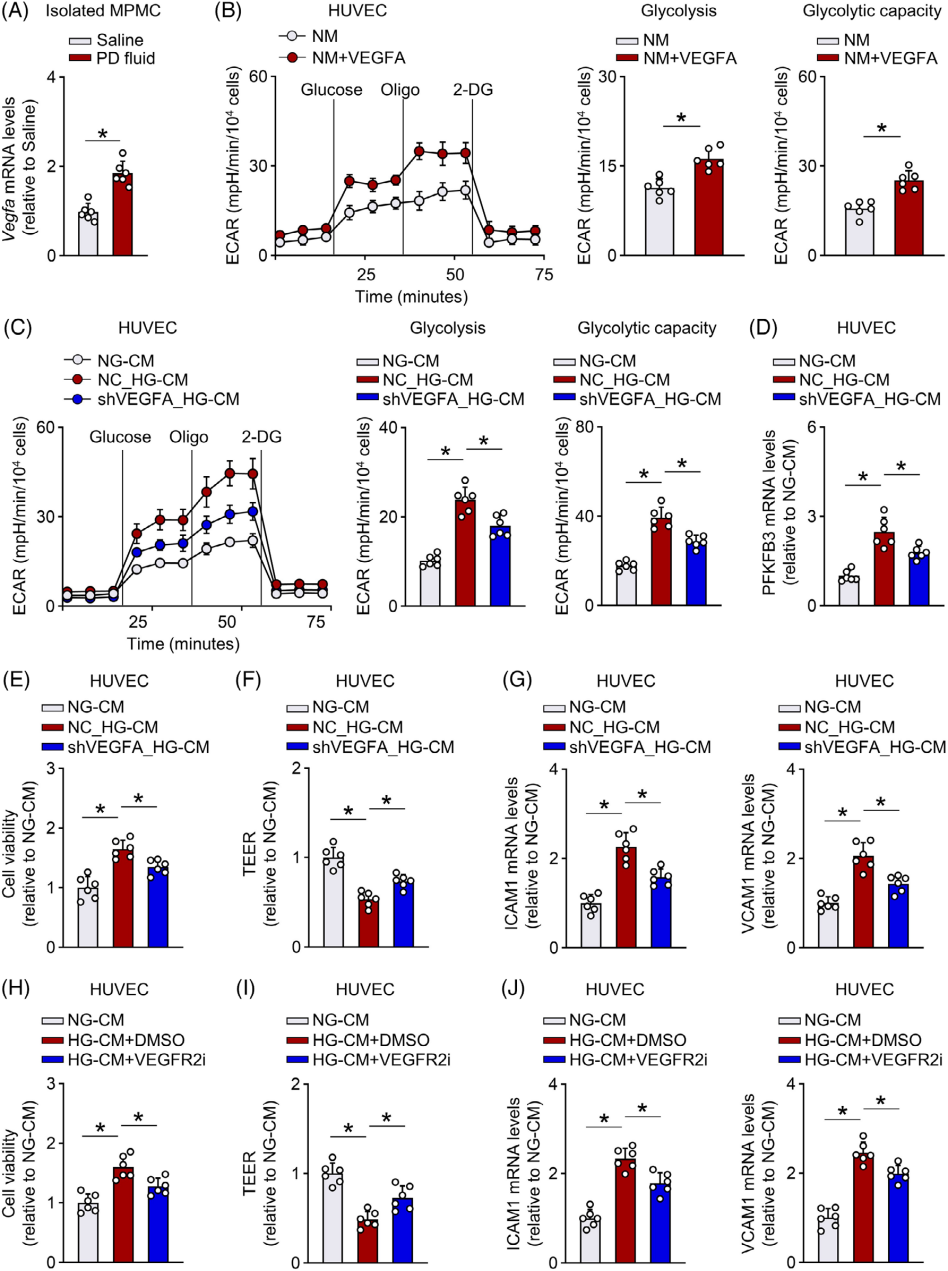
VEGF released from mesothelial cells induces endothelial cell (EC) glycolysis and dysfunction. (A) mRNA level of *Vegfa* in peritoneal mesothelial cells (MPMCs) isolated from saline and peritoneal dialysis (PD) fluid‐treated mice. *t*‐test; *p < .05. (B) VEGF administration increased ECAR in human umbilical venous ECs (HUVECs). *t*‐test; *p < .05. (C–G) Knockdown of VEGFA in human mesothelial cells (Met‐5A) reduced the HG‐CM‐induced increase in ECAR (C) and PFKFB3 expression (D). This reduced glycolysis was associated with reduced cell viability (E), improved TEER (F) and decreased expression of VCAM1 and ICAM1 (G). shVEGFA_HG‐CM, HG‐CM from shVEGFA‐transfected Met‐5A cells; NC_HG‐CM, HG‐CM from NC‐transfected Met‐5A cells. *t*‐test; *p < .05. (H–J) Pharmacological inhibition of VEGF receptor 2 (VEGFR2i, ZM 323881 HCl, 10 nM) in HUVECs reduced HG‐CM‐induced increase in cell viability (H), improved TEER (I), downregulated VCAM1 and ICAM1 expression (J). *t*‐test; *p < .05. Error bars, mean ± SD (*n* = 6 mice in each group in A; *n* = 6 cell samples in B–J).

We also sought to identify soluble factors in HG‐CM that promote EC glycolysis and dysfunction. The boiling of HG‐CM reversed its ability to increase cell viability, and downregulated the expression of key glycolysis‐related enzymes in HUVECs (Figure S9A,B). Thus, the active soluble factors in the HG‐CM are thermally unstable and likely to be cytokines released by mesothelial cells. VEGF is a potent proangiogenic factor implicated in peritoneal dysfunction.^9^ Here, transcripts of VEGF were elevated in mouse mesothelial cells from PD mice (Figure 7A). EC PFKFB3 deficiency did not significantly alter the VEGF expression in peritoneal mesothelial cells (Figure S9C). While VEGF administration in HUVECs upregulated PFKFB3 expression (Figure S9D) and increased ECAR (Figure 7B), supporting a role of VEGF in the enhancement of EC glycolysis.

Further in vitro experiments revealed that the VEGF concentration was approximately 2.7‐fold higher in HG‐CM compared to NG‐CM from human mesothelial cells (Figure S9E). Knockdown of VEGFA or Hif1α, a known VEGF transcriptional regulator,^21^, ^40^ in human mesothelial cells significantly reduced VEGF levels in HG‐CM (Figure S9F–J). HUVECs incubated with the VEGF‐depleted HG‐CM (vs. those treated with HG‐CM) had a reduction in glycolysis, as indicated by decreased ECAR and PFKFB3 expression (Figure 7C,D). This reduced glycolysis was closely associated with reduced cell proliferation, improved EC barrier tightness and decreased expression of VCAM1 and ICAM1 (Figure 7E–G). Consistently, pharmacological or genetical inhibition of VEGF receptor 2 in HUVECs also reduced the HG‐CM‐induced hyperglycolysis and partially improved the above‐mentioned EC function (Figure 7H–J and Figure S10), suggesting that VEGF generated by mesothelial cells contributes to EC hyperglycolysis and dysfunction.

We next tested our conclusion in primary HPECs. Similarly, the HG‐CM‐induced upregulation of glycolysis and EC dysfunction was significantly ameliorated by either knockdown of VEGF in human mesothelial cells (Figure S11A–E) or pharmacological blockade of VEGF receptor 2 in HPECs (Figure S11F–J). Together, VEGF released from mesothelial cells can increase EC glycolysis and induce EC dysfunction, supporting a role for hyperglycolysis in the development of peritoneal dysfunction during PD.

### 3.9 Upregulation of PFKFB3 in human peritoneal ECs is associated with microvascular alterations in ESKD patients

**FIGURE 8:**
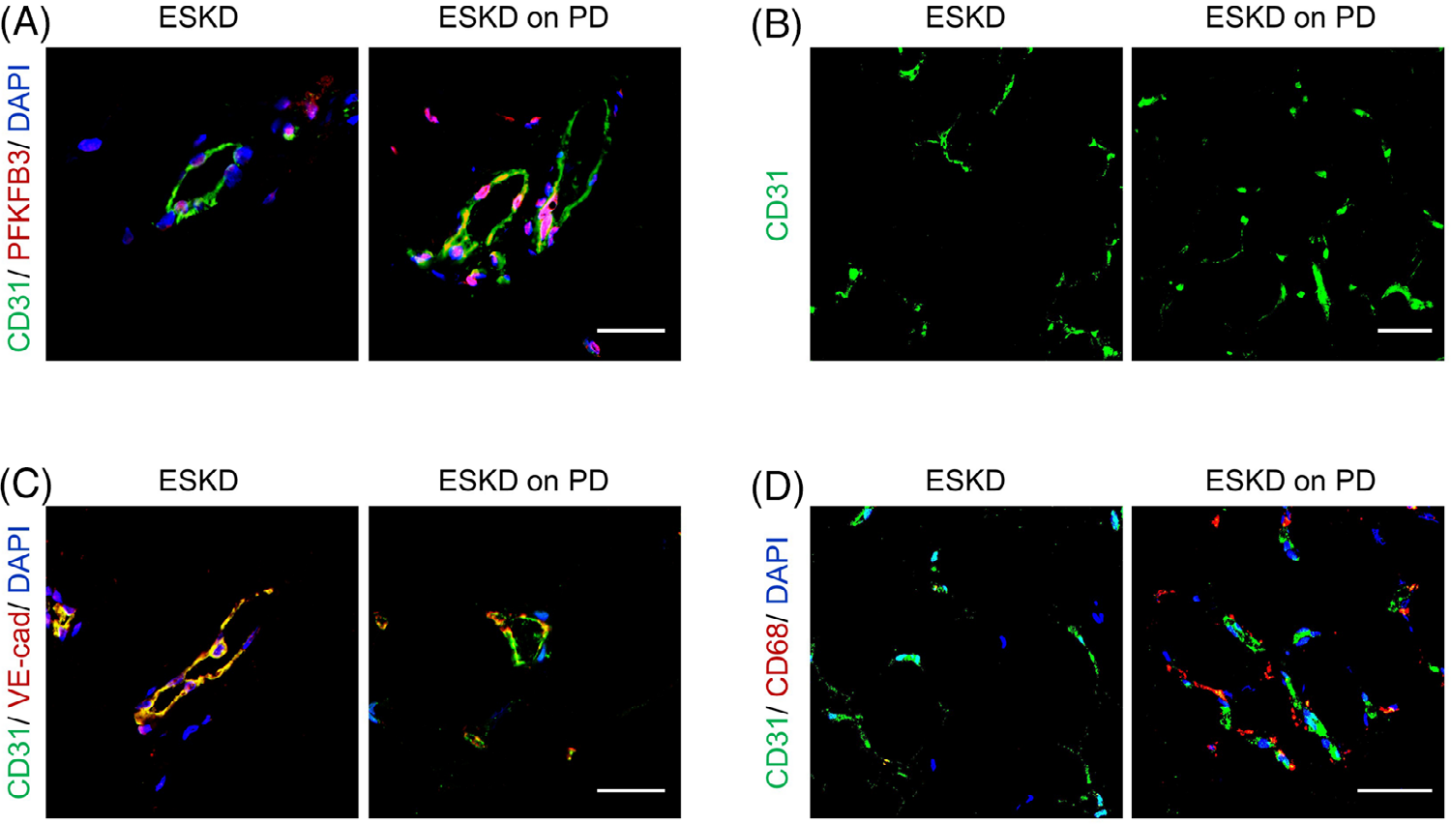
Upregulation of PFKFB3 in human peritoneal endothelial cells (ECs) is associated with microvascular alterations in end‐stage kidney disease (ESKD) patients. Human omentum was obtained from ESKD patients (*n* = 3 subjects) and ESKD patients on peritoneal dialysis (PD) (*n* = 3 subjects). (A) Immunostaining of omentum sections for CD31 (green) and PFKFB3 (red). Scale bar: 25 μm. (B) Immunostaining of omentum sections for CD31 (green). Scale bar: 50 μm. (C) Immunostaining of omentum sections for CD31 (green) and VE‐cadherin (red). Scale bar: 25 μm. (D) Immunostaining of omentum sections for CD31 (green) and CD68 (red). Scale bar: 50 μm.

Omentum tissue was obtained from ESKD patients on PD (*n* = 3 subjects) and from age‐ and serum creatinine‐matched ESKD patients (*n* = 3 subjects). The PFKFB3 expression was determined by immunostaining in human omentum tissue and was related to peritoneal microvessel alterations. The omentum from ESKD patients on PD (vs. ESKD patients) had upregulated expression of PFKFB3 in peritoneal ECs (Figure 8A), accompanied by increased microvessel density, decreased EC VE‐cadherin expression and increased macrophage infiltration in and around peritoneal vessels (Figure 8B–D).

## 4 DISCUSSION

**FIGURE 9:**
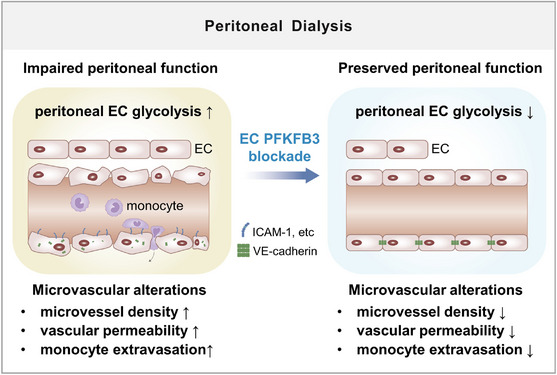
Schematic diagram summarizing the critical role of glycolysis in peritoneal endothelial cells (ECs) in facilitating peritoneal dysfunction during peritoneal dialysis (PD). In the setting of PD, peritoneal ECs have a hyperglycolytic metabolism that promotes endothelial proliferation, permeability and inflammation, culminating in peritoneal microvascular alterations and peritoneal dysfunction. Blocking glycolysis in peritoneal EC by genetic deletion or pharmacological inhibition of the glycolytic activator PFKFB3, reduces PD fluid‐induced increases in peritoneal capillary density, vascular permeability and monocyte extravasation, thereby protecting the peritoneum from the development of structural and functional damage. Mechanistically, EC PFKFB3 deficiency induces the protective effects in part by reducing proliferation, VE‐cadherin endocytosis and monocyte‐adhesion molecule expression in peritoneal ECs.

The major new finding of our study is a novel role of hyperglycolytic peritoneal ECs in the development of peritoneal dysfunction during PD (illustrated in Figure 9). We show that enhanced glycolysis in peritoneal ECs is associated with the development of PD fluid‐induced microvascular alterations and peritoneal membrane damage. Targeting this hyperglycolysis, by inhibiting endothelial PFKFB3, reduces PD fluid‐induced increases in peritoneal capillary density, vascular permeability and monocyte extravasation, thereby preserving the peritoneal structure and function in PD. Mechanistically, PFKFB3 blockade induces the protective effects in part by reducing proliferation, VE‐cadherin endocytosis and monocyte‐adhesion molecule expression in ECs. We demonstrate for the first time that metabolic reprogramming of ECs may be a critical mechanism underlying peritoneal dysfunction during PD. This glycolysis mechanism may also mediate peritoneal microvascular alterations in ESKD patients on PD.

Here, we characterize the metabolic profile of mouse peritoneal ECs in an experimental PD model by transcriptomic and metabolic analysis. This analysis reveals a strong, positive correlation between endothelial glycolysis and the peritoneal dysfunction during PD. ECs are highly glycolytic^18^ and generate 85% of their ATP via glycolysis even under resting conditions.^20^ We demonstrate that mouse peritoneal ECs further upregulate their glycolysis in response to PD fluid treatment. The functional importance of this hyperglycolysis is shown by endothelial PFKFB3 deficiency, which not only decreases glycolysis, but also lessens peritoneal fibrosis and dysfunction. These interesting observations identify peritoneal ECs with enhanced glycolysis as crucial participants in the development of peritoneal dysfunction during PD, which is unexpected given the modest volume of the EC population in the peritoneum.^27^ Hyperglycolysis in mesothelial cells has recently been linked to peritoneal fibrosis,^27^ but the contribution of the metabolic state in other cell types to peritoneal injury remains undefined. Here, we report the presence of a second metabolic mechanism within the peritoneum in PD, namely, hyperglycolysis in ECs.

We also find that soluble cytokines released from mesothelial cells appear to be an important upstream modulator of metabolism in peritoneal ECs. Mesothelial cells line the peritoneum and act as the primary cells that initiate the responses to PD fluid.^5^, ^27^ Here, using scRNA‐seq data of human peritoneal cells, we identify mesothelial cells as the most important cells that promote angiogenesis after long‐term PD, highlighting the mesothelial–endothelial crosstalk during PD. Indeed, exposure to cultured medium from high glucose‐treated mesothelial cells (HG‐CM), which mimics the in vivo peritoneal niche during PD, upregulates PFKFB3 expression and enhances glycolysis in both HUVECs and primary HPECs. In contrast, 1.5% glucose alone does not significantly alter glycolysis, suggesting that HG‐CM‐induced EC hyperglycolysis may be unrelated to the glucose in HG‐CM. We further demonstrate that active soluble factors in HG‐CM are thermally unstable. An important factor shown here to activate the EC glycolysis is VEGF, a well‐known cytokine involved in the development of peritoneal dysfunction during PD.^17^, ^41^, ^42^, ^43^, ^44^ Mesothelial cells produce VEGF even in a quiescent state.^45^ We demonstrate that mesothelial cell production of VEGF is amplified in PD mice. As VEGF is reported to drive PFKFB3 expression,^20^ we presume that VEGF mediates HG‐CM‐triggered hyperglycolysis in ECs. Indeed, the increase in EC glycolysis by HG‐CM is significantly inhibited by disrupting the VEGF regulator Hif1α^21^, ^40^ or VEGF signalling in mesothelial cells, or by interfering with VEGFR2 or PFKFB3 signalling in ECs. Interestingly, blockade of VEGF‐VEGFR2 signalling only partially reverses the HG‐CM‐induced EC dysfunction, suggesting that factors other than VEGF may also contribute to the enhanced glycolysis and the impaired EC dysfunction associated with PD.

There are several reasons that could account for why inhibition of PFKFB3‐mediated glycolysis in ECs protects the peritoneum from PD‐induced damage. First, endothelial PFKFB3 deficiency reduces EC proliferation and reverses the PD fluid‐induced peritoneal vascularization. ECs rely on glycolysis for high ATP‐demanding processes, including proliferation.^20^ Proliferating ECs use glucose carbons for nucleotide synthesis.^25^ Here, hyperglycolytic peritoneal ECs from PD mice express higher mRNA levels of enzymes involved in nucleotide synthesis. Stimulation of glycolysis by HG‐CM, a scenario similar to PD, enhances the transition from G1 to S phase and induces EC proliferation, migration and tube formation. We therefore suggest that peritoneal ECs are activated in PD. In support, PFKFB3 deficiency in ECs renders them more quiescent by lowering glycolysis and by disassociating the PFKFB3–CDK4 complex^32^ that controls the G1/S phase transition. This counteracts EC proliferative behaviours, reduces the peritoneal microvessel intensity, thereby decreasing solute transport^5^ and preserving peritoneal function.

Second, endothelial PFKFB3 deficiency tightens the EC barrier in the peritoneum by restoring endothelial VE‐cadherin expression, leading to reduced vascular permeability. VE‐cadherin is the principal cell–cell adhesion molecule of EC adheren junction.^34^ Disruption of VE‐cadherin is sufficient to disassemble blood vessels and increase the vascular permeability.^36^, ^46^ We demonstrate a downregulation of VE‐cadherin protein in hyperglycolytic peritoneal ECs from PD mice, which may be mechanistically mediated via a Clathrin‐dependent, ATP‐consuming^36^ endocytosis and increased oxidative stress.^26^ The reduced VE‐cadherin expression, functionally reflected by decreased electrical resistance in ECs, contributes to a leaky EC barrier. Thus, even if PD stimulates the proliferation of peritoneal ECs, the peritoneal vessels are still leaky. Importantly, endothelial PFKFB3 deficiency reverses this endocytosis process, elevates VE‐cadherin levels at EC surface, while increases the trans‐EC electrical resistance, all signs of barrier tightening and monolayer integrity.^9^, ^39^ Given that tightening of EC junctions contributes to vascular barrier integrity,^8^, ^35^ endothelial PFKFB3 inhibition may help to correct the vascular hyperpermeability and thereby restore solute transport in PD.

Third, endothelial PFKFB3 deficiency inhibits monocyte transendothelial migration, contributing to reduced peritoneal inflammation and fibrosis in PD. Hyperglycolytic peritoneal ECs from PD mice have elevated expression of monocyte adhesion molecules. This, combined with the disrupted EC barrier, would promote monocyte recruitment to the peritoneum, contributing to the development of peritoneal fibrosis.^10^, ^15^, ^47^ Indeed, macrophages in these PD mice display selective accumulation in and around peritoneal blood vessels. Notably, endothelial PFKFB3 deficiency lowers EC expression of monocyte adhesion molecules, reduces monocyte adhesion and transmigration across EC monolayers, and attenuates peritoneal macrophage infiltration. The effect of PFKFB3 knockout on reduced monocyte adhesion is related to reduced NF‐κB signalling, as PFKFB3 inhibition reduces levels of the glycolytic product lactate, which stimulates NF‐κB signalling in ECs.^25^ As inflammation drives fibrosis^15^, ^39^ that decreases the osmotic conductance of glucose and thus ultrafiltration in peritoneum,^13^ our findings suggest a role for EC PFKFB3 inhibition in the improved peritoneal function in PD.

Our studies have several important clinical implications. First, the endothelial PFKFB3 could become a therapeutic target to potentially prevent microvascular alterations and peritoneal dysfunction during PD. Most previous studies have focused on targeting angiogenic signals (such as VEGF) to reverse microvascular alterations in PD, which is limited by drug resistance, inadequate efficacy and systemic toxicity.^18^ Our results provide an alternative therapeutic option to induce vascular normalization in PD. As peritoneal ECs in the setting of PD display a unique phenotype with increased proliferation, permeability and inflammation, their quiescence by reducing glycolysis may counteract the active behaviour. Indeed, endothelial deficiency of the glycolytic activator PFKFB3, inhibited PD fluid‐induced increases in peritoneal capillary density, vascular permeability and monocyte extravasation, thereby protecting the peritoneum from the development of structural and functional damages. Also, drugs that block glycolysis may be of therapeutic benefit to patients undergoing PD. For example, the PFKFB3 antagonist PFK158, a 3PO analogue with improved inhibitory potency, might benefit patients with PD, as PFK158 is now in phase 1 safety study in patients with advanced solid malignancies.^48^

Our study also has limitations. First, we characterize the metabolic profile of peritoneal ECs isolated from a part of the mouse peritoneum, the mesentery. Although our findings from mesenteric ECs are corroborated in omentum ECs from ESKD patients on PD and in primary human parietal peritoneal ECs incubated with HG‐CM, the differences between different subsets of peritoneal ECs require further investigation. Second, we cannot rule out the adverse effect of EC PFKFB3 blockade on EC regeneration and repair. Here, EC PFKFB3 deficiency in vivo reduces glycolysis in mouse peritoneal ECs by approximately 33%. This reduction in EC glycolysis does not affect baseline peritoneal microvessel density, nor does it significantly alter EC nitric oxide generation, supporting the notion that PFKFB3 blockade does not lead to EC death, but rather induces proliferating ECs to enter a quiescent state.^20^, ^49^ The compensatory upregulation of other PFKFB isoforms after PFKFB3 deletion might account for the modest effects. Therefore, a dominant adverse effect of PFKFB3 blockade does not seem to play a role in our model. Third, we do not investigate the effect of factors other than VEGF, including epidermal growth factor and protein kinase Cα, which are activated in mesothelial cells during PD and contribute to peritoneal microvascular alterations.^50^, ^51^, ^52^ In addition, we do not measure glucose‐derived metabolites in peritoneal ECs from PD mice by using radio‐labelled glucose. However, we demonstrate increased glucose consumption and lactate excretion in the medium of peritoneal ECs from PD mice, and increased glycolysis measured by glycolytic rate assay in primary HPECs treated with HG‐CM. Finally, only male mice are used in our study. Therefore, gender differences in regulation of EC glycolysis and mechanisms of peritoneal dysfunction cannot be established, and extrapolation to females should be cautious.

In summary, our study demonstrates a hyperglycolytic metabolism in peritoneal ECs in PD mice, which is associated with the development of microvascular alterations and peritoneal membrane damage. Blocking glycolysis in peritoneal ECs, either genetically or pharmacologically, inhibits peritoneal vascularization, reduces vascular permeability and monocyte extravasation in the peritoneum, and thereby protects the peritoneum from the development of structural and functional damages. Maintaining the proper metabolic state of peritoneal ECs is a potential therapeutic goal to prevent or delay the peritoneal dysfunction in PD.

## AUTHOR CONTRIBUTIONS

W.C. conceived the project, analyzed the data, and wrote the manuscript. Z.S., W.S. and Z.Z. performed experiments, analyzed data, and wrote the manuscript. J.L., C.S., Y.Z., Z.H., Z.H., and H.Z. performed experiments, analyzed data. A.C. and Z.Z. analyzed data. W.C. and Z.S. verified the underlying data.

## CONFLICT OF INTEREST STATEMENT

The authors declare no potential conflicts of interest.

## ETHICAL APPROVAL

Studies using human omentum samples were approved by Ethics Committee in the Second Affiliated Hospital of Guangzhou Medical University and conducted in accordance with the Declaration of Helsinki (2021‐hs‐61).

## Supporting information

Supporting Information

Supporting Information Figure S1 PD fluid induces peritoneal fibrosis in mice.

Supporting Information Figure S2 Gene set enrichment analysis shows top 20 upregulated pathways in isolated MPECs from PD fluid‐treated mice compared to those from saline‐treated mice.

Supporting Information Figure S3 ScRNA‐seq data of human peritoneal cells demonstrate significant enhancement of mesothelial–endothelial crosstalk in patients on PD.

Supporting Information Figure S4 Soluble factors from mesothelial cells induce hyperglycolysis in ECs.

Supporting Information Figure S5 Genetic or pharmacological inhibition of EC PFKFB3 lessens peritoneal fibrosis in PD model.

Supporting Information Figure S6 Knockdown of PFKFB3 in ECs reverses the ability of HG‐CM to increase cell proliferation and improves EC barrier tightness.

Supporting Information Figure S7 Genetic inhibition of EC PFKFB3 attenuates endothelial inflammation.

Supporting Information Figure S8 Genetic inhibition of EC PFKFB3 attenuates peritoneal macrophage infiltration.

Supporting Information Figure S9 VEGF released from mesothelial cells upregulates glycolytic gene expression in ECs.

Supporting Information Figure S10 VEGF released from mesothelial cells induces EC glycolysis and dysfunction in HUVECs.

Supporting Information Figure S11 VEGF released from mesothelial cells induces ECs glycolysis and dysfunction in primary HPECs.

Supporting Information Figure S12 Uncropped immunoblots.

Supporting Information Figure S13 Histological sections in Figure 3.

## Data Availability

The sequencing data uploaded in the GSE database under accession number GSE230008. All remaining data used in this study are available in this article.
