# Supplementary material for: Hyperglycolysis in endothelial cells drives endothelial injury and microvascular alterations in peritoneal dialysis
Source: Clin Transl Med. 2023 Nov 30;13(12):e1498. doi: 10.1002/ctm2.1498 (PMC10689974; doi:10.1002/ctm2.1498)
Supplement: Supplementary file 1 — Supporting Information [file CTM2-13-e1498-s014.docx]

**Hyperglycolysis in endothelial cells drives endothelial injury and microvascular alterations in peritoneal dialysis**

Zekun Si, Wenyan Su, Zhuoyu Zhou, Jinjin Li, Cailing Su, Ying Zhang, Zuoyu Hu, Zhijie Huang, Hong Zhou, Ansheng Cong, Zhanmei Zhou, Wei Cao

**Supplementary material Table of Contents**

Supplemental Methods.

Supplemental Figure 1. PD fluid induces peritoneal fibrosis in mice

Supplemental Figure 2. Gene set enrichment analysis shows top 20 upregulated pathways in isolated MPECs from PD fluid-treated mice compared to those from saline-treated mice

Supplemental Figure 3. ScRNA-seq data of human peritoneal cells demonstrates significant enhancement of mesothelial-endothelial crosstalk in patients on PD

Supplemental Figure 4. Soluble factors from mesothelial cells induces hyperglycolysis in ECs

Supplemental Figure 5. Genetic or pharmacological inhibition of EC PFKFB3 lessens peritoneal fibrosis in PD model

Supplemental Figure 6. Knockdown of PFKFB3 in ECs reverses the ability of HG-CM to increase cell proliferation and improves EC barrier tightness

Supplemental Figure 7. Genetic inhibition of EC PFKFB3 attenuates endothelial inflammation

Supplemental Figure 8. Genetic inhibition of EC PFKFB3 attenuates peritoneal macrophage infiltration

Supplemental Figure 9. VEGF released from mesothelial cells upregulates glycolytic gene expression in ECs

Supplemental Figure 10. VEGF released from mesothelial cells induces EC glycolysis and dysfunction in HUVECs

Supplemental Figure 11. VEGF released from mesothelial cells induces ECs glycolysis and dysfunction in primary HPECs

Supplemental Figure 12. Uncropped immunoblots

Supplemental Figure 13. Histological sections in Figure 3

Supplemental Table 1. Patient characteristics

Supplemental Table 2. List of shRNAs/ siRNAs used to knock down the indicated targets

Supplemental Table 3. List of primers used for real-time PCR

**Supplemental Methods**

**Cell isolation and culture**

***Isolation of*** ***mouse peritoneal*** ***ECs and mesothelial cells***

For isolation of mouse peritoneal ECs,^1^ the mesentery along the small intestine was removed from mice under anesthesia with intraperitoneal (i.p.) injection of sodium pentobarbital (50 mg/kg body weight), and then digested with 0.02% collagenase. The digested solution was centrifuged and cell pellet was resuspended in PBS containing 0.5% BSA. Cells were then stained with antibodies anti-CD45-FITC (103107, Biolegend, CA, USA), anti-CD31-FITC (102407, Biolegend). ECs (CD31+ CD45-) were isolated by fluorescence-activated cell sorting (BD Biosciences, Franklin Lakes, NJ) (Figure S2A).

For isolation of peritoneal mesothelial cells,^2^ the mesentery along the small intestine was digested with 0.25% Trypsin-EDTA. Next, the digested solution was centrifuged and cell pellet was washed and resuspended in PBS containing 0.5% BSA. Mesothelial cells were isolated by fluorescence-activated cell sorting using anti-UPK3B antibody (1:1000, ab128568, Abcam, Cambridge, UK).^2^

Purified mouse peritoneal ECs or mesothelial cells were harvested and analyzed by Real-time PCR or western blot.

***Culture of mouse primary peritoneal ECs***

The mesentery along the small intestine was removed, digested and centrifuged as described above. The cell pellets were resuspended in the EC complete medium containing 5% FBS and 1% EC growth supplement (all from Gibco, Grand Island, NY, USA), and then plated onto a dish coated with gelatin (Sigma).^1^ Adherent ECs were grown to confluence in complete EC medium. Cells of passage 1 and 2 were used for experiments.

***Culture of human primary parietal peritoneal endothelial cells***

Primary human parietal peritoneal endothelial cells (HPECs, human; Procell, Wuhan, China) were cultured in cell media purchased from Procell. Passage 2 and 3 cells were used for experiments.

***Preparation of conditioned medium from human mesothelial cells***

Conditioned medium was prepared from human mesothelial cells that were exposed to high glucose (1.5% glucose, a concentration equivalent to 1.5% human PD fluid; HG-CM) or normal glucose (0.1% glucose; NG-CM), which mimics the *in vivo* peritoneal niche in PD or saline mice. The medium was aspirated, centrifuged, sterile filtered (0.22 μm filter). The glucose concentration in the HG-CM is not significantly different from that in 1.5% glucose solution (HG-CM *versus* 1.5% glucose: 81.39 ± 8.30 mM *versus* 82.23 ± 5.35 mM, *P*> 0.05; Figure S4E). Mesothelial cell-free medium with 0.1% glucose served as normal medium (NM). Mesothelial cell-free medium with 1.5% glucose served as high glucose medium (HM).

***Culture of human umbilical vein endothelial cells***

Human umbilical vein endothelial cells (HUVECs, human; American Type Culture Collection, VA, USA) were cultured in DMEM (5.5mM glucose) supplemented with 10% FBS and 1% penicillin-streptomycin (all from Gibco).

***Culture of human mesothelial cells***

Mesothelial cells (Met-5A, human; American Type Culture Collection) were cultured in Medium 199 supplemented with 10% FBS, 1% Insulin-Transferrin-Selenium, and 1% penicillin-streptomycin (all from Gibco).

**Gene transfection of cultured cells by shRNA or siRNA**

The shRNAs targeting VEGFR2, PFKFB3, HIF1A, VEGFA were synthesized and cloned into a lentiviral vector by Genechem. The lentivirus containing the scrambled nonsense shRNA was used as a negative control. The lentiviral shRNAs were then delivered to ECs as previously described.^3^ Briefly, HUVECs or Met-5A cells were seeded into 6-well plates to reach 60% confluence, and then infected with the shRNAs (50 MOI). After 24 hours, the cells were cultured in puromycin (3.0 g/ml) containing complete medium for a further 72 hours.

The siRNA targeted to VEGFR2 were used to knock down VEGFR2 in HPECs. Cells were seeded in 6-well plates and transfected with VEGFR2 siRNA (100nm, GeneChem) with lipofectamine RNAiMAX (Invitrogen, Carlsbad, CA, USA) according to the manufacturer’s instructions. In parallel, scrambled siRNA (100 nM) was used as negative controls.

The corresponding sequences are listed in Table S2.

**Single-cell Analysis**

A scRNA-seq data of human peritoneal cells in the GEO database (GSE130888) was analyzed to investigate the mesothelial-endothelial crosstalk in patients on PD. Cells were grouped and annotated by a marker gene set from a previous study.^4^ Gene set variation analysis (GSVA) was further used to explore the pathways involved in the differentially expressed genes (DEGs) in different groups or cell types. The Molecular Signatures Database (MSigDB) v.5.2 (http://software.broadinstitute.org gseamsigdb/indexjsp) was used to get gene sets for boarchallmarkers.

**Measurement of F-2,6-P2 levels**

F-2,6-P2 levels in cell homogenates were measured in isolated mouse peritoneal ECs in the presence of 0.5 mM pyrophosphate and 1 mM fructose 6-phosphate.^5^

**Peritoneal permeability test**

Peritoneal function was measured by modified peritoneal equilibration tests.^6^ Before mice were sacrificed, 3 ml of 4.25% PD fluid was intraperitoneally injected. Dialysate and blood samples were collected at 0 hour and 2 hours of dwell time. The concentrations of glucose and urea nitrogen in the dialysate and plasma were measured using an automatic biochemical analyzer (Beckman, CA, USA). The peritoneal permeability to glucose was expressed as the ratio of glucose concentration in 2-hour peritoneal dialysate to 0-hour peritoneal dialysate (D/D_0_). The peritoneal permeability to blood urea nitrogen was expressed as the ratio of urea nitrogen concentration in dialysate to plasma at 2 hours (D/P). The net ultrafiltration was calculated by removal of 3 ml from the 2-hour dialysate volume.

**Peritoneum histological procedures**

Under anesthesia with sodium pentobarbital (50 mg/kg body weight, i.p.), mice were transcardially perfused, first with PBS and then with 4% paraformaldehyde.^7^ The parietal peritoneal tissues were isolated, embedded in paraffin, and sectioned at a thickness of 4 μm. Peritoneal fibrosis was assessed on paraffin sections processed for Masson’s trichrome staining. The degree of fibrosis was calculated by the thickness of fibrotic (blue-stained) area in each section.

**Immunostaining**

Immunostaining was performed as previously described.^7^ Briefly, paraffin sections (4 µm) or 4% paraformaldehyde-fixed cells were labeled with primary antibodies at 4°C for 1 day and then reacted with respective secondary antibodies (Thermo Fisher Scientific, MA, USA) at 37°C for 1 hour. Sections or paraformaldehyde-fixed cells were visualized under a Leica confocal microscope (Leica TCS SP2 AOBS, IL, USA). Primary antibodies used were listed as follows: anti-CD31 (1:50, AF3628, R&D Systems, MN, USA), α-SMA (1:50, BM0002, Boster), anti-CD45 (1:50, ab282747), anti-VE-cadherin (1:50, ab205336), anti-ICAM1 (1:50, ab171123), anti-UPK3B (1:50, ab237778) (all from Abcam), anti-F4/80 (1:50, 70076, Cell Signaling Technology, MA, USA) antibody.

**Liquid chromatography-mass spectrometry (LC/MS)**

Intracellular metabolite levels were determined using Liquid chromatography-mass spectrometry.^8^ Briefly, metabolites in the 200 μL cell sample were extracted with 600 μL methanol. Proteins in the extracted fluid were then pelleted by centrifugation at 14,000rpm for 15 minutes at 4°C. The supernatant was dried under nitrogen stream, resuspended with 100 μL of 50% methanol, and then centrifuged at 14000rpm for 15 minutes at 4°C.The final supernatant was transferred to LC/MS vials.

Metabolite analysis was performed using an ultra-high-performance liquid chromatography coupled with Q-Exactive hybrid Orbitrap mass spectrometry (UHPLC-QE-Orbitrap-MS) (Thermo Fisher Scientific). Briefly, 5 μl of sample was injected on a ACQUITY UPLC BEHC18 column (WATERS). The flow was kept constant at 300 μl/min at the column was placed at 40°C throughout the analysis. The MS (Q-Exactive ORBITRAP, Thermo) operated in full (negative) scan using a spray voltage of 3.5 kV, capillary temperature of 320°C, sheath gas at 35.0, auxiliary gas at 10.0. For Full scan, AGC target was set at 3e6 using a resolution of 70.000.

**Real-time energy metabolism assessment**

Cells were seeded onto Seahorse XF96 polystyrene tissue culture plates (Seahorse Bioscience) at the density of 1 ×10^4^ cells/well, and incubated overnight at 37°C.^2^ Glycolytic activity (indicated by ECAR) was studied using the Seahorse Bioscience XFe96 Extracellular Flux Analyzer for the XF Glycolysis Stress Test according to the manufacturer’s instructions (Agilent Technologies, CA, USA).

Glycolytic rate was performed using the glycolytic rate assay kit from Agilent Technologies and was analyzed by sequentially adding pharmacological inhibitors of oxidative phosphorylation, namely rotenone/antimycin A (0.5 μM) and 2-DG (50 mM).^9^

**CCK8 assay**

Cells were seeded into 96-well plates at a density of 3 × 10^3^ cells per well. Cell viability was measured at the indicated time points, using a Cell Counting Kit (CCK8, Dojindo, Japan) according to the manufacturer’s instructions. Absorbance at 450 nm was measured using a multifunctional microplate reader (Bio-Rad Laboratories, Hercules, CA).

**EdU incorporation assay**

ECs were stained with the Click-iT Plus EdU Alexa Fluor 488 Imaging Kit according to the manufacturer’s instructions (Invitrogen). Stained specimens were viewed under a Leica TCS confocal microscope equipped with a digital camera. Quantification of EdU-positive cells was performed using Image J software (NIH, USA).

**Cell-cycle analysis using flow cytometry**

ECs were washed with cold PBS and then stained with the FxCycle PI/RNase Staining Solution (Invitrogen) according to the manufacturer’s instructions. Cell cycle distribution was evaluated using a FACSCanto II Flow cytometry (Becton Dickinson, CA, USA). The percentage of cells in each phase of the cell cycle was determined using the ModFit LT5.0 software (Verity Software House, USA).

***In vitro* tube formation assay**

Tube formation of ECs was determined as previously described.^10^ Briefly, HUVECs (4 × 10^4^ cells) were suspended and seeded on a 24-well plate (300 μl/well) coated with matrigel matrix (Becton Dickinson). After 6 hours of incubation at 37 °C, tube formation of ECs was observed under a Leica TCS confocal microscope equipped with a digital camera. Five random fields per sample were photographed at 200× magnification.

**Scratch wound migration assay**

A scratch wound was applied on a confluent cell monolayer using a 200 µl tip. After scratch wounding and photography at time zero (T0), the cultures were stimulated by indicated treatments in the presence of Mitomycin C (Sigma-Aldrich) to block proliferation, and photographed again at 48 hours (T48). Migration distance (gap area at T0 minus gap area at T48) was measured using the NIH Image J software.

***In vivo* vascular permeability assay**

Vascular permeability of the mouse peritoneum was measured using Evans blue dye with minor modifications.^11^ Briefly, 2 hours after tail vein injection of Evans blue (0.5% dissolved in sterile PBS), mice received an intraperitoneal injection of cold PBS (2ml). The peritoneal fluid was then collected and centrifuged at 2500 rpm for 10 minutes. Evans blue in the peritoneal fluid was quantified by spectrophotometric analysis at 620 nm. An equal volume of PBS was used as a blank control.

**VE-cadherin endocytosis assay**

To visualize internalized VE-cadherin,^12^ ECs were incubated with anti-VE-cadherin antibody (sc-52751, Santa Cruz Biotechnology, Dallas, TX, USA) in the presence of 150 µM of the lysosomal inhibitor chloroquine (Sigma-Aldrich) at 37°C for 4 hours. To remove cell surface-bound antibody, the HUVECs were then exposed to mild acid buffer (2 mM PBS-glycine, pH 2.0, for 10 minutes) and washed twice with PBS. Next, the cells were fixed with 4% PFA, incubated with an Alex fluor 488-labeled secondary antibody (Thermo Fisher Scientific) at 37°C for 1 hour, and detected with a Leica confocal microscope.

***In vitro* endothelial permeability assay**

Paracellular permeability was assessed in a transwell system (Corning, Union City, CA, USA) by measuring the flux of FITC-dextran across the EC monolayer.^10^ Briefly, the EC monolayer was stimulated with indicated treatments in the upper chamber. Then, 100 µl of PBS containing FITC-dextran (1 mg/ml, Thermo Fisher Scientific) was added to the upper chamber and 500 µl of PBS was added to the lower chamber. After 1 hour of incubation, the concentration of FITC-dextran transferred to the lower chamber was determined using a multimode microplate reader (Agilent) with excitation and emission wavelengths of 492 and 520 nm, respectively.

**Transendothelial electrical resistance (TEER) measurement**

TEER, which reflects the paracellular permeability of endothelial monolayers, was measured using established protocols.^13^ Briefly, ECs were grown to confluence on gelatin-coated gold electrodes. After recording the resistance value at time zero, cells were stimulated with indicated treatments and the TEER was measured again (ECIS system, Applied Biophysics, Troy, NY, USA). Data were presented as changes in TEER after treatments.

**Monocyte adhesion assay**

Monocyte adhesion assay was performed according to an established protocol.^14^ Briefly, THP-1 cells labeled with Calcein-AM (Thermo Fisher Scientific) were seeded on the EC monolayer for 30 minutes. Adherent THP-1 cells were visualized by fluorescence microscopy and quantified manually (a minimum of 5 fields/well were quantified).

**Assessment of nitric oxide production**

Cellular nitric oxide (NO) production was measured using the Total Nitric Oxide Assay Kit (Beyotime, Shanghai, China) accordingly to manufactory’s instruction. The optical densities at 540 nm wavelength were recorded using a DTX 880 Multimode Detector and the concentrations of NO were calculated according to the standard curve.^15^

**Intracellular ROS measurement**

The intracellular ROS levels were determined using a ROS Assay Kit (Beyotime) accordingly to manufactory’s instruction. Briefly, the cells were treated under different conditions and incubated with 2′,7′-Dichlorodihydro-fluorescein diacetate (DCFH-DA, with excitation and emission spectrum peak wavelengths of 488 nm/525 nm) for 30 min at 37 ℃. Then, cells were analyzed on a Synergy™ HTX Multi-Mode Microplate Reader (Agilent).

**Measurement of ATP**

Intracellular ATP content was determined using the Enhanced ATP Assay Kit (Beyotime) following the manufacturer’s instructions.

**Glucose consumption and lactate secretion**

Medium from mouse peritoneal ECs were collected after 48 hours. Glucose concentration in the medium was determined using an automatic biochemical analyzer (AU5831; Backman Coulter, CA). Lactate levels in the medium was determined using a L-Lactate Assay Kit (Cayman, MI, US).

**Western blot analysis**

Lysates of tissues or cells were prepared, and western blots were performed^16^ with the following primary antibodies: anti-collagen I (1:500, BA0325, Boster, Wuhan, China), anti-fibronectin (1:1000, 15613-1-AP, ProteinTech, IL, USA), anti-PFKFB3 (1:1000, ab181861), anti-VEGFR2 (1:1000, ab134191), anti-UPK3B (1:1000, ab237778) (all from Abcam), anti-CDK4 (1:1000, AF5254, R&D System), anti-VE-cadherin (1:250, sc-52751, Santa Cruz Biotechnology), anti-Clathrin (1:1000, 4796), p65 (1:1000, [8242](https://www.cellsignal.cn/products/primary-antibodies/nf-kb-p65-d14e12-xp-rabbit-mab/8242?site-search-type=Products&N=4294956287&Ntt=p65&fromPage=plp)), p-p65 (1:1000, 3033) (all from Cell Signaling Technology) antibody followed by specific secondary antibodies (LICOR, NE, USA). β-actin antibody (1:1000, GTX109639, GeneTex, San Antonio, TX, USA) was used as loading controls. Band densitometry was performed using ImageJ software (NIH, USA).

**Co-immunoprecipitation assay**

Immunoprecipitations were performed as previously described.^17^ Briefly, cell lysates were immunoprecipitated by antibody against PFKFB3 (1:100, Abcam) or Clathrin (1:100, Santa Cruz Biotechnology). Immune complexes were washed five times with lysis buffer and then analyzed by western blot.

**Real-time PCR**

Total RNA was isolated from cells or tissues using TRIzol reagent (Invitrogen), or RNeasy Plus Micro Kit (Qiagen, Hilden, Germany). Real-time PCR was performed using TaKaRa SYBR® Premix Ex Taq^TM^ II kit (Takara Biotechnology, Shiga, Japan), with β-actin serving as the internal control. The primers used were listed in Table S3.

**Enzyme-linked immunosorbent assay (ELISA)**

VEGFA concentrations in supernatants of cultured cells were detected using an ELISA kit (Raybiotech, Norcross, GA, USA) according to manufacturer’s instruction.

**REFERENCES**

1. Snead MD, Papapetropoulos A, Carrier GO, Catravas JD. Isolation and culture of endothelial cells from the mesenteric vascular bed. *Methods in Cell Science.* 1995;17(4)**:** 257-262.

2. Si M, Wang Q, Li Y, et al. Inhibition of hyperglycolysis in mesothelial cells prevents peritoneal fibrosis. *Sci Transl Med.* 2019;11(495).

3. Yao J, Wu XY, Yu Q, et al. The requirement of phosphoenolpyruvate carboxykinase 1 for angiogenesis in vitro and in vivo. *Sci Adv.* 2022;8(21)**:** n6928.

4. Su W, Hu Z, Zhong X, et al. Restoration of CPT1A-mediated fatty acid oxidation in mesothelial cells protects against peritoneal fibrosis. *Theranostics.* 2023;13(13)**:** 4482-4496.

5. Cao Y, Zhang X, Wang L, et al. PFKFB3-mediated endothelial glycolysis promotes pulmonary hypertension. *Proc Natl Acad Sci U S A.* 2019;116(27)**:** 13394-13403.

6. Shi Y, Tao M, Wang Y, et al. Genetic or pharmacologic blockade of enhancer of zeste homolog 2 inhibits the progression of peritoneal fibrosis. *J Pathol.* 2020;250(1)**:** 79-94.

7. Cao W, Wu L, Zhang X, et al. Sympathetic Overactivity in CKD Disrupts Buffering of Neurotransmission by Endothelium-Derived Hyperpolarizing Factor and Enhances Vasoconstriction. *J Am Soc Nephrol.* 2020;31(10)**:** 2312-2325.

8. Cantelmo AR, Conradi LC, Brajic A, et al. Inhibition of the Glycolytic Activator PFKFB3 in Endothelium Induces Tumor Vessel Normalization, Impairs Metastasis, and Improves Chemotherapy. *Cancer Cell.* 2016;30(6)**:** 968-985.

9. Zhang Y, Shan P, Srivastava A, Li Z, Lee PJ. Endothelial Stanniocalcin 1 Maintains Mitochondrial Bioenergetics and Prevents Oxidant-Induced Lung Injury via Toll-Like Receptor 4. *Antioxid Redox Signal.* 2019;30(15)**:** 1775-1796.

10. Yang K, Fan M, Wang X, et al. Lactate induces vascular permeability via disruption of VE-cadherin in endothelial cells during sepsis. *Sci Adv.* 2022;8(17)**:** m8965.

11. Yang X, Yan H, Jiang N, et al. IL-6 trans-signaling drives a STAT3-dependent pathway that leads to structural alterations of the peritoneal membrane. *Am J Physiol Renal Physiol.* 2020;318(2)**:** F338-F353.

12. Gong H, Rehman J, Tang H, et al. HIF2alpha signaling inhibits adherens junctional disruption in acute lung injury. *J Clin Invest.* 2015;125(2)**:** 652-664.

13. Tiruppathi C, Malik AB, Del VP, Keese CR, Giaever I. Electrical method for detection of endothelial cell shape change in real time: assessment of endothelial barrier function. *Proc Natl Acad Sci U S A.* 1992;89(17)**:** 7919-7923.

14. Yang Q, Xu J, Ma Q, et al. Disruption of endothelial Pfkfb3 ameliorates diet-induced murine insulin resistance. *J Endocrinol.* 2021;250(3)**:** 93-104.

15. Jing C, Zhang G, Liu Z, et al. Peroxidasin promotes diabetic vascular endothelial dysfunction induced by advanced glycation end products via NOX2/HOCl/Akt/eNOS pathway. *Redox Biol.* 2021;45(102031.

16. Cao W, Li A, Wang L, et al. A Salt-Induced Reno-Cerebral Reflex Activates Renin-Angiotensin Systems and Promotes CKD Progression. *J Am Soc Nephrol.* 2015;26(7)**:** 1619-1633.

17. Su H, Yang Z, Zhang Y, et al. Neural and central mechanisms of kidney fibrosis after relief of ureteral obstruction. *iScience.* 2023;26(4)**:** 106338.

**Figure legends**

**Supplemental Figure 1. PD fluid induces peritoneal fibrosis in mice**

(A) Fibrosis in the mesentery presented by Masson’s trichrome staining (Scale bar, 50 μm). One-way ANOVA followed by Bonferroni test; *, *P*<0.05. (B) mRNA levels of *Fn1* and *Col1a1* in the mesentery. One-way ANOVA followed by Bonferroni test; *, *P*<0.05. (C) The peritoneal permeability of glucose (D/D_0_ glucose) examined by modified peritoneal equilibration test. One-way ANOVA followed by Bonferroni test; *, *P*<0.05. (D) Immunostaining of the mesentery for CD31 and UPK3B (marker of mesothelial cell). Scale bar, 100 μm. (E) Expression of UPK3B in homogenates of mesentery. Error bars, mean ± SD (n=6 mice in each group).

**Supplemental Figure 2.** **Gene set enrichment analysis shows top 20 upregulated pathways in isolated MPECs from PD fluid-treated mice compared to those from saline-treated mice**

(A) The schematic diagram of sorting MPECs by fluorescence-activated cell sorting from mouse peritoneum. (B) The top 20 KEGG pathways ranked by normalized enrichment scores identified by gene set enrichment analysis of MPECs from PD fluid-treated mice compared to saline-treated mice. (C) Heatmap analysis showing transcript levels of nucleotide metabolism, amino acid metabolism and carbohydrate metabolism-related genes in RNA sequencing data of isolated MPECs. (D) mRNA levels of genes involved in glycolysis in isolated MPECs. *t* test, **P*<0.05. (E) Protein levels of genes involved in glycolysis in isolated MPECs. *t* test, **P*<0.05. (F) Glucose consumption and lactate excretion in the medium of MPECs from PD fluid- and saline-treated mice. *t* test, **P*<0.05. Error bars, mean ± SD (n=6 mice in each group).

**Supplemental Figure 3. ScRNA-seq data of human peritoneal cells demonstrates significant enhancement of mesothelial-endothelial crosstalk in patients on PD**

(A) t-Distributed Stochastic Neighbor Embedding (tSNE) shows single-cell populations from the normal peritoneum (normal, n = 3) and from effluent of patients on PD (ST-PD, n = 6; LT-PD, n = 4) separately using scRNA-seq data GSE130888. (B) GSVA analysis demonstrating enrichment of gene sets involved in angiogenesis in the scRNA-seq data stratified by cell type. (C) GSVA analysis demonstrating enrichment of gene sets involved in angiogenesis in the mesothelial population stratified by patient group.

**Supplemental Figure 4. Soluble factors from mesothelial cells induces hyperglycolysis in ECs**

(A) Cell viability measured by CCK-8 assay in HUVECs treated with different concentrations of glucose (1.5-4.25%, concentrations equivalent to 1.5-4.25% human PD fluids). One-way ANOVA followed by Bonferroni test; *, *P*<0.05. (B) Cell viability measured by CCK-8 assay in HUVECs treated with different concentrations of mannitol (1.5-4.25%). One-way ANOVA followed by Bonferroni test; *, *P*<0.05. (C) mRNA levels of key glycolytic genes in HUVECs treated with 1.5% glucose. *t* test. (D) mRNA levels of key glycolytic genes in HUVECs treated with 1.5% mannitol. *t* test. (E) The glucose concentration in 1.5% glucose and HG-CM. *t* test; ns, no significance. (F) Cell viability measured by CCK-8 assay in HUVECs treated with NM, NG-CM, or HG-CM with glucose concentrations at 1.5-4.25%. One-way ANOVA followed by Bonferroni test; *, *P*<0.05. (G) mRNA levels of key glycolytic genes in HUVECs treated with NM, NG-CM or HG-CM (1.5% glucose). One-way ANOVA followed by Bonferroni test; *, *P*<0.05. (H) ECAR determined by Glycolysis Stress assay in HUVECs. One-way ANOVA followed by Bonferroni test; *, *P*<0.05. (I) Knockdown of PFKFB3 in HUVECs markedly reduced the elevation of ECAR stimulated by HG-CM. *t* test; *, *P*<0.05. Error bars, mean ± SD (n=6 cell samples in each group).

**Supplemental Figure 5. Genetic or pharmacological inhibition of EC PFKFB3 lessens peritoneal fibrosis in PD model**

(A) PD fluid-induced upregulation of fibronectin (FN) and collagen (Co) I in peritoneal tissue was markedly attenuated in *Pfkfb3*^ΔEC^ mice. One-way ANOVA followed by Bonferroni test; *, *P*<0.05. (B) Pharmacological inhibition of PFKFB3 by 3PO treatment starting from day 1 of PD fluid administration, reduced PD fluid-induced upregulation of FN and Co I in mouse peritoneal tissue. One-way ANOVA followed by Bonferroni test; *, *P*<0.05. (C-E) Treatment with 3PO, initiated after 3 weeks of PD fluid administration, attenuated PD fluid-induced upregulation of *Fn1* and *Col1a1* mRNA (C) and improved ultrafiltration volume (D), D/D_0_ glucose and D/P of BUN (E) at week 6. One-way ANOVA followed by Bonferroni test; *, *P*<0.05. Error bars, mean ± SD (n=6 mice in each group).

**Supplemental Figure 6. Knockdown of PFKFB3 in ECs reverses the ability of HG-CM to increase cell proliferation and improves EC barrier tightness**

(A) GSEA plots demonstrating ES for migration pathway in RNA sequencing data of isolated MPECs. (B) F2,6-P2 levels in MPECs from mice treated with 3PO or DMSO. *t* test; *, *P*<0.05. (C) Immunofluorescence staining for CD31 (green) in the mesentery from PD mice and PD mice treated with 3PO. Quantitative data showing the vascular density/ field. Scale bar, 50 μm. One-way ANOVA followed by Bonferroni test; *, *P*<0.05. (D) Primary HPECs transfected with siPFKFB3 or negative control (NC) were incubated with HG-CM for 24 hours. EdU incorporation images and percentage of EdU-positive cells were shown. Scale bar, 100 μm. One-way ANOVA followed by Bonferroni test; *, *P*<0.05. (E) CCK-8 assay showing cell viability in primary HPECs. One-way ANOVA followed by Bonferroni test; *, *P*<0.05. (F and G) Treatment of HUVECs with HG-CM (*versus* NM) for 24 hours increased the binding of PFKFB3 to CDK4 (F) and upregulated the CDK4 expression (G). Knockdown of PFKFB3 in HUVECs inhibited the upregulation of CDK4 stimulated by HG-CM (G). One-way ANOVA followed by Bonferroni test; *, *P*<0.05. (H) Knockdown of PFKFB3 did not significantly affect nitric oxide (NO) generation in HUVECs. *t* test. (I) Primary HPECs transfected with siPFKFB3 or NC were incubated with HG-CM for 24 hours. VE-cad protein levels were shown. One-way ANOVA followed by Bonferroni test; *, *P*<0.05. (J) *In vitro* permeability assay using FITC-dextran demonstrating permeability of primary HPECs. One-way ANOVA followed by Bonferroni test; *, *P*<0.05. (K) Transendothelial electrical resistance (TEER) in primary HPECs. One-way ANOVA followed by Bonferroni test; *, *P*<0.05. (L) ATP levels in shPFKFB3- or NC-transfected HUVECs after incubation with HG-CM. One-way ANOVA followed by Bonferroni test; *, *P*<0.05. (M) ROS levels in HUVECs. One-way ANOVA followed by Bonferroni test; *, *P*<0.05. (N) TEER in HUVECs incubated with 1.5% glucose or mannitol. *t* test; *, *P*<0.05. Error bars, mean ± SD (n=6 cell samples in each group).

**Supplemental Figure 7. Genetic inhibition of EC PFKFB3 attenuates endothelial inflammation**

(A) Protein levels of ICAM1 and VCAM1 in MPECs from PD fluid- and saline-treated mice. One-way ANOVA followed by Bonferroni test; *, *P*<0.05. (B) mRNA levels of ICAM1 and VCAM1 in siPFKFB3- or NC-transfected primary HPECs after incubation with HG-CM. (C) Phosphorylation of NF-κB subunit p65 in shPFKFB3- or NC-transfected HUVECs after incubation with HG-CM. One-way ANOVA followed by Bonferroni test; *, *P*<0.05. (D) Protein levels of ICAM1 and VCAM1 in shPFKFB3- or NC-transfected HUVECs after incubation with HG-CM. One-way ANOVA followed by Bonferroni test; *, *P*<0.05. (E) VCAM1 mRNA level in HUVECs treated with 1.5% glucose or mannitol. *t* test. One-way ANOVA followed by Bonferroni test; *, *P*<0.05. Error bars, mean ± SD (n=6 cell samples in each group).

**Supplemental Figure 8. Genetic inhibition of EC PFKFB3 attenuates peritoneal macrophage infiltration**

(A) UMAP plots demonstrating the expression of CD14, HLA-DRB1, CD68, and ITGAM (CD11b) in human macrophage population selected from Figure S3A. The color gradient is based on the normalized gene expression. (B) UMAP plots demonstrating the expression of CD14, CD36, Adgre1 (F4/80), and Itgam (CD11b) in mouse macrophage population selected from scRNA-seq data of mouse peritoneal cells (GSE 139999). The color gradient is based on the normalized gene expression. (C) Immunostaining of mesentery sections for UPK3B (green) and F4/80 (red). Scale bar, 100 μm. (D) Immunostaining of parietal peritoneum sections for α-SMA (green) and F4/80 (red). Scale bar, 100 μm. Error bars, mean ± SD (n=6 mice in each group).

**Supplemental Figure 9. VEGF released from mesothelial cells upregulates glycolytic gene expression in ECs**

(A) Cell viability measured by CCK-8 assay in HUVECs treated with NM, HG-CM or boiled HG-CM. One-way ANOVA followed by Bonferroni test; *, *P*<0.05. (B) mRNA levels of key glycolytic genes in HUVECs treated with NM, HG-CM or boiled HG-CM. One-way ANOVA followed by Bonferroni test; *, *P*<0.05. (C) *Vegfa* mRNA levels in MPECs from *Pfkfb3*^WT^ and *Pfkfb3*^ΔEC^ mice. *t* test; ns, no significance. (D) PFKFB3 mRNA levels in HUVECs treated with or without VEGFA. *t* test*, *P*<0.05. (E) VEGFA content in cultured medium of human mesothelial line Met-5A cells treated with normal glucose (NG, 0.1%) or high glucose (HG, 1.5%). *t* test; *, *P*<0.05. (F) HIF1A mRNA levels in shHIF1A- or NC-transfected Met-5A cells. *t* test; *, *P*<0.05. (G) VEGFA content in cultured medium of shHIF1A- or NC-transfected Met-5A cells. *t* test; *, *P*<0.05. (H) Protein levels of PFKFB3 in HUVECs after incubation with HG-CM from shHIF1A- (shHIF1A_HG-CM) or NC- (NC_HG-CM) transfected Met-5A cells. One-way ANOVA followed by Bonferroni test; *, *P*<0.05. (I) VEGFA mRNA levels in shVEGFA- or NC-transfected Met-5A cells. *t* test; *, *P*<0.05. (J) VEGFA content in cultured medium of shVEGFA- or NC-transfected Met-5A cells. *t* test; *, *P*<0.05. Error bars, mean ± SD (n=6 cell samples in each group).

**Supplemental Figure 10. VEGF released from mesothelial cells induces EC glycolysis and dysfunction in HUVECs**

(A) Incubation of HUVECs with a VEGF receptor 2 inhibitor (VEGFR2i, ZM 323881 HCl, 10nM) reduced the upregulation of ECAR stimulated by HG-CM. One-way ANOVA followed by Bonferroni test; *, *P*<0.05. (B) VEGFR2i administration in HUVECs inhibited the upregulation of PFKFB3 mRNA level stimulated by HG-CM. One-way ANOVA followed by Bonferroni test; *, *P*<0.05. (C) Knockdown of VEGFR2 by shVEGFR2 in HUVECs inhibited the upregulation of ECAR stimulated by HG-CM. One-way ANOVA followed by Bonferroni test; *, *P*<0.05. (D) Knockdown of VEGFR2 in HUVECs reduced HG-CM-induced upregulation of PFKFB3. One-way ANOVA followed by Bonferroni test; *, *P*<0.05. (E-G) Knockdown of VEGFR2 in HUVECs reduced HG-CM-induced increase in cell viability (E), improved TEER (F), and decreased mRNA levels of ICAM1 and VCAM1 (G). One-way ANOVA followed by Bonferroni test; *, *P*<0.05. Error bars, mean ± SD (n=6 cell samples in each group).

**Supplemental Figure 11. VEGF released from mesothelial cells induces ECs glycolysis and dysfunction in primary HPECs**

(A-E) Knockdown of VEGFA in human mesothelial cells reduced the HG-CM-induced increase in ECAR (A) and PFKFB3 expression (B) in HPECs. This reduced glycolysis was associated with reduced cell viability (C), improved TEER (D), and decreased expression of VCAM1 and ICAM1 (E). One-way ANOVA followed by Bonferroni test; *, *P*<0.05. shVEGF_HG-CM, HG-CM from shVEGF-transfected Met-5A cells; NC_HG-CM, HG-CM from NC-transfected Met-5A cells. (F-J) Incubation of HPECs with VEGFR2i reduced the HG-CM-induced increase in ECAR (F) and PFKFB3 expression (G). This reduced glycolysis was associated with reduced cell viability (H), improved TEER (I), and decreased expression of VCAM1 and ICAM1 (J). One-way ANOVA followed by Bonferroni test; *, *P*<0.05. Error bars, mean ± SD (n=6 cell samples in each group).

**Supplemental Figure 12. Uncropped immunoblots**

**Supplemental Figure 13. Histological sections in Figure 3**

**Supplemental Table 1. Patient characteristics**

|  | ESKD  (n=3) | ESKD on PD  (n=3) | | *P* value |
| --- | --- | --- | --- | --- |
| Age (years) | 57±7 | 61±10 | 0.72 | |
| Scr (umol/L) | 763.0±241.1 | 864.7±236.6 | 0.63 | |
| Kt/V | / | 1.8±0.4 | / | |

ESKD on PD, ESKD patients with PD failure; ESKD, age- and serum creatinine-matched ESKD patients. Continuous data are expressed as mean ± SE. ESKD, end stage kidney disease; PD, peritoneal dialysis; Scr, serum creatinine; Kt/V, urea clearance index.

**Supplemental Table 2. List of shRNAs/ siRNAs used to knock down the indicated targets**

| **Target gene** | **sense** | **antisense** |
| --- | --- | --- |
| *PFKFB3* | GGGACUUGUCGCUGAUCAATT | UUGAUCAGCGACAAGUCCCTT |
| *VEGFR2* | GCAAGAGAAAUGAAUUUGUTT | ACAAAUUCAUUUCUCUUGCTT |
| *HIF1α* | GCCGAGGAAGAACUAUGAATT | UUCAUAGUUCUUCCUCGGCTT |
| *VEGFA* | GGAGUACCCUGAUGAGAUCTT | GAUCUCAUCAGGGUACUCCTT |

**Supplemental Table 3. List of primers used for real-time PCR**

| **Gene** | **Forward (5’->3’)** | **Reverse (5’->3’)** |
| --- | --- | --- |
| *PFKFB3* | CGACCCCGACAAATGCGACAG | GTACACGATGCGGCTCTGGATG |
| *PFKP* | GCATGGGTATCTACGTGGGG | CTCTGCGATGTTTGAGCCTC |
| *HK1* | GCTCTCCGATGAAACTCTCATAG | GGACCTTACGAATGTTGGCAA |
| *LDHA* | ATGGCAACTCTAAAGGATCAGC | CCAACCCCAACAACTGTAATCT |
| *KDR* | AGGGAGTCTGTGGCATCTGAAGG | GTGGTGTCTGTGTCATCGGAGTG |
| *ICAM1* | GTCACCTATGGCAACGACTCCTTC | AGTGTCTCCTGGCTCTGGTTCC |
| *VCAM1* | TCTCATTGACTTGCAGCACCACAG | CCTCATTCGTCACCTTCCCATTCAG |
| *SELE* | GCACATCTCAGGGACAATGGACAG | CATCCTTCAGGACAGGCGAACTTG |
| *HIF1A* | AGTTCCGCAAGCCCTGAAAGC | GCAGTGGTAGTGGTGGCATTAGC |
| *VEGFA* | CTGCTGTAACGATGAAGCCCTG | GCTGTAGGAAGCTCATCTCTCC |
| *ACTB* | GCACTCTTCCAGCCTTCCTTCC | GCGGATGTCCACGTCACACTTC |
| *Pfkfb3* | CGCTCCACACTGTCCTGAAACTG | GGAAGGTCTGACTACACTGGCAAAG |
| *Pfkp* | GAAACATGAGGCGTTCTGTGT | CCCGGCACATTGTTGGAGA |
| *Hk1* | CGGAATGGGGAGCCTTTGG | GCCTTCCTTATCCGTTTCAATGG |
| *Aldoa* | CGTGTGAATCCCTGCATTGG | CAGCCCCTGGGTAGTTGTC |
| *Eno2* | AGGTGGATCTCTATACTGCCAAA | GTCCCCATCCCTTAGTTCCAG |
| *Ldha* | TGTCTCCAGCAAAGACTACTGT | GACTGTACTTGACAATGTTGGGA |
| *G6pdx* | CACAGTGGACGACATCCGAAA | AGCTACATAGGAATTACGGGCAA |
| *Aprt* | CCCTCTTGAAAGACCCGGAC | TCCAGAGAATAGGAGGCTGAC |
| *Pnp* | ATCTGTGGTTCCGGCTTAGGA | TGGGGAAAGTTGGGTATCTCAT |
| *Uck2* | CTTCCGTTTGTGCTAAGATCGT | GTGAGGACTCGGTAGAAGCTAT |
| *Vegfa* | ACCCACGACAGAAGGAGAGCAG | CACAGGACGGCTTGAAGATGTACTC |
| *Icam1* | GTGATGCTCAGGTATCCATCCA | CACAGTTCTCAAAGCACAGCG |
| *Vcam1* | GCTTCAAGAGGGTGGTGCTGTG | GCAATTAAGGTGAGGGTGGCATTTC |
| *Sele* | GCTCCAGGTGAACCAAACAACAAAC | CGTCATTCCACATGCCCGAGTC |
| *Pgam1* | TCTGTGCAGAAGAGAGCAATCC | CTGTCAGACCGCCATAGTGT |
| *Hk1* | CGGAATGGGGAGCCTTTGG | GCCTTCCTTATCCGTTTCAATGG |
| *Hk2* | TGATCGCCTGCTTATTCACGG | AACCGCCTAGAAATCTCCAGA |
| *Hk3* | CTGAGTCAAGGCTGTATCCTCC | TGCACCAGTTCAGCATCTGAGG |
| *Col1a1* | CCTCAGGGTATTGCTGGACAAC | CAGAAGGACCTTGTTTGCCAGG |
| *Fn1* | CCCTATCTCTGATACCGTTGTCC | CCCTATCTCTGATACCGTTGTCC |
| *Actb* | GTGACGTTGACATCCGTAAAGA | GCCGGACTCATCGTACTCC |
