# Supplementary figures and images for: Hyperglycolysis in endothelial cells drives endothelial injury and microvascular alterations in peritoneal dialysis
Source: Clin Transl Med. 2023 Nov 30;13(12):e1498. doi: 10.1002/ctm2.1498 (PMC10689974; doi:10.1002/ctm2.1498)

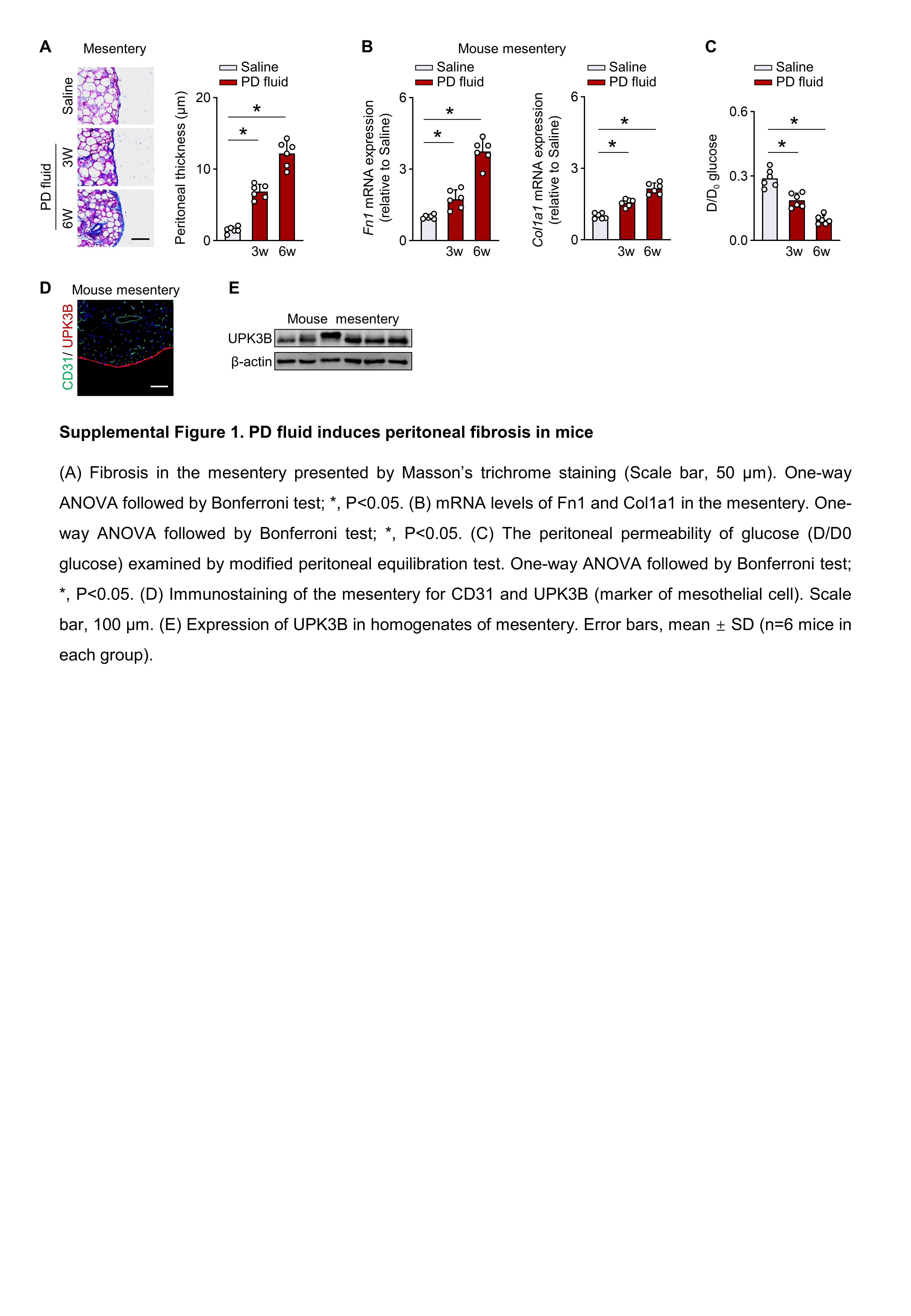

Supplement: Supplementary file 2 — Supporting Information Figure S1 PD fluid induces peritoneal fibrosis in mice. [file CTM2-13-e1498-s008.docx]

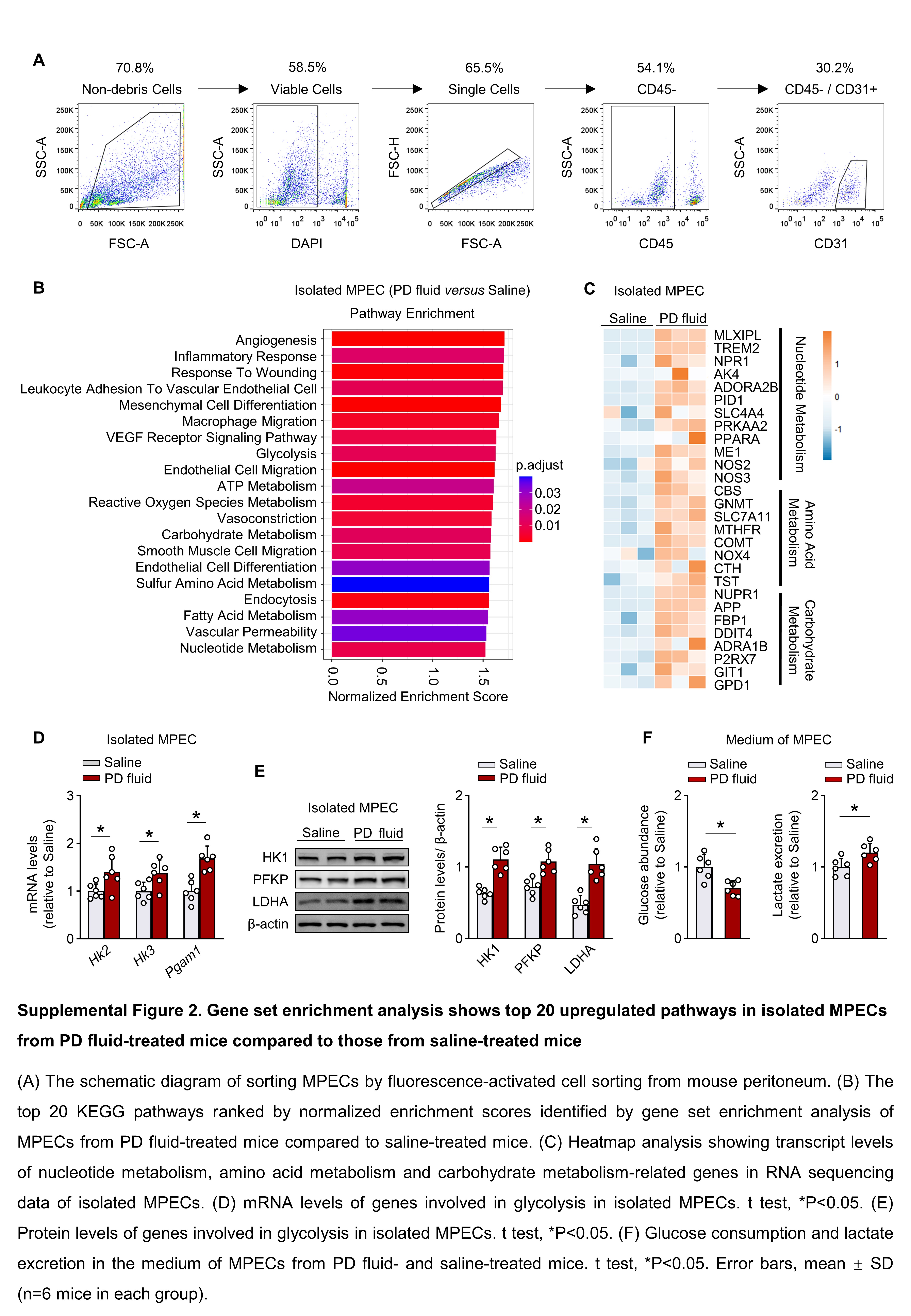

Supplement: Supplementary file 3 — Supporting Information Figure S2 Gene set enrichment analysis shows top 20 upregulated pathways in isolated MPECs from PD fluid‐treated mice compared to those from saline‐treated mice. [file CTM2-13-e1498-s003.docx]

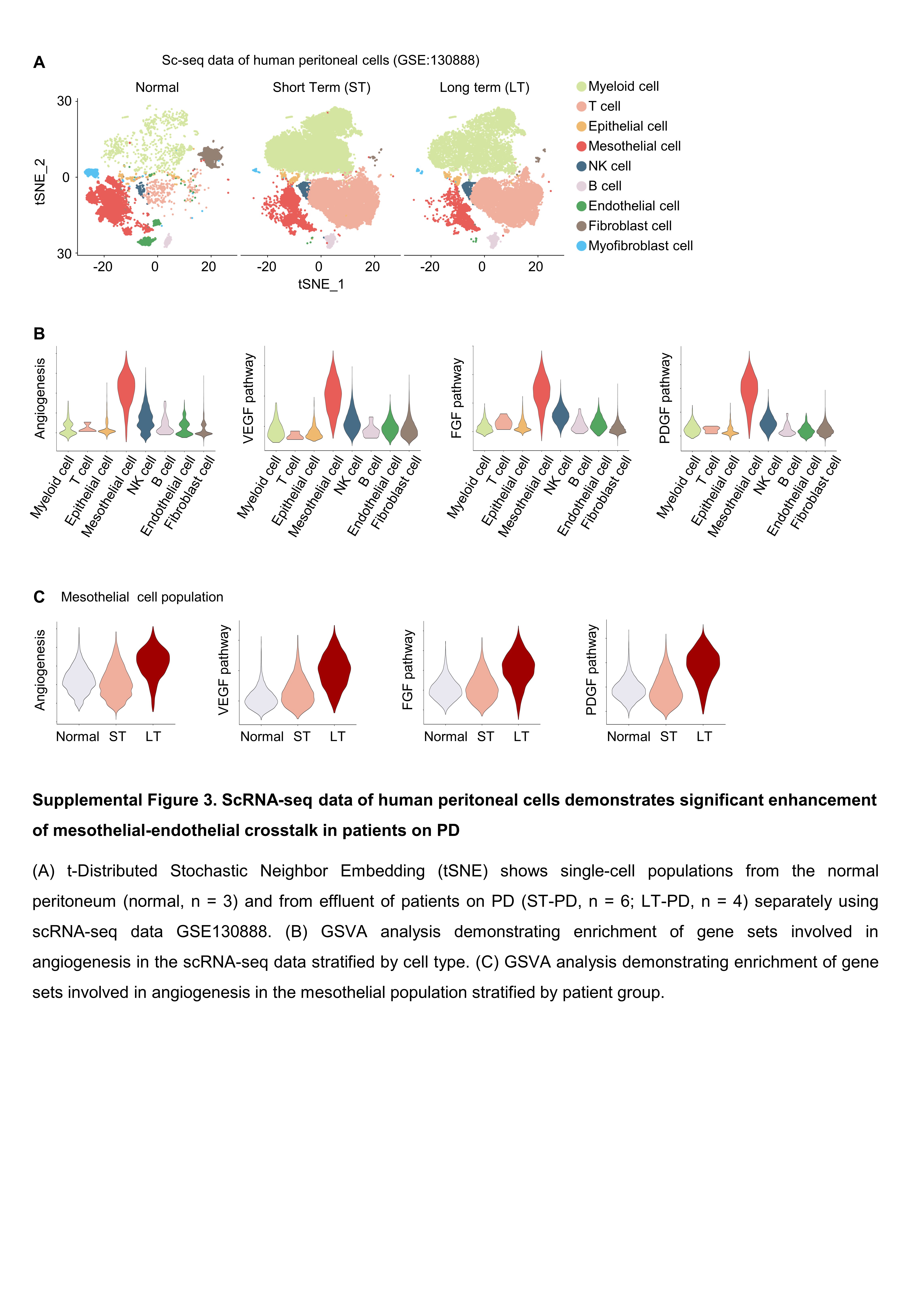

Supplement: Supplementary file 4 — Supporting Information Figure S3 ScRNA‐seq data of human peritoneal cells demonstrate significant enhancement of mesothelial–endothelial crosstalk in patients on PD. [file CTM2-13-e1498-s002.docx]

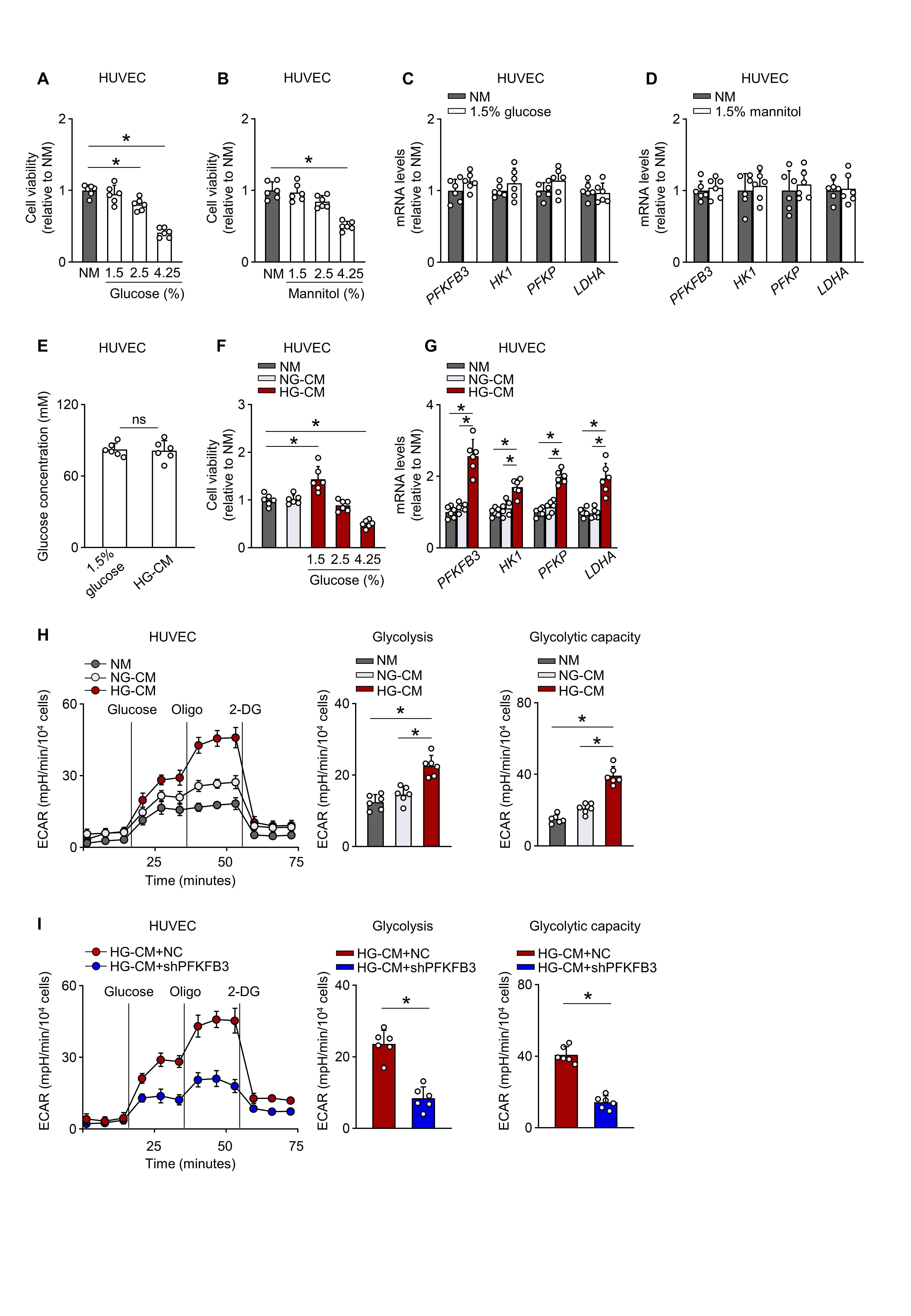


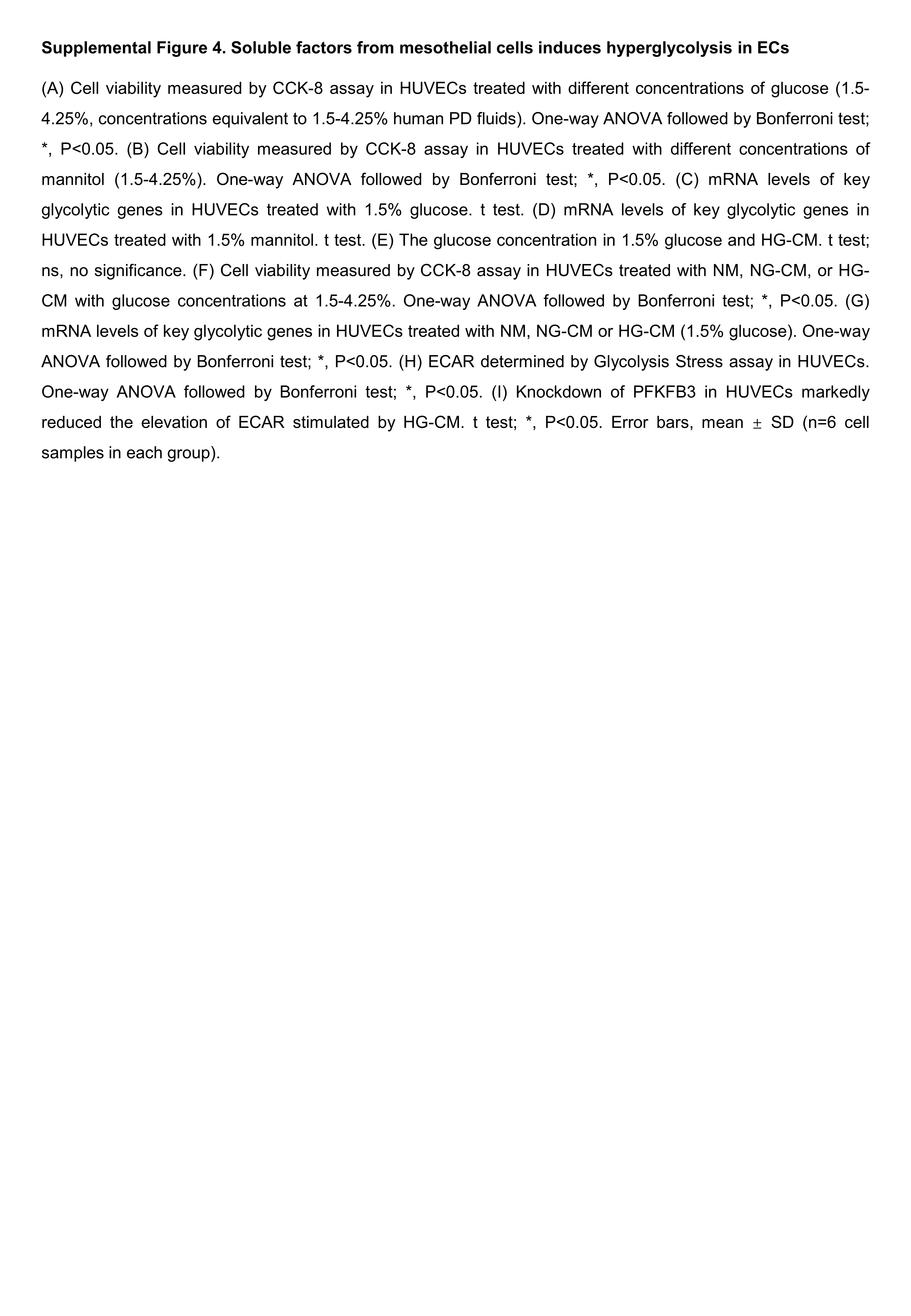

Supplement: Supplementary file 5 — Supporting Information Figure S4 Soluble factors from mesothelial cells induce hyperglycolysis in ECs. [file CTM2-13-e1498-s012.docx]

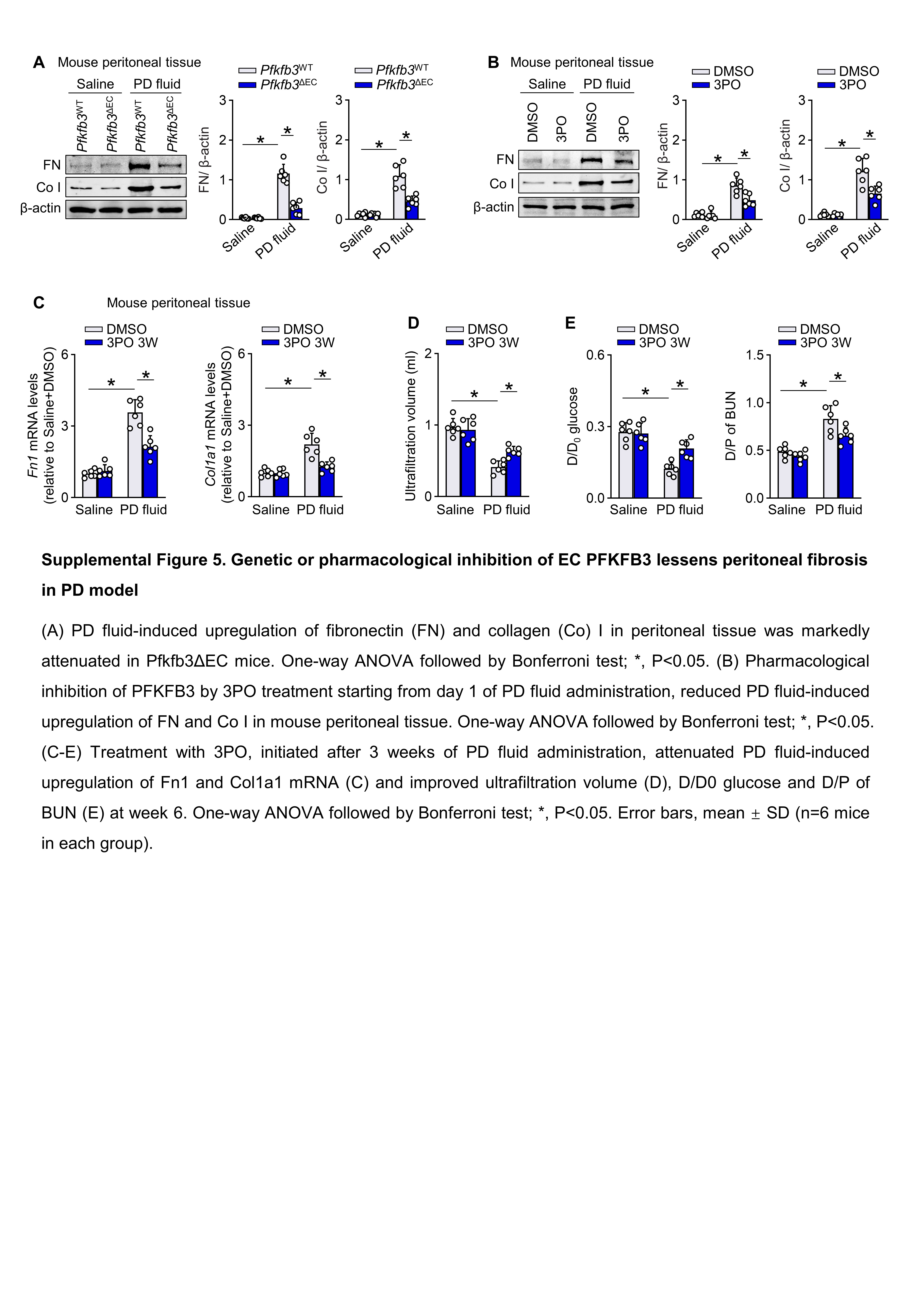

Supplement: Supplementary file 6 — Supporting Information Figure S5 Genetic or pharmacological inhibition of EC PFKFB3 lessens peritoneal fibrosis in PD model. [file CTM2-13-e1498-s009.docx]

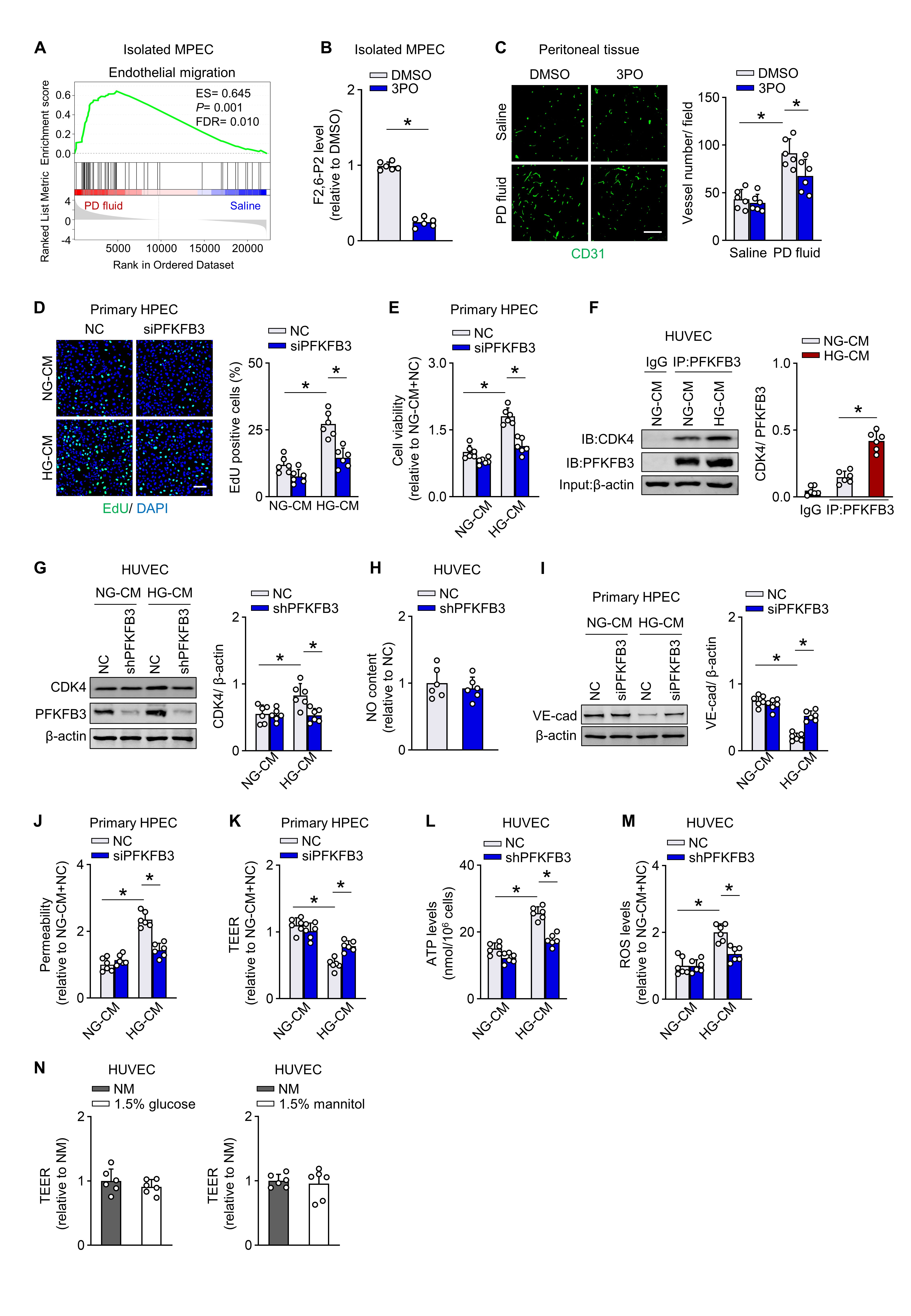


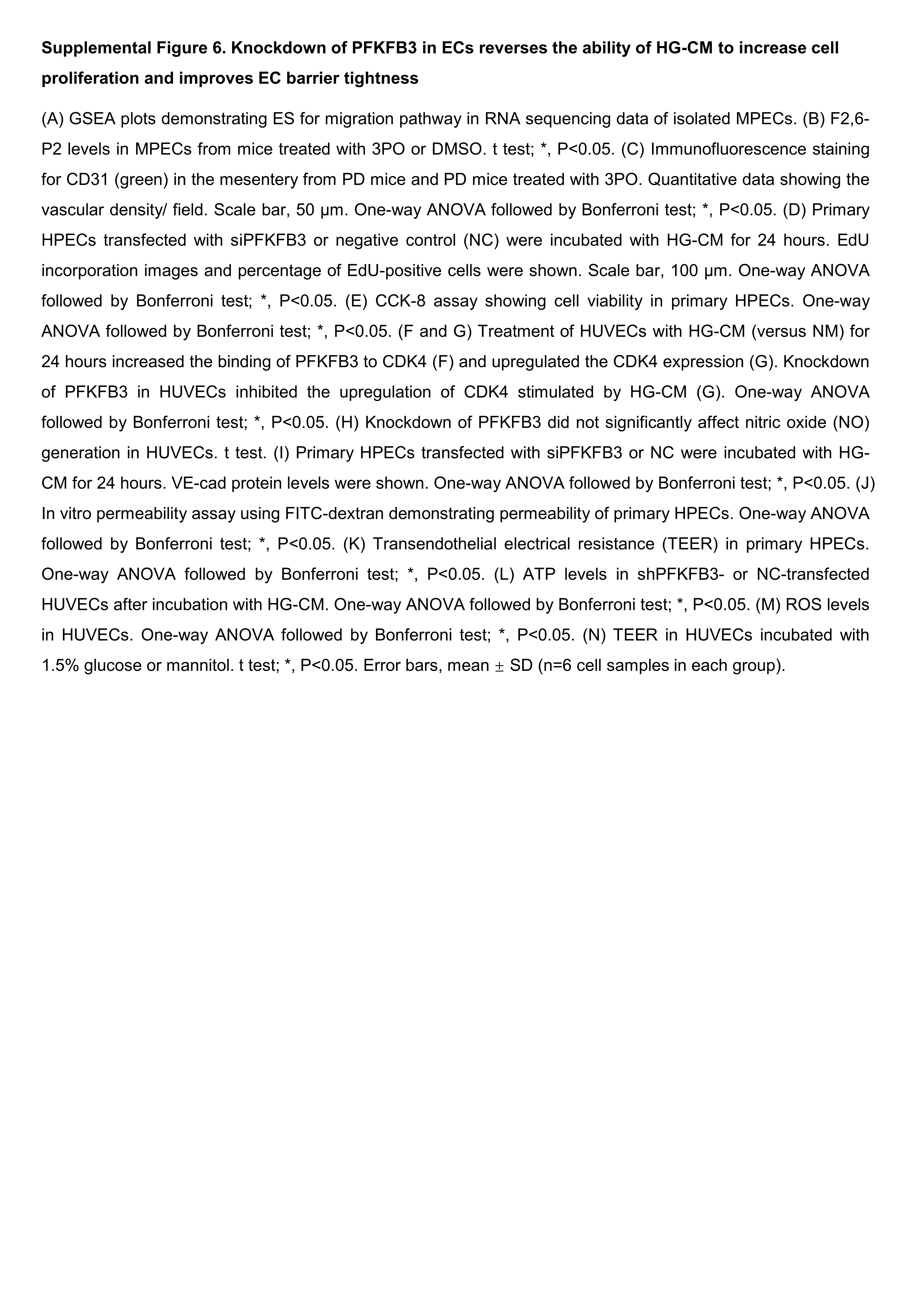

Supplement: Supplementary file 7 — Supporting Information Figure S6 Knockdown of PFKFB3 in ECs reverses the ability of HG‐CM to increase cell proliferation and improves EC barrier tightness. [file CTM2-13-e1498-s011.docx]

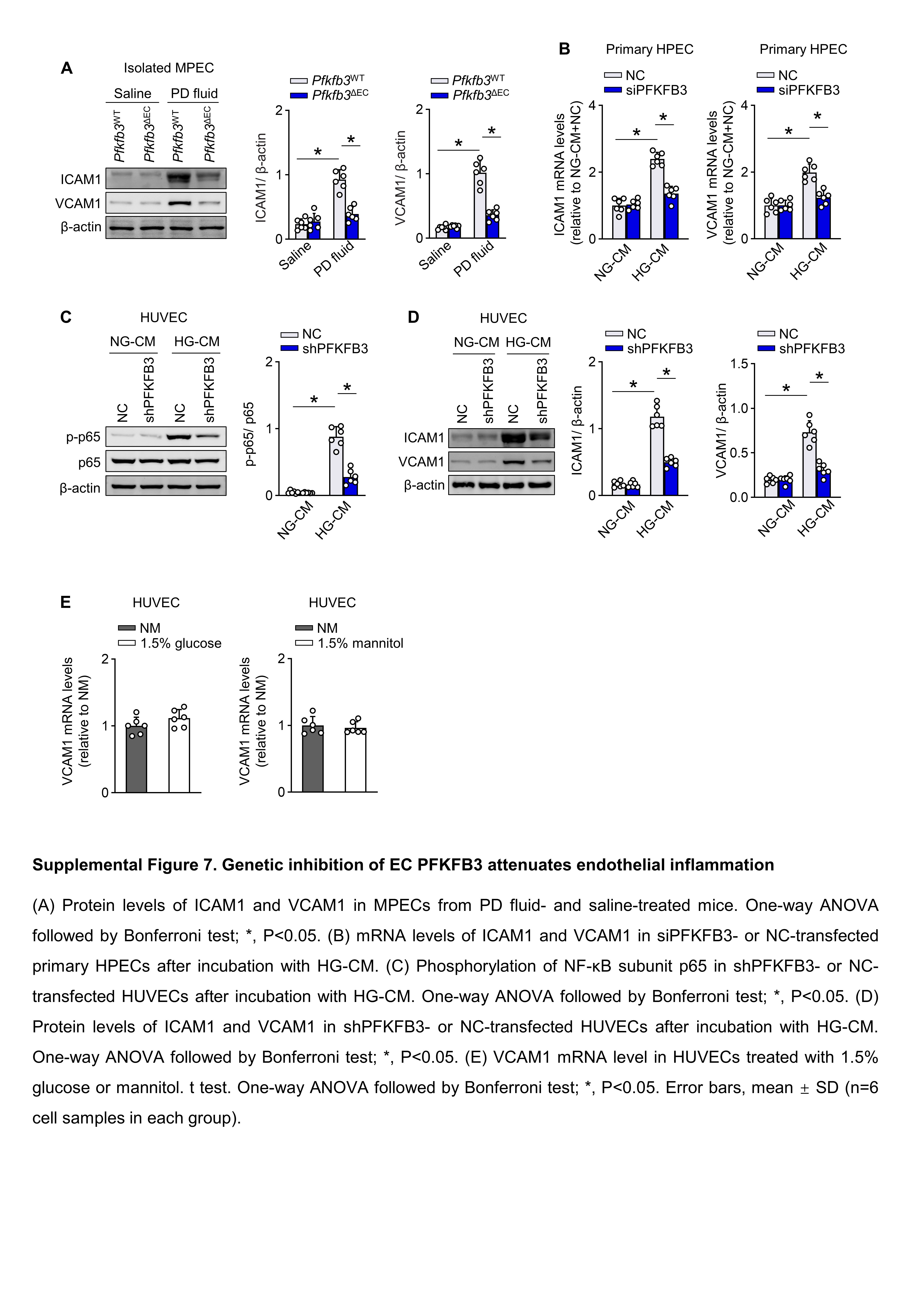

Supplement: Supplementary file 8 — Supporting Information Figure S7 Genetic inhibition of EC PFKFB3 attenuates endothelial inflammation. [file CTM2-13-e1498-s001.docx]

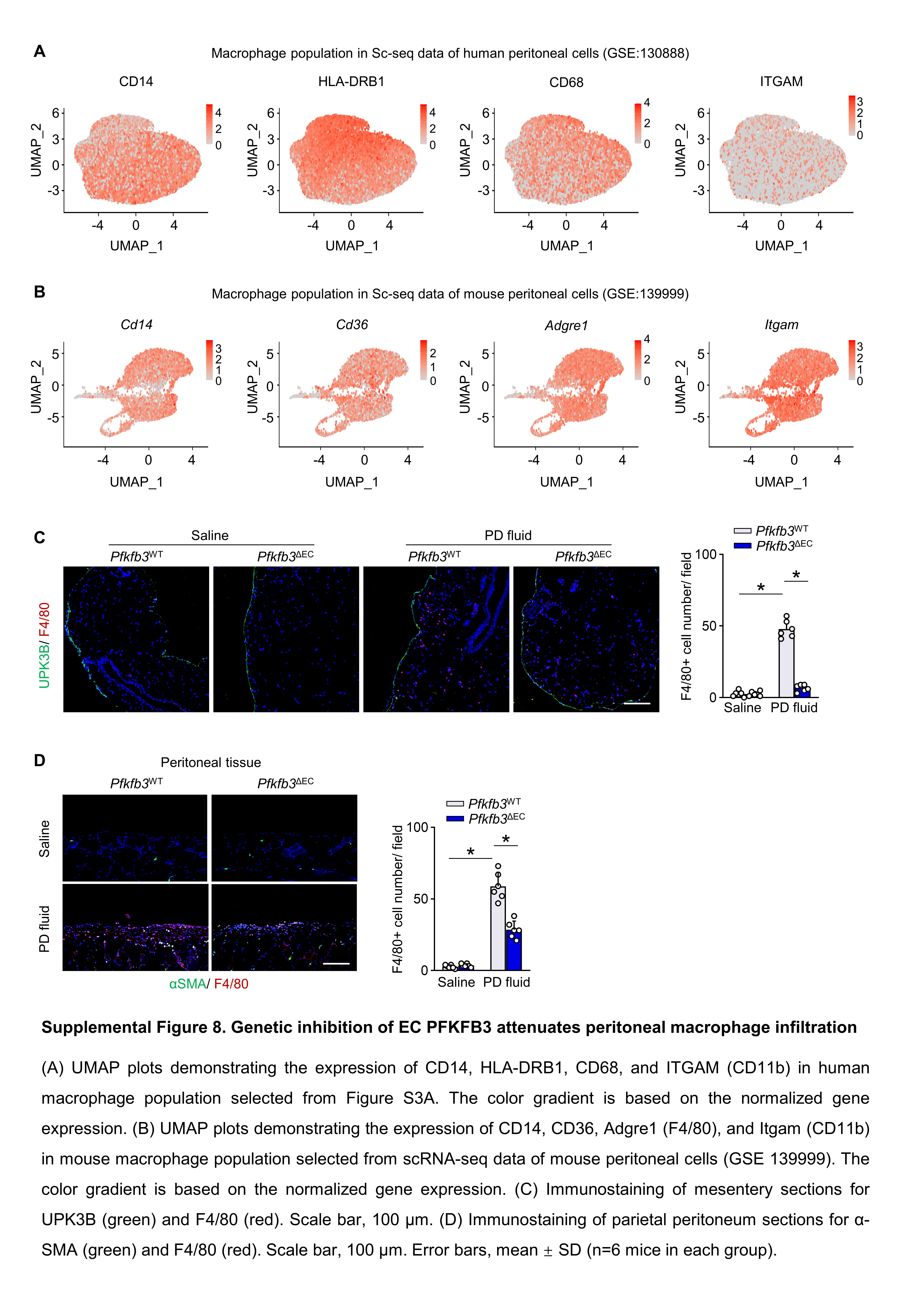

Supplement: Supplementary file 9 — Supporting Information Figure S8 Genetic inhibition of EC PFKFB3 attenuates peritoneal macrophage infiltration. [file CTM2-13-e1498-s007.docx]

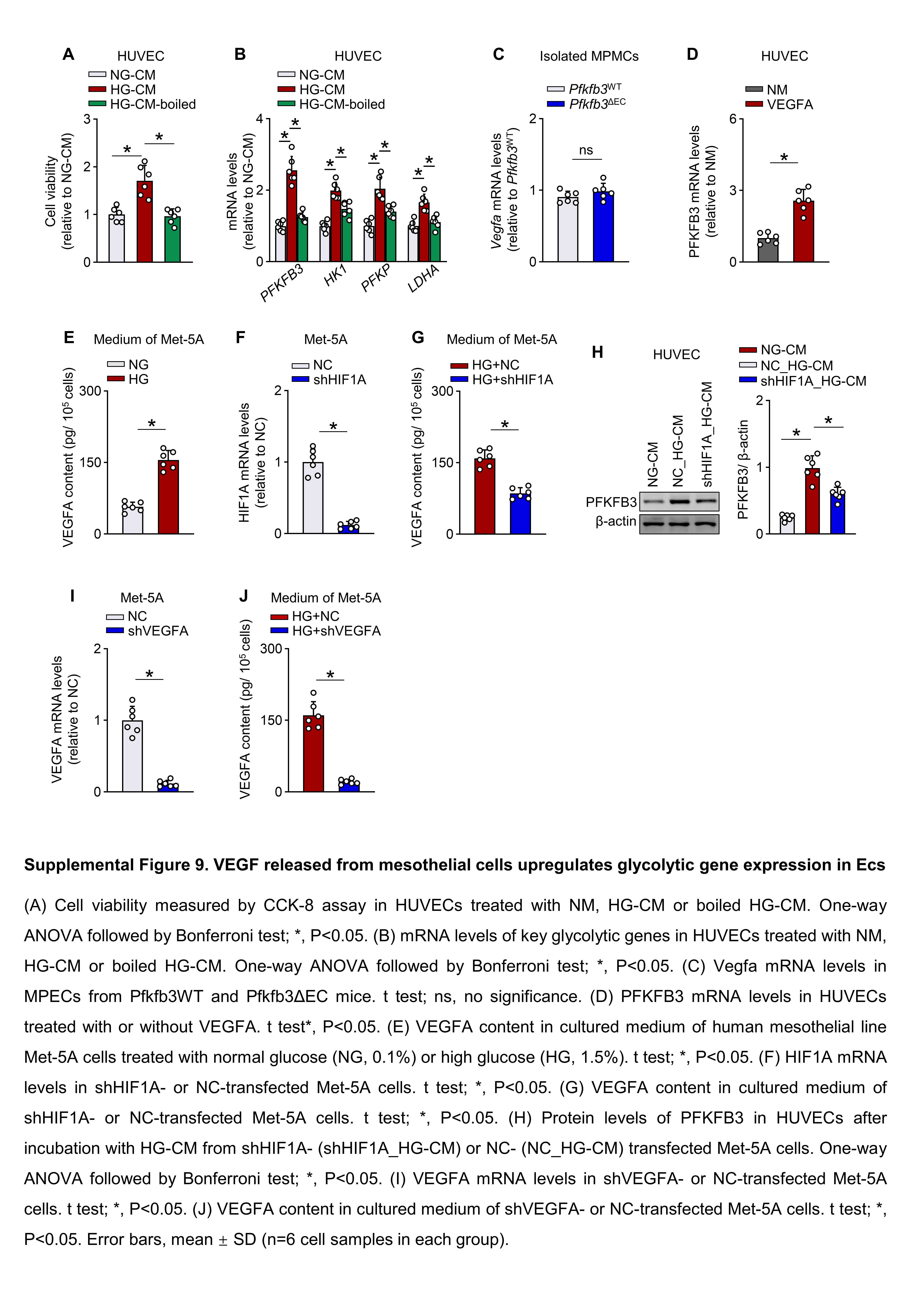

Supplement: Supplementary file 10 — Supporting Information Figure S9 VEGF released from mesothelial cells upregulates glycolytic gene expression in ECs. [file CTM2-13-e1498-s006.docx]

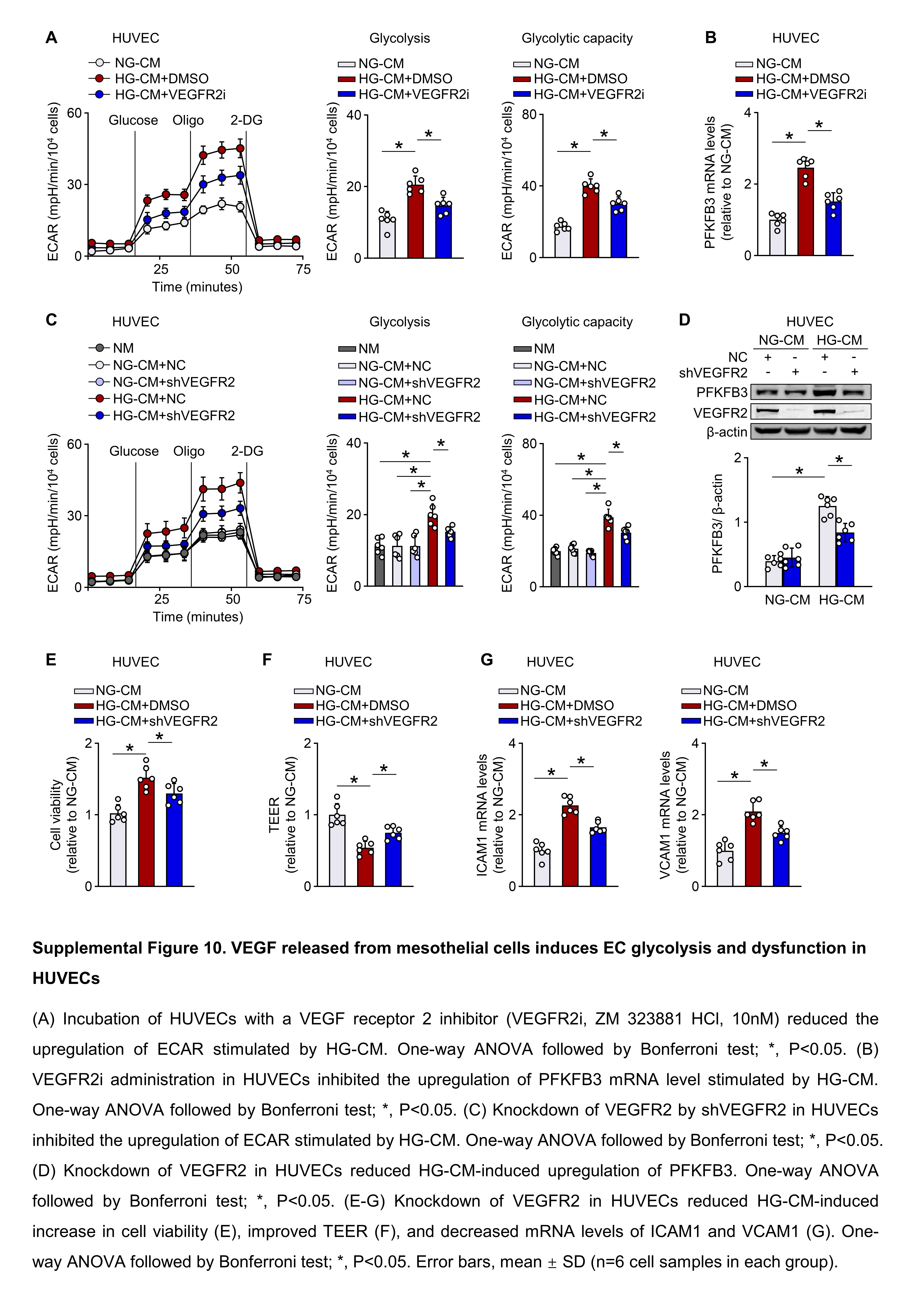

Supplement: Supplementary file 11 — Supporting Information Figure S10 VEGF released from mesothelial cells induces EC glycolysis and dysfunction in HUVECs. [file CTM2-13-e1498-s013.docx]

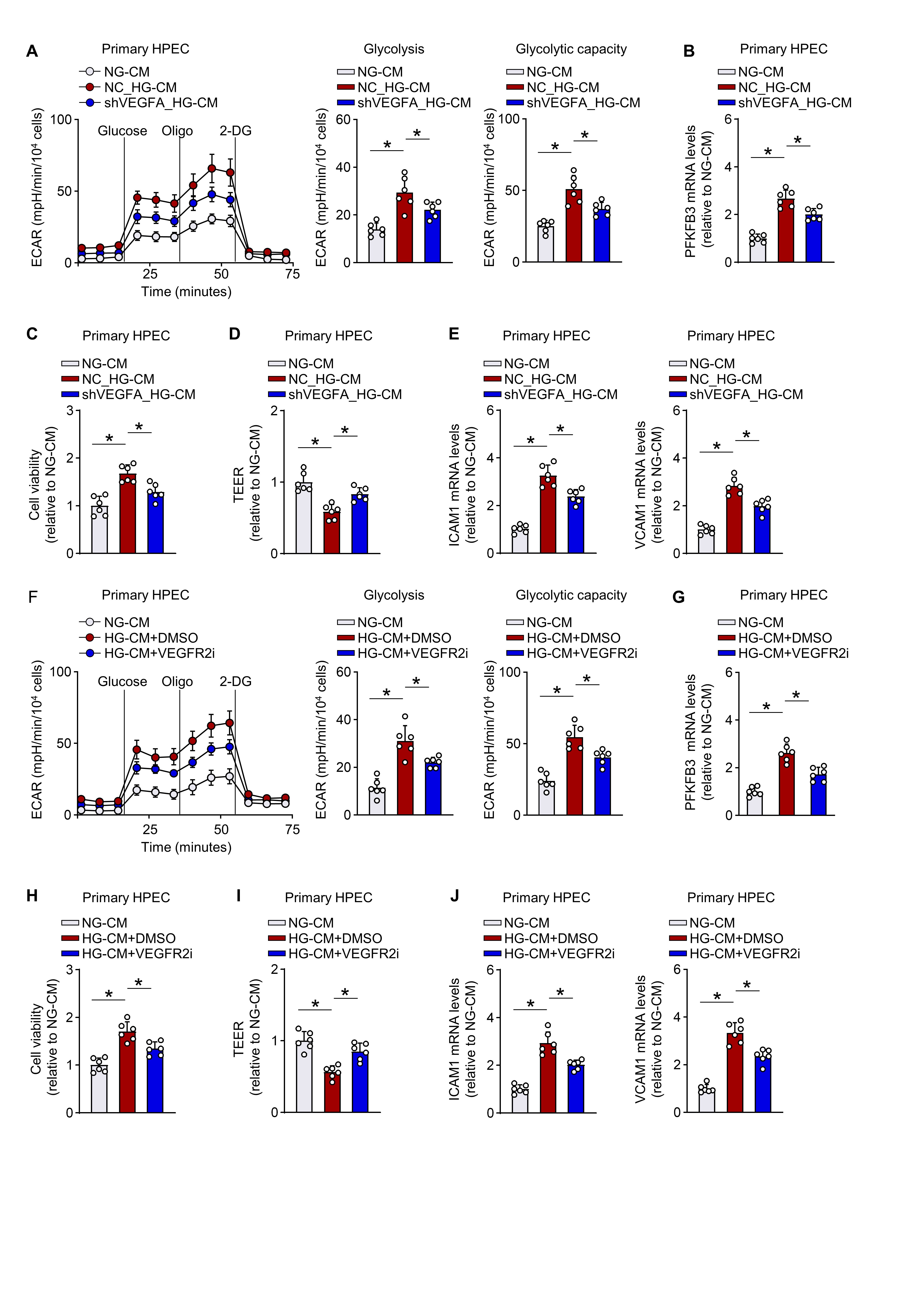


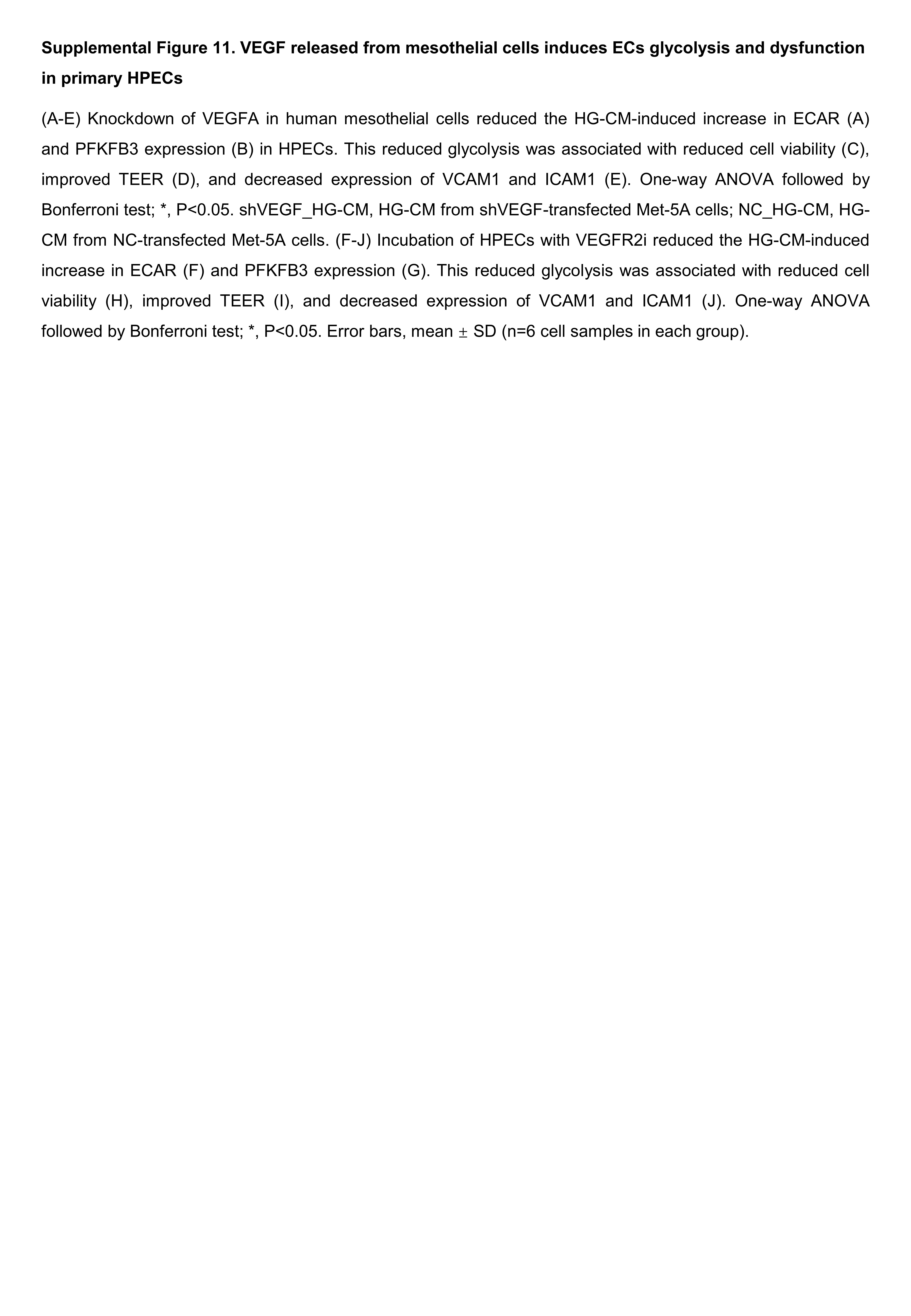

Supplement: Supplementary file 12 — Supporting Information Figure S11 VEGF released from mesothelial cells induces ECs glycolysis and dysfunction in primary HPECs. [file CTM2-13-e1498-s004.docx]

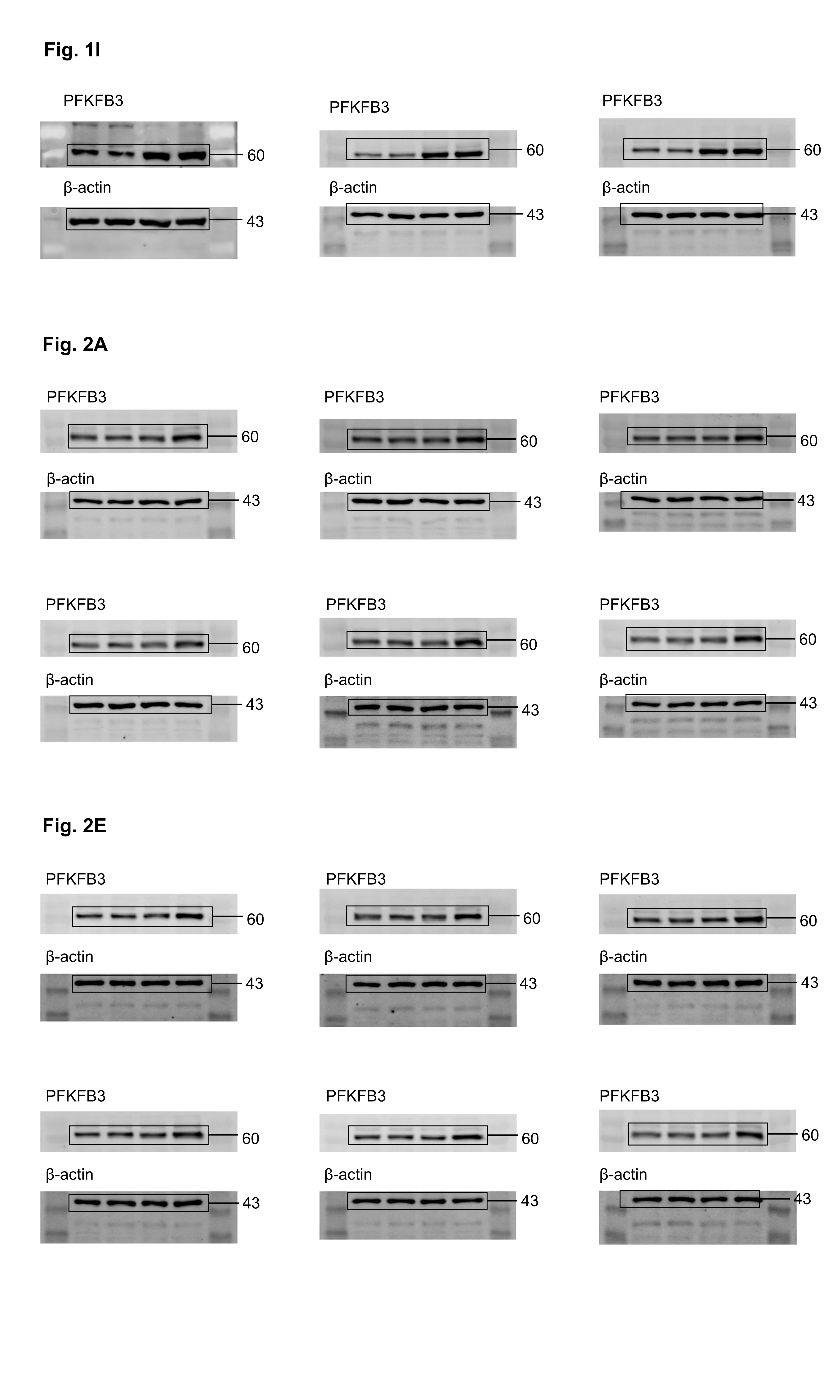


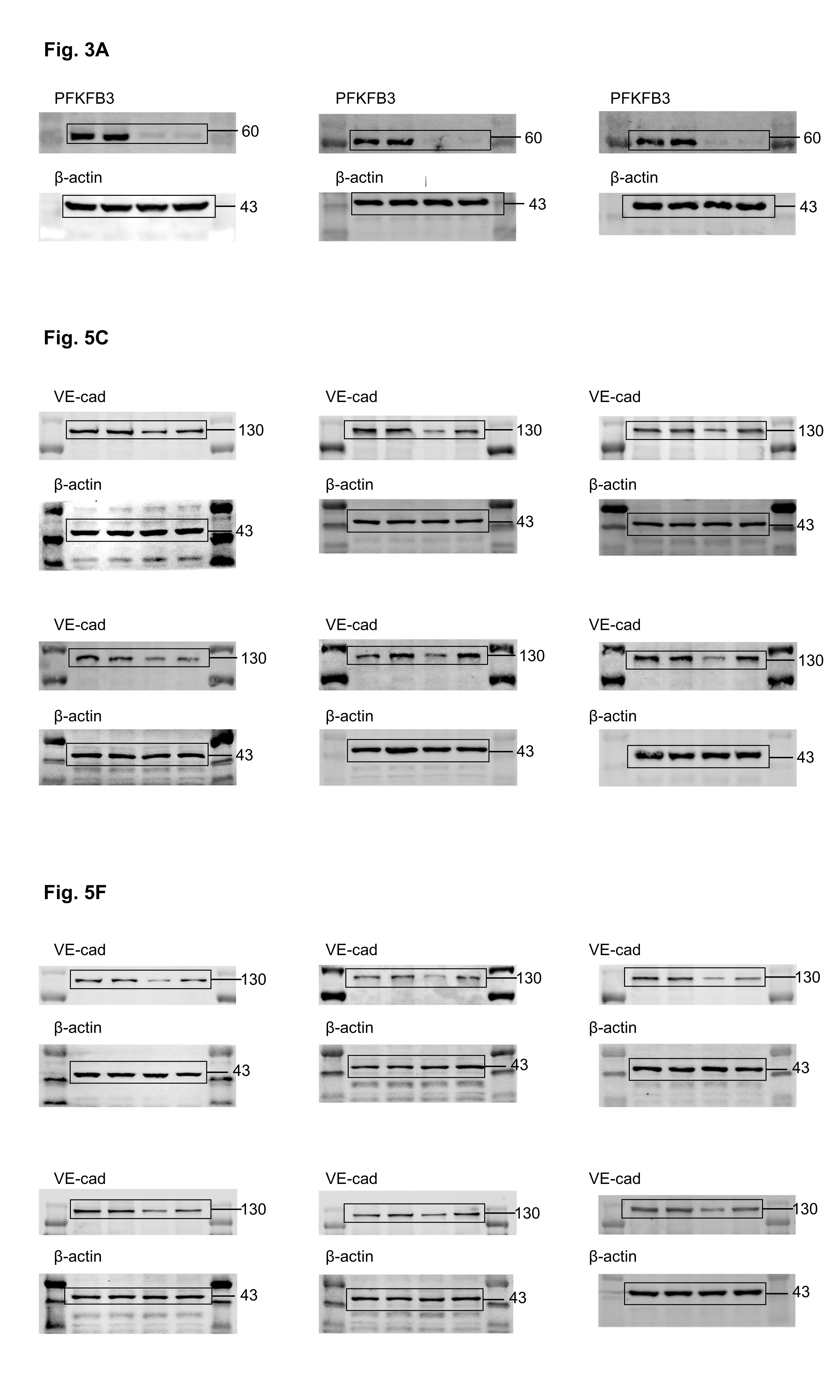


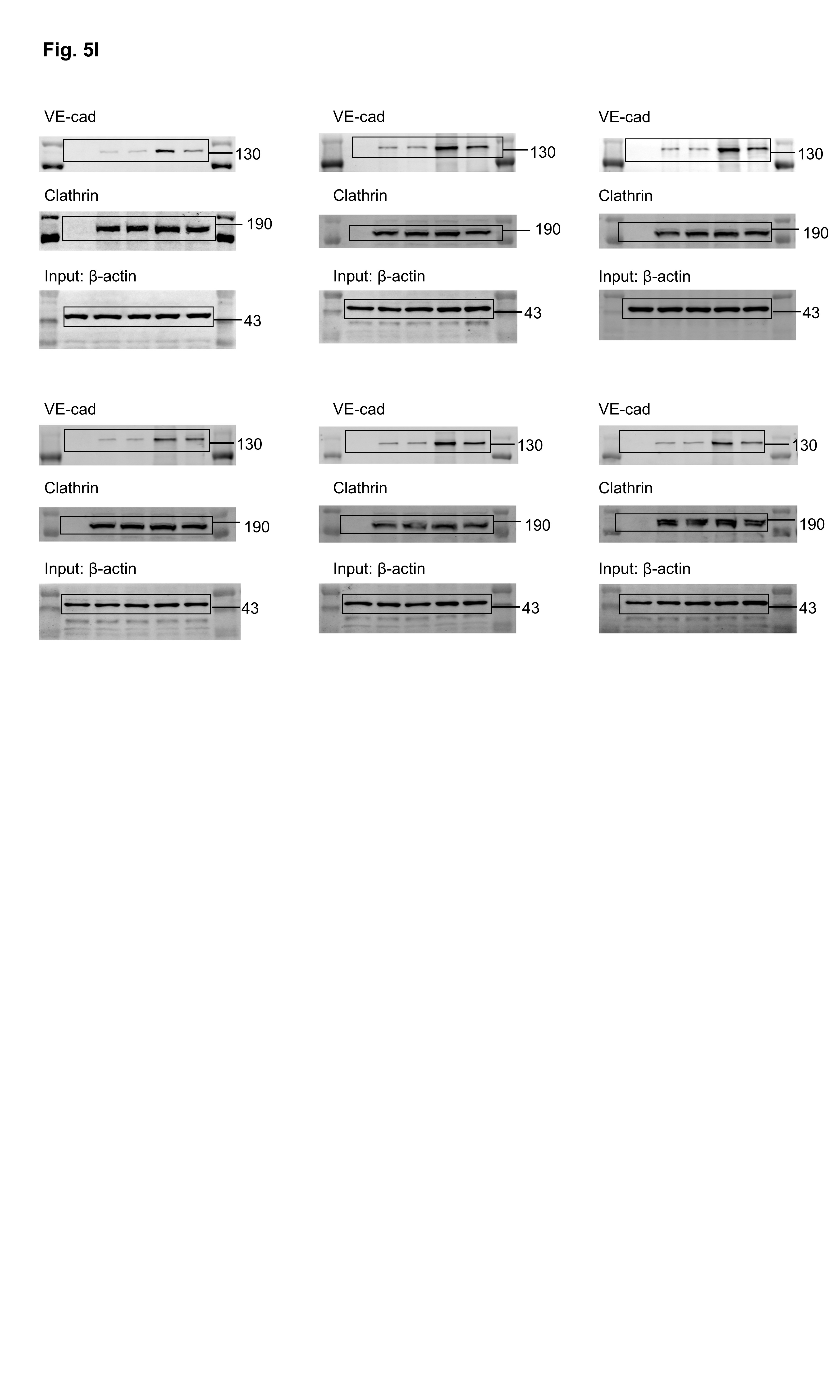


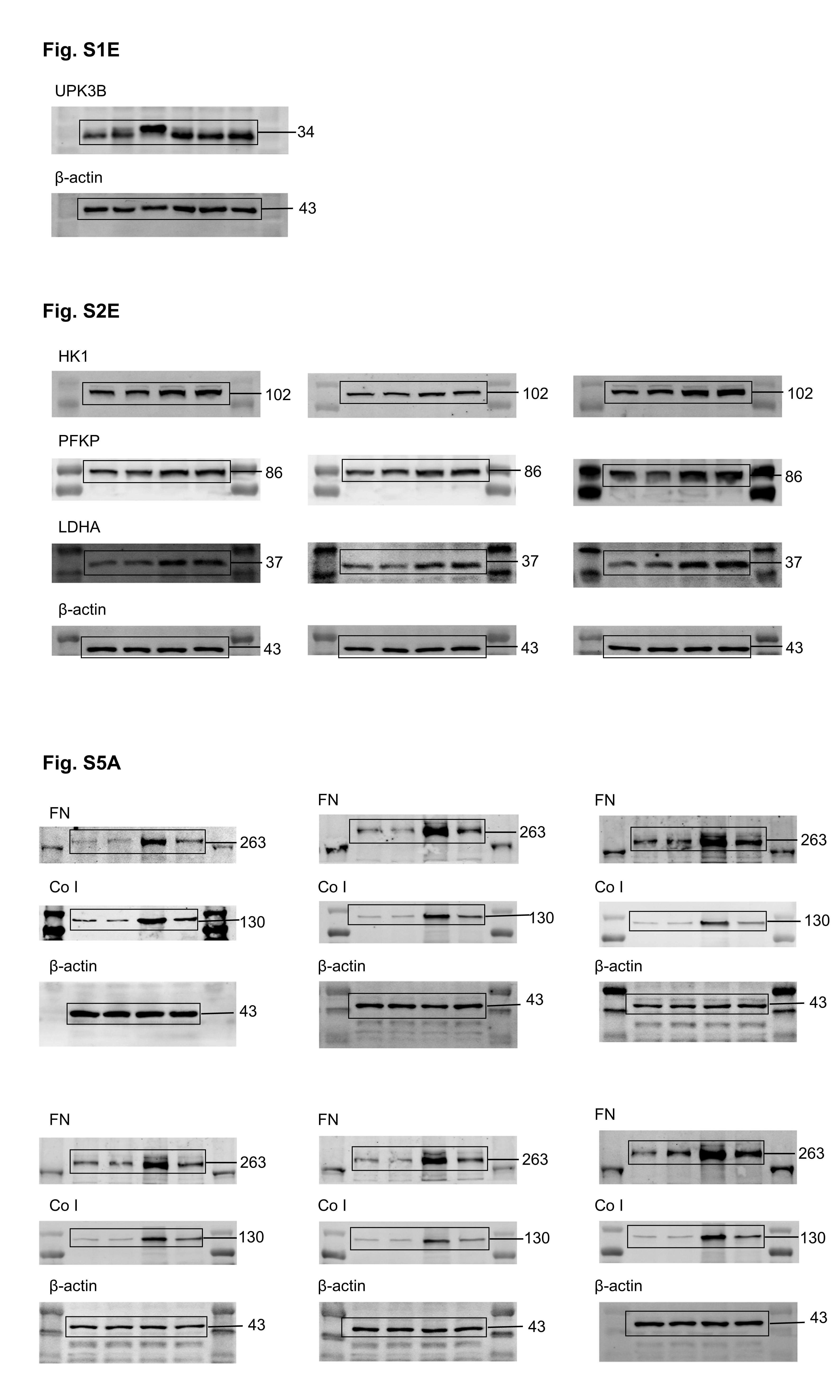


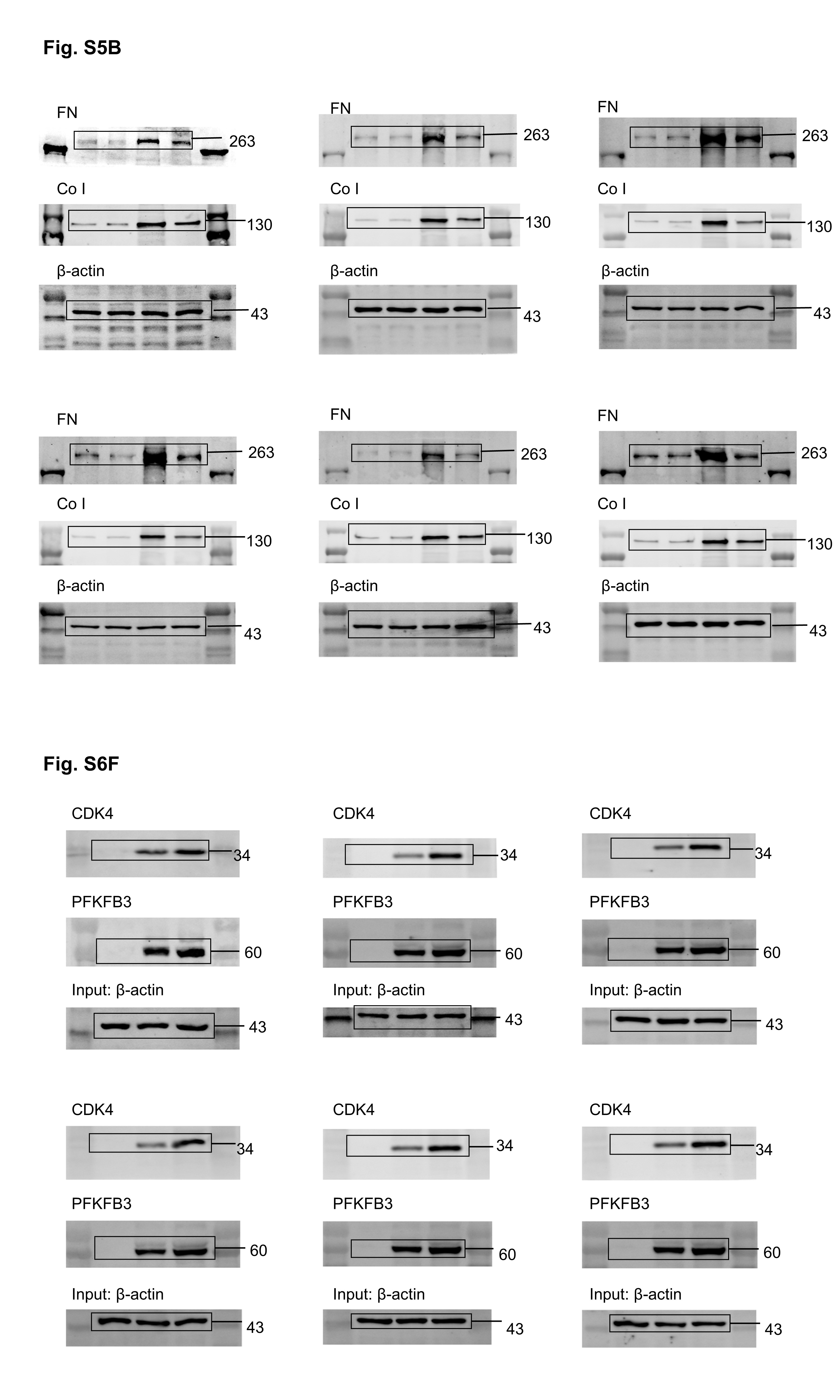


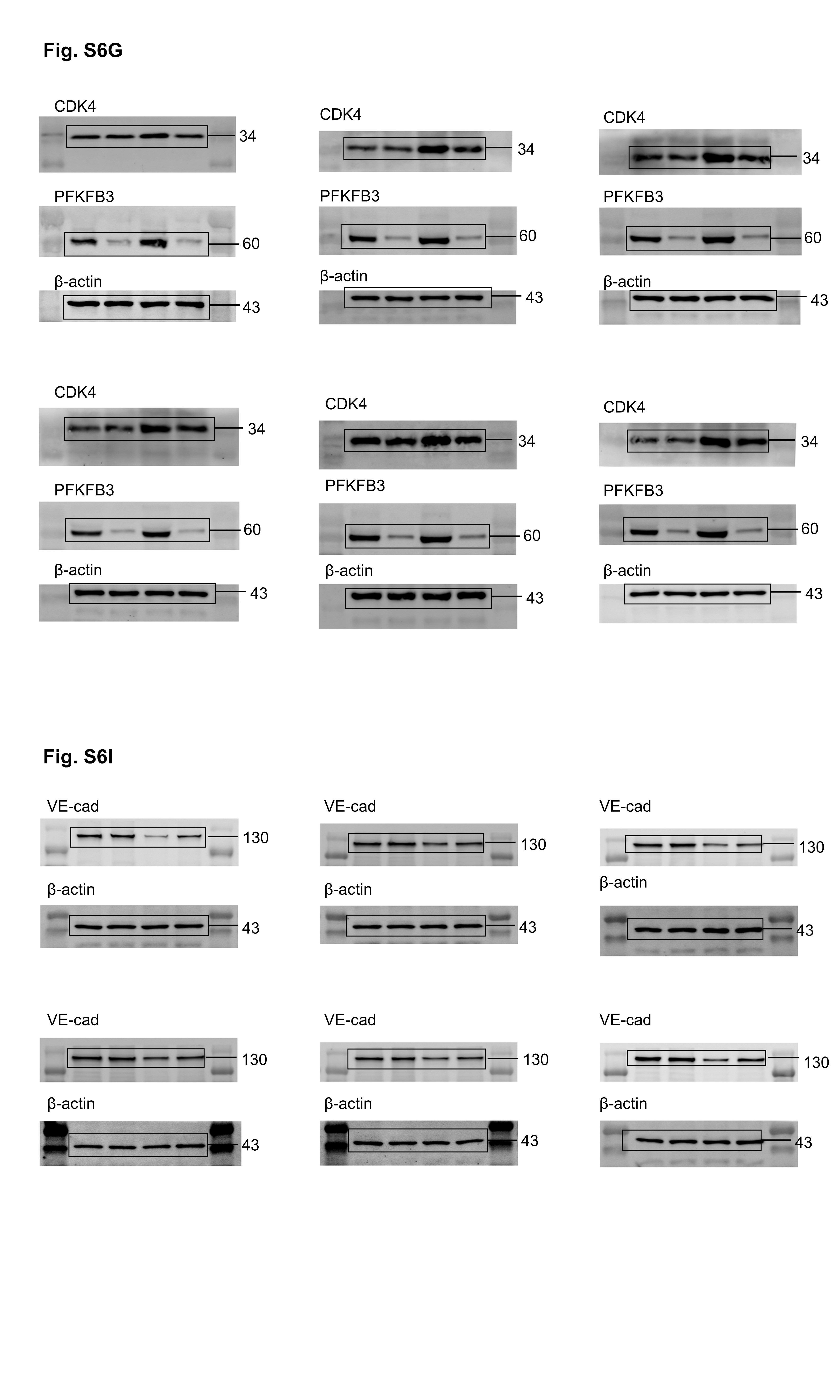


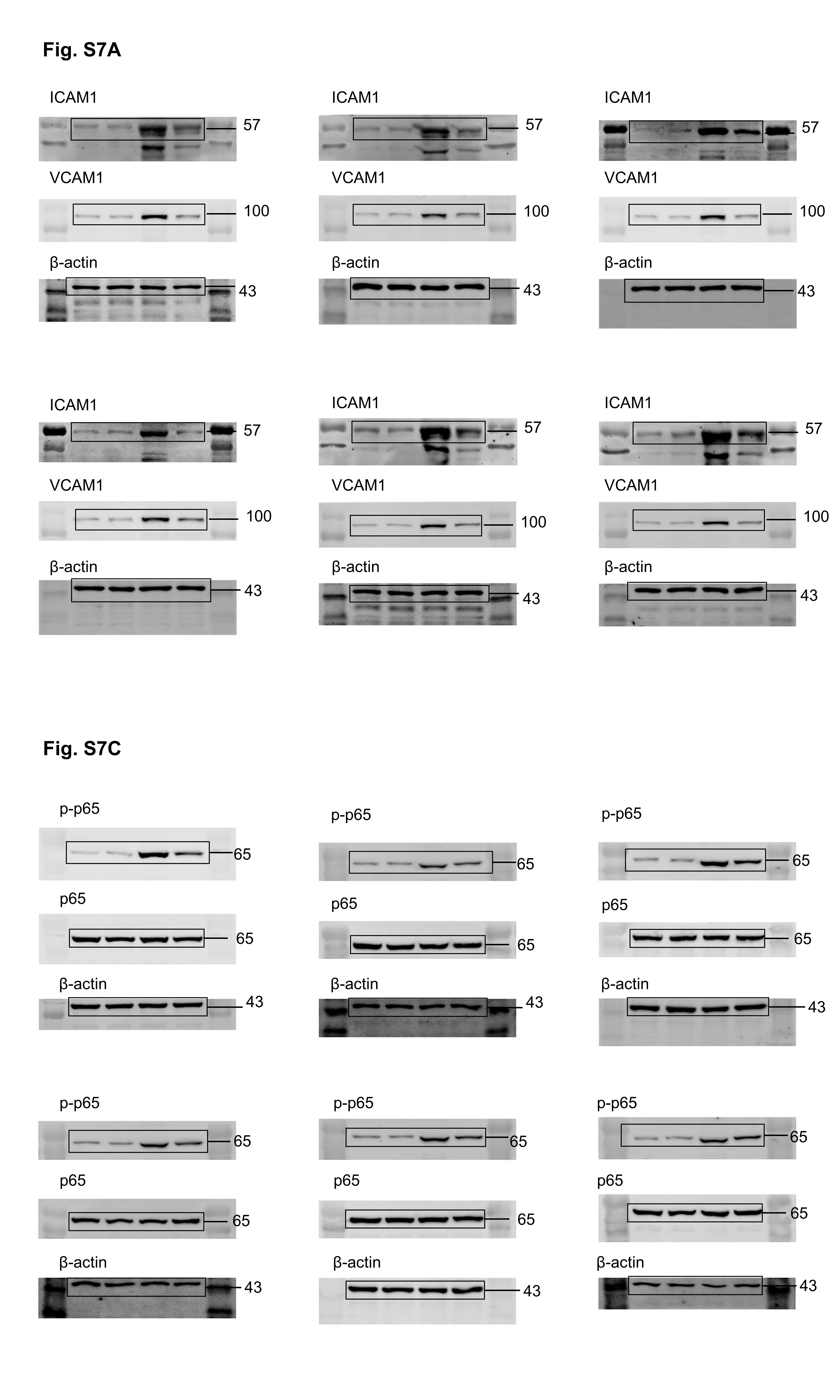


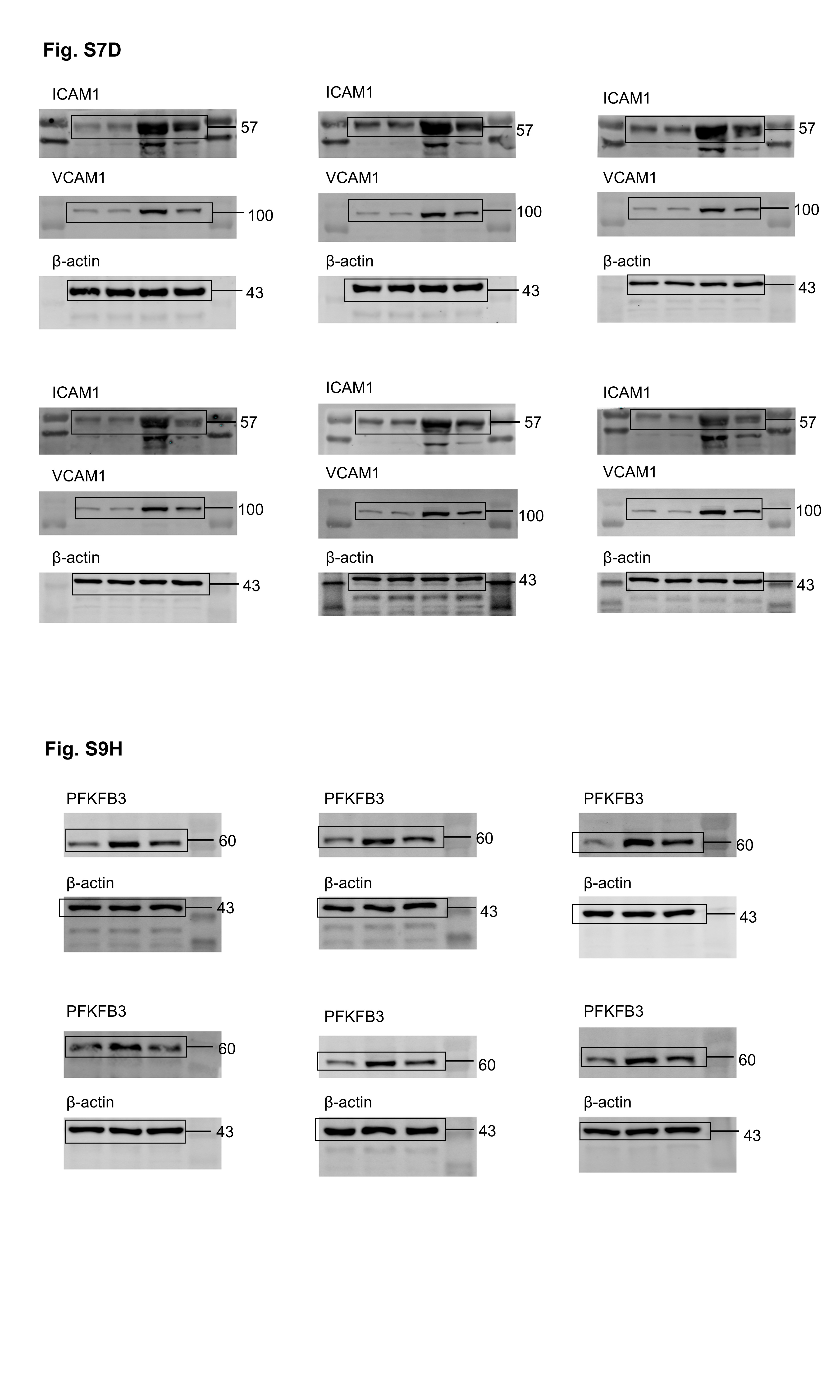


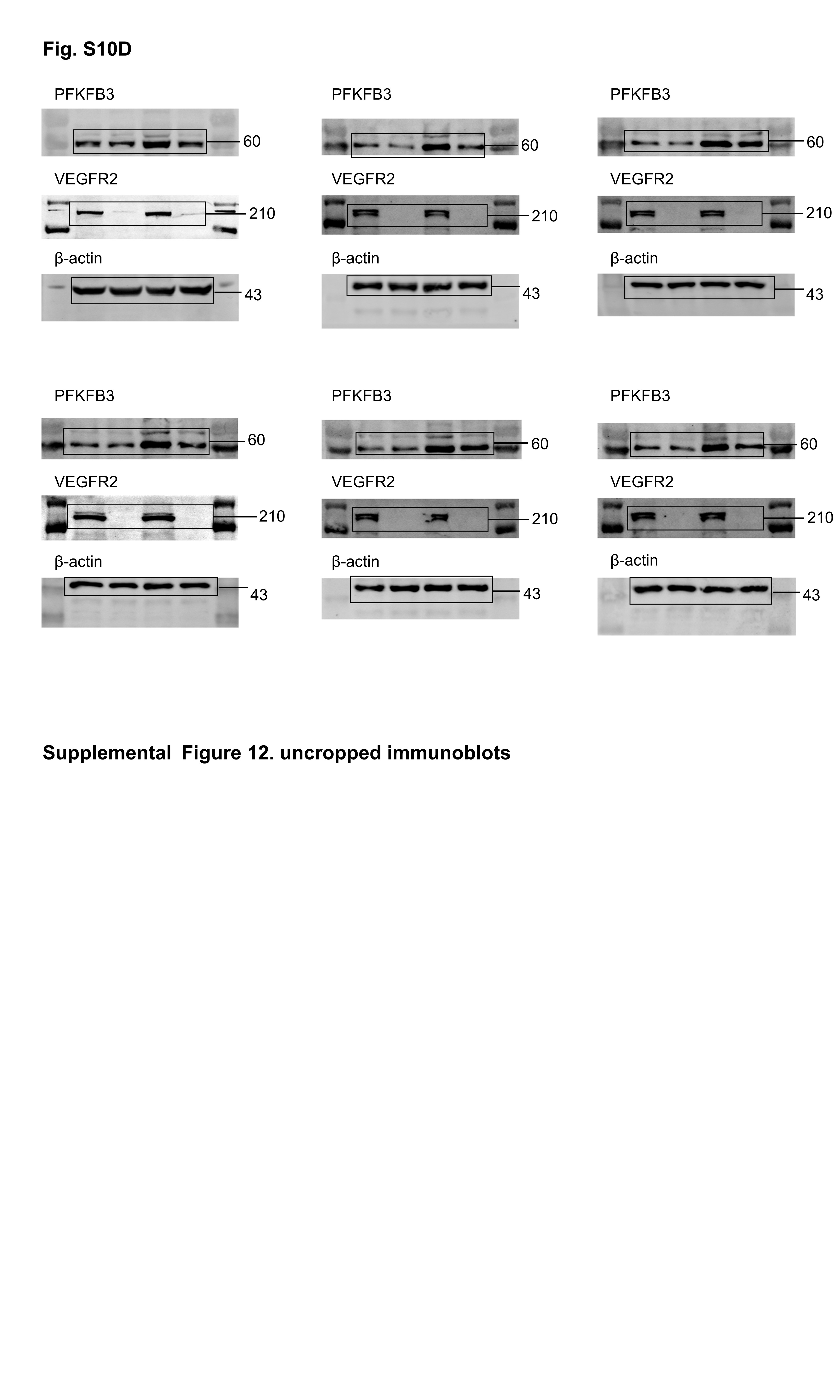

Supplement: Supplementary file 13 — Supporting Information Figure S12 Uncropped immunoblots. [file CTM2-13-e1498-s010.docx]

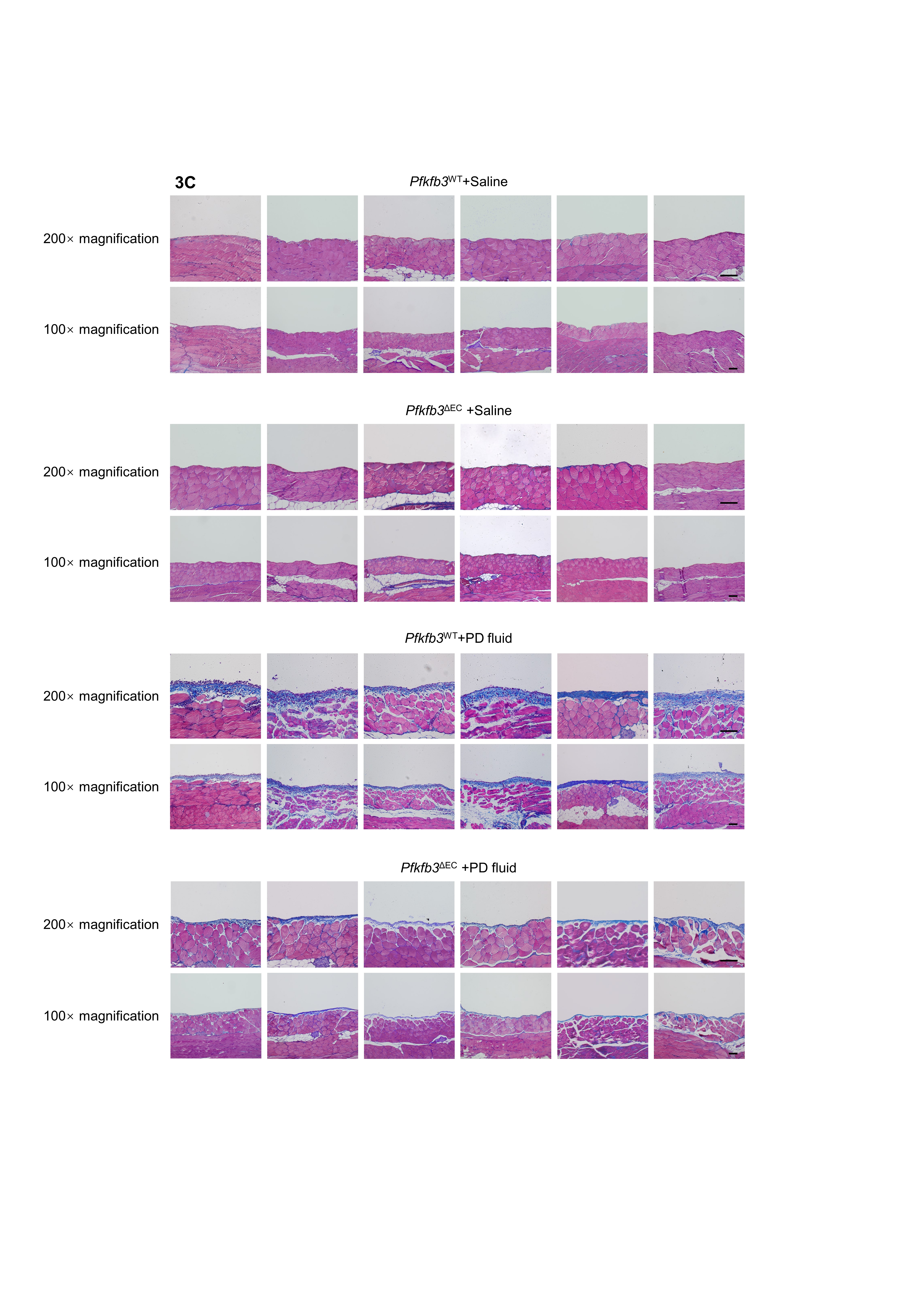


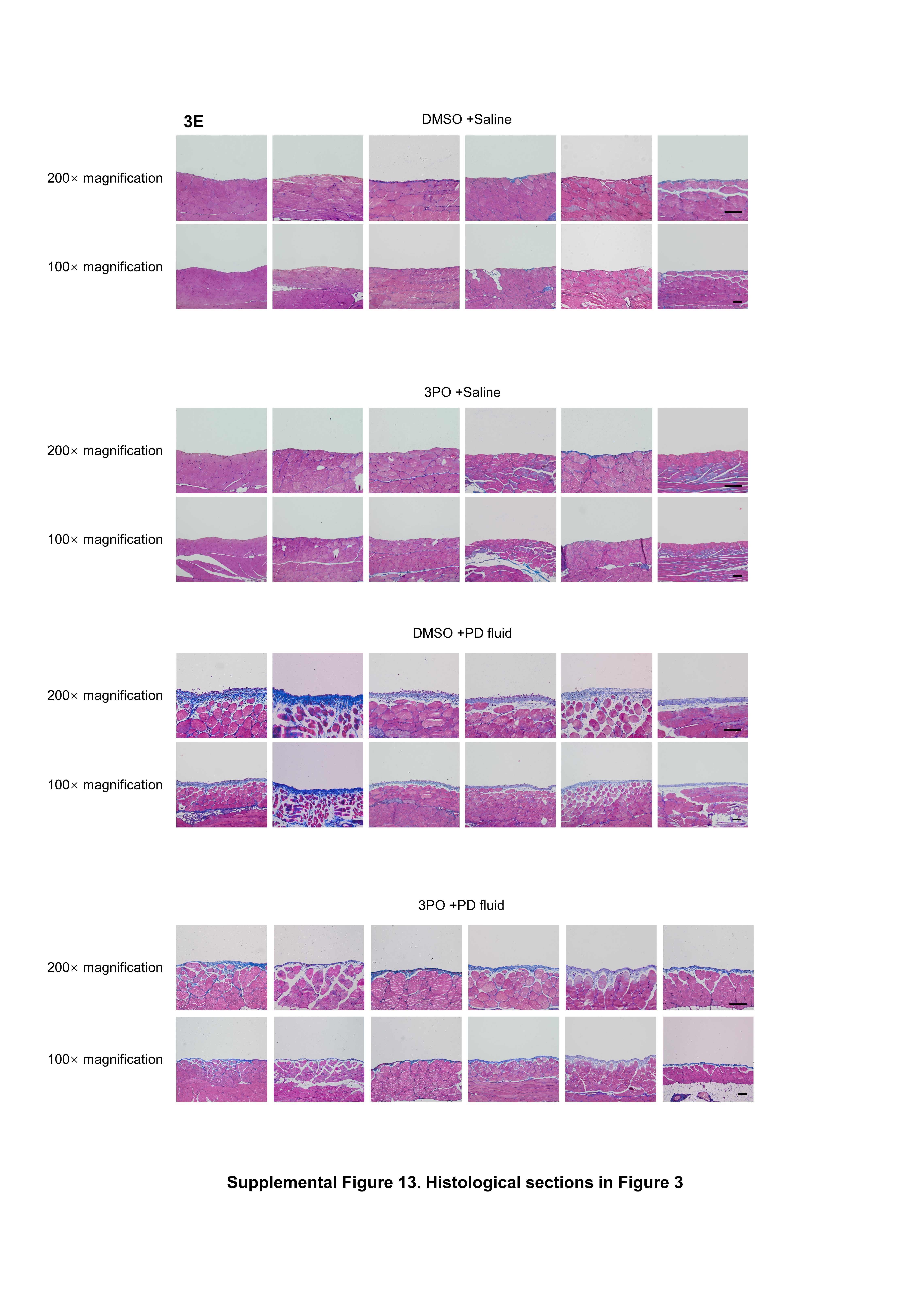

Supplement: Supplementary file 14 — Supporting Information Figure S13 Histological sections in Figure 3. [file CTM2-13-e1498-s005.docx]
